# Evaluating Intensity, Complexity, and Potential for Causal Inference in Social Needs Interventions: A Review of a Scoping Review

**DOI:** 10.1001/jamanetworkopen.2024.17994

**Published:** 2024-06-21

**Authors:** Meera Viswanathan, Sara M. Kennedy, Nila Sathe, Michelle L. Eder, Valerie Ng, Shannon Kugley, Megan A. Lewis, Laura M. Gottlieb

**Affiliations:** 1 RTI International, Research Triangle Park, North Carolina; 2 Kaiser Permanente Center for Health Research, Portland, Oregon; 3 Department of Family and Community Medicine, University of California, San Francisco

## Abstract

**Importance:**

Interventions that address needs such as low income, housing instability, and safety are increasingly appearing in the health care sector as part of multifaceted efforts to improve health and health equity, but evidence relevant to scaling these social needs interventions is limited.

**Objective:**

To summarize the intensity and complexity of social needs interventions included in randomized clinical trials (RCTs) and assess whether these RCTs were designed to measure the causal effects of intervention components on behavioral, health, or health care utilization outcomes.

**Evidence Review:**

This review of a scoping review was based on a Patient-Centered Outcomes Research Institute–funded evidence map of English-language US-based RCTs of social needs interventions published between January 1, 1995, and April 6, 2023. Studies were assessed for features related to intensity (defined using modal values as providing as-needed interaction, 8 participant contacts or more, contacts occurring every 2 weeks or more often, encounters of 30 minutes or longer, contacts over 6 months or longer, or home visits), complexity (defined as addressing multiple social needs, having dedicated staff, involving multiple intervention components or practitioners, aiming to change multiple participant behaviors [knowledge, action, or practice], requiring or providing resources or active assistance with resources, and permitting tailoring), and the ability to assess causal inferences of components (assessing interventions, comparators, and context).

**Findings:**

This review of a scoping review of social needs interventions identified 77 RCTs in 93 publications with a total of 135 690 participants. Most articles (68 RCTs [88%]) reported 1 or more features of high intensity. All studies reported 1 or more features indicative of high complexity. Because most studies compared usual care with multicomponent interventions that were moderately or highly dependent on context and individual factors, their designs permitted causal inferences about overall effectiveness but not about individual components.

**Conclusions and Relevance:**

Social needs interventions are complex, intense, and include multiple components. Our findings suggest that RCTs of these interventions address overall intervention effectiveness but are rarely designed to distinguish the causal effects of specific components despite being resource intensive. Future studies with hybrid effectiveness-implementation and sequential designs, and more standardized reporting of intervention intensity and complexity could help stakeholders assess the return on investment of these interventions.

## Key Points

**Question:**

What is the intensity and complexity of different social needs interventions, and what is the potential for causal inference about specific intervention components?

**Findings:**

This review of a scoping review examined 77 randomized clinical trials of social needs interventions; the majority (68 studies [88%]) described features indicating high intervention intensity and all studies reported features indicating high intervention complexity. Study designs permitted conclusions on overall effectiveness but typically did not permit casual inferences about individual intervention components.

**Meaning:**

These findings suggest that social needs–related interventions undertaken in health care settings are often complex and intensive and have generally not been designed to assess the causal effects of specific components.

## Teaser

This review of a scoping review analyzes the intensity and complexity of social needs interventions in randomized clinical trials and whether the trials were designed to assess the effect of individual intervention components on health outcomes.

## Introduction

Intervening to improve social determinants of health—“the conditions in which people are born, grow up, live, work and age”^1^—has increasingly been heralded as critical to improving health equity.^2,3,4,5^ Social determinants include both upstream structural and societal systems, policies, and norms and the downstream manifestations of those upstream factors, such as the day-to-day availability of food, transportation, housing, and safety. These downstream manifestations of social adversity are often referred to as social risks.^6^ More recently, the health care sector has begun to support health care activities focused on reducing social risks (ie, social care or social needs interventions).^6^ New state and federal policies are designed to incentivize the uptake of these interventions.^7,8,9,10^

The value of social needs interventions needs to be assessed to understand the extent of health care sector involvement. Although several studies have suggested that social needs interventions undertaken in health care settings may improve health outcomes^11^ without increasing (and sometimes even lowering) health care costs,^12,13,14,15^ the added value of individual intervention components has received relatively little attention. By design, randomized clinical trials (RCTs) assess the effect of interventions on measured outcomes, but major barriers to uptake of social needs interventions include the lack of resources to implement and sustain these programs.^16,17^ As a result, assessing value also requires detailed information about the feasibility of implementation to help allocate scarce resources such as staff time, technology, and partnerships. Information about these program costs can be garnered from intervention intensity (eg, duration and extent of patient contacts) and intervention complexity (eg, range of needs addressed, intervention components).

To begin to inform implementation and scalability questions, we undertook this review of a scoping review of RCTs to better understand what the current literature reveals about the intensity and complexity of existing intervention models and the contribution of components of social needs interventions to health and health care utilization outcomes. Although numerous prior systematic and scoping reviews have synthesized the evidence on social care and social needs interventions,^18,19,20,21,22,23,24,25^ to our knowledge, this review of a scoping review is the first to focus on these crucial precursors to implementation and scalability. Specifically, we focus on (1) intensity and complexity of social needs interventions and (2) measurement of the effects of individual components or combinations of intervention components on behavioral, health, or health care utilization outcomes.

## Methods

### Data Sources and Searches

This review of a scoping review followed the Preferred Reporting Items for Systematic Reviews and Meta-Analyses (PRISMA) reporting guidelines.^26,27,28^ Our data source was a Patient-Centered Outcomes Research Institute–funded, web-based repository and visualization of social needs interventions in health care settings,^29^ which was built on systematic searches of articles in MEDLINE and the Cochrane Library published between January 1, 1995, and April 6, 2023; reference searches of relevant systematic reviews and companion articles; and consultation with subject matter experts (eTables 1-12 in Supplement 1). We registered the protocol for this review of a scoping review in the Open Science Framework.^30^

### Study Selection

eTable 13, the eMethods, and eFigure 1 in Supplement 1 detail the criteria used to select studies.^29^ The review selected English-language RCTs set in the US that addressed participant-level social needs^31,32^ and reported behavioral, health, or health care utilization outcomes or harms. A pair of investigators (M.V. and S.M.K., N.S. and M.L.E., or V.N. and S.K.) independently reviewed titles, abstracts, and full-text articles; disagreements were resolved by discussion or by a third reviewer (M.V. or M.L.E.).

### Data Extraction and Quality Assessment

A reviewer (S.M.K., N.S., V.N., or S.K.) extracted population and intervention characteristics, social needs addressed (eResults in Supplement 1), recruitment and intervention setting, and intervention practitioner; a second reviewer (M.V., M.L.E., or N.S.) checked for accuracy. A reviewer (M.V., N.S., or M.L.E.) assessed the risk of bias using the Risk of Bias 2.0 instrument,^33^ and a second reviewer (M.V., N.S., or M.L.E.) spot-checked the studies (eTable 14 in Supplement 1).

To understand intensity of social needs interventions, we extracted information on the number, duration, and frequency of contacts and the time period over which the contacts occurred.^34^ Given the underlying heterogeneity, we did not define thresholds a priori; instead, we employed an exploratory approach. Specifically, we selected the modal value to categorize the distribution as suggestive of lower vs higher intensity ( <8 contacts vs ≥8 contacts, mode = 8; less often than every 2 weeks vs 2 weeks or more often, mode = 2 weeks; <30 minutes vs ≥30 minutes, mode = 30 minutes; <6 months vs ≥6 months, mode = 6 months). Studies that planned to vary intensity based on participant needs were included in the high-intensity category (ie, varied by need) because they were designed to accommodate high intensity for at least some participants.

To capture features of complexity, we used the Complexity Assessment Tool for Systematic Reviews (iCAT-SR).^35^ Overall, we applied 5 of the 10 dimensions of the iCAT-SR tool to disaggregate complex interventions (eTable 15 and eTable 16 in Supplement 1). For complexity specifically, we included number of components (iCAT-SR dimension 1), behavior changes targeted in recipients (knowledge, action, or practice) (iCAT-SR dimension 2), and degree of tailoring intended or permitted (iCAT-SR dimension 4).^35^ Additionally, we assessed whether interventions addressed multiple social needs, had dedicated staff, involved multiple practitioners, and provided resources and/or active assistance with resources or required resources to implement (eg, information, economic supports, food, transportation, supplies, referral for participants, staff, training, time, space, or monetary resources).

We also applied the iCAT-SR framework to assess whether each study’s design permitted attribution of effects to 1 or more intervention components. We used 3 specific iCAT-SR criteria, either in combination with study design features or independently. First, we evaluated whether the studies could isolate the effect of the social needs intervention component. Studies comparing usual care plus a single-component social needs intervention with usual care alone permit causal inference on the effects of the single component; factorial trial designs may similarly permit causal inference regarding individual components. Prespecified or post hoc analyses of intervention components may not necessarily support causal inference because they may conflate selection and treatment effects, but they can offer an upper bound on the likely treatment effect.^36^ Multicomponent interventions (iCAT-SR dimension 1) and interventions addressing a combination of medical and social needs that have no prespecified or post hoc analyses of the effectiveness of intervention components address overall effectiveness but not the effectiveness of individual social needs components. Second, we judged whether context or setting (iCAT-SR dimension 8) or individual-level recipient or practitioner factors (iCAT-SR dimension 9) were likely to modify the effect of the intervention. Interventions that could be delivered under various settings with minimal modification were assessed as independent of context. Interventions likely to yield different results by setting or fully intertwined within a complex setting were moderately or highly dependent on context. Studies moderately or highly dependent on context and individual factors without additional analyses were judged as being unable to parse the effects of the intervention from the effects of context and individual factors.

### Data Analysis

We relied primarily on descriptive analyses. These analyses were supported by study counts and percentages (from proportions of all studies) and supplemented by qualitative syntheses of individual data elements.

## Results

We reviewed 15 114 references from database searches, 917 references from the Social Interventions Research and Evaluation Network, and 475 references from hand searches of systematic reviews, for a total of 16 506 references. We excluded 15 010 references at title and abstract review and assessed the full text of 1496 references. We excluded 1419 references at full-text review and included 77 RCTs^36,37,38,39,40,41,42,43,44,45,46,47,48,49,50,51,52,53,54,55,56,57,58,59,60,61,62,63,64,65,66,67,68,69,70,71,72,73,74,75,76,77,78,79,80,81,82,83,84,85,86,87,88,89,90,91,92,93,94,95,96,97,98,99,100,101,102,103,104,105,106,107,108,109,110,111,112^ reporting on 78 interventions in 93 publications with a total of 135 690 participants (eFigure 2 in Supplement 1).

Table 1 and eTable 17 in Supplement 1 present study and population characteristics. Of the 77 RCTs, 34 (44%) addressed both social and medical needs^38,40,41,46,47,48,49,50,51,52,54,56,57,58,59,60,65,66,69,70,73,74,75,76,77,82,84,90,92,94,97,101,104,108^ and 43 (56%) involved no medical care intervention component^36,37,39,42,43,44,45,53,55,61,62,63,64,67,68,71,72,78,79,80,81,83,85,86,87,88,89,91,93,95,96,98,99,100,102,103,105,106,107,109,110,111,112^; yet, all interventions were affiliated with or in health care settings, so participants may have received medical care indirectly. Nearly one-third addressed just 1 prespecified social need (25 RCTs [32%])^36,37,38,39,41,42,43,44,45,61,63,64,68,73,90,93,95,96,102,105,107,109,110,111,112^; the remainder (52 RCTs [68%]) addressed multiple social needs.^40,46,47,48,49,50,51,52,53,54,55,56,57,58,59,60,62,65,66,67,69,70,71,72,74,75,76,77,78,79,80,81,82,83,84,85,86,87,88,89,91,92,94,97,98,99,100,101,103,104,106,108^ The most frequently addressed social needs, alone or combined with other social needs, were health care access and quality (53 RCTs [69%]),^36,37,38,39,40,41,42,43,44,45,46,47,48,50,51,52,53,54,55,56,57,58,59,60,62,65,66,67,68,69,70,72,73,74,75,76,77,78,79,80,81,82,83,84,85,86,88,89,97,99,106,108,111^ housing stability and quality (35 RCTs [45%]),^40,46,47,48,49,50,51,53,54,55,56,59,62,65,69,76,77,78,79,80,81,82,83,85,86,87,88,90,91,92,93,94,95,103,106^ food security (30 RCTs [39%]),^48,50,53,56,58,59,61,62,63,64,65,70,72,74,76,77,78,81,82,85,86,87,91,92,96,98,101,103,104,109^ transportation (25 RCTs [32%]),^47,48,50,52,53,55,56,57,58,62,65,66,67,72,74,76,77,81,85,86,92,94,97,101,104^ and financial strain (23 RCTs [30%])^46,47,48,51,54,56,58,62,65,72,76,80,82,83,86,87,92,94,98,99,101,104,106^ (Figure). Nearly one-half of the studies (36 RCTs [47%]) also addressed additional unspecified social domains.^46,47,48,49,50,51,52,53,54,55,56,58,59,60,62,65,67,71,72,74,75,77,78,79,83,85,86,87,89,91,92,94,100,103,104,106^

**Table 1.** Study and Population Characteristics

Study characteristic	Randomized clinical trials, No. (%) (N =77)
Randomization	
Individually randomized parallel-group trial	72 (94)
Cluster-randomized parallel-group trial	5 (6)
Quality	
High	17 (22)
Medium	38 (49)
Low	22 (29)
Comparator	
Usual care	59 (77)
Active control	9 (12)
Waitlist control	4 (5)
Other inactive control	3 (4)
Other	2 (3)
Target domain type	
Social need program	43 (56)
Medical and social need program	34 (44)
No. of social need domains addressed^a^	
1 specified need	25 (32)
1 specified need and additional unspecified needs	4 (5)
2 specified needs	9 (12)
2 specified needs and additional unspecified needs	5 (6)
3 specified needs	3 (4)
3 specified needs and additional unspecified needs	5 (6)
4 specified needs	2 (3)
4 specified needs and additional unspecified needs	11 (14)
5 specified needs	0
5 specified needs and additional unspecified needs	8 (10)
≥ 6 specified needs	2 (3)
≥ 6 specified needs and additional unspecified needs	3 (4)
Age group^b^	
Children (<18 y) or children and their families	15 (19)
Adolescents and young adults (13-20 y)	9 (12)
Adults (≥18 y)	56 (73)
Older adults ( ≥50 y)	52 (68)
Only older adults (≥50 y)	6 (8)
Majority race or ethnicity^c^	
Majority Asian or Pacific Islander	0
Majority Black or Non-Hispanic Black	24 (36)
Majority Hispanic or Latino	12 (18)
Majority Native American, American Indian, or Indigenous	0
Majority White or Non-Hispanic White	16 (24)
No single group is a majority	15 (22)
Not reported	10 (NA)
Sex (proportion female)	
<50%	31 (42)
≥50%	42 (58)
Not reported	4 (NA)
Required clinical condition	
Mental health	11 (29)
Chronic condition(s)^d^	9 (24)
Diabetes	5 (13)
Asthma	3 (8)
Cardiovascular disease	3 (8)
Preterm birth	2 (5)
Blind or disabled	1 (3)
Heart failure or chronic obstructive pulmonary disease	1 (3)
Obesity	1 (3)
Interpersonal violence injury	1 (3)
Pregnancy	1 (3)
Selection for clinical condition, health care services, or both	
Clinical condition alone	27(35)
Clinical condition and use of health care services	11 (14)
Use of health care services	19 (25)
Not specific to clinical condition or use of health care services	20 (26)
Recruitment setting^b^	
Primary care	27 (35)
Hospital (inpatient)	17 (22)
Emergency department	16 (21)
Outpatient clinic	6 (8)
Recruited from health plan membership	5 (6)
Telephone-based care	4 (5)
Transitional housing	3 (4)
Urgent care	2 (3)
Web-based care	0 (0)
Home-based care	0 (0)
Other	21 (27)
Not reported	0
Intervention setting^b^	
Primary care	31 (44)
Home-based care	27 (38)
Telephone-based care	23 (32)
Hospital (inpatient)	8 (11)
Transitional housing	7 (10)
Outpatient clinic	6 (8)
Emergency department	4 (6)
Urgent care	2 (3)
Web-based care	2 (3)
Other	17 (24)
Not reported	6 (NA)

Abbreviation: NA, not applicable.

^a^ Studies may have reported addressing 1 or more of the prespecified social needs that were systematically captured. In addition, studies may have also reported that they addressed any need that arose in the population or social needs that were not prespecified.

^b^ Percentages add up to more than 100% because studies could have included participants in more than 1 age group, recruited from more than 1 setting, or have been conducted in more than 1 setting (eg, the group of studies with only older adults is also included in the group of studies with older adults).

^c^ Defined as more than 50%.

^d^ Studies included at least 1 specified or unspecified chronic condition.

**Figure.**
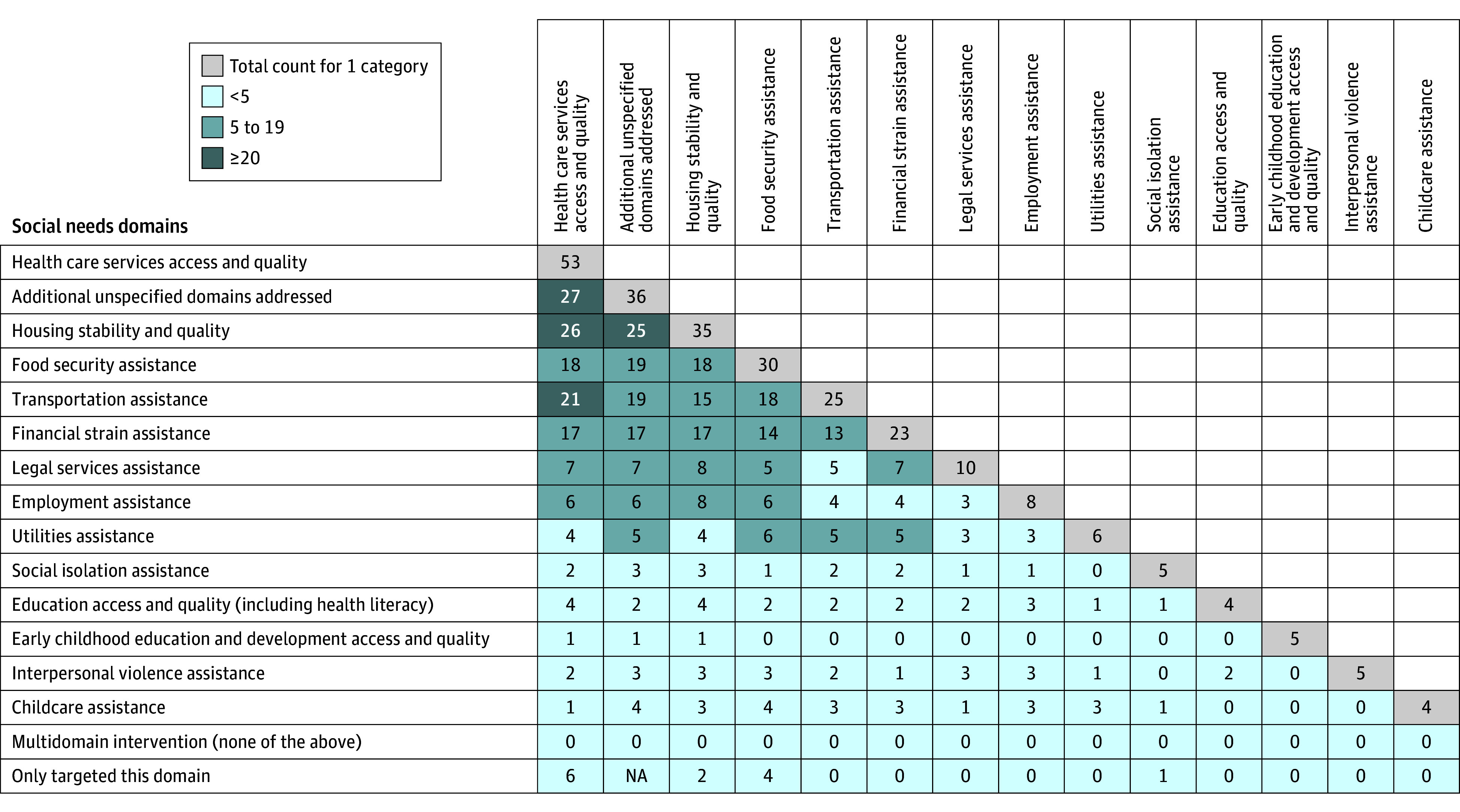
Domains Addressed by Social Needs Interventions Based on 77 trials. The numbers along the diagonal represent the total number of studies addressing the domain. Numbers along the last row represent the number of studies addressing a single domain. Numbers between the diagonal and the last row represent the number of studies addressing both the domain in the row and the domain in the column. NA indicates not applicable.

Most studies (56 RCTs [73%]) included adults aged 18 years and older.^36,37,38,39,42,44,45,46,48,50,51,52,53,55,56,57,58,59,60,61,62,63,64,66,68,69,72,73,74,75,76,77,78,79,80,81,82,83,84,85,88,89,90,91,92,93,94,95,96,97,101,103,104,106,108,109^ Among the studies reporting race and ethnicity, studies most commonly included majority Black and non-Hispanic Black populations (24 RCTs [36%]).^37,41,48,49,50,51,54,56,60,62,63,68,75,79,80,87,88,90,93,94,95,101,105,112^ Almost one-half of the 77 studies (38 RCTs [49%]) required a health condition for inclusion in the intervention.^37,40,43,46,49,50,54,55,56,57,58,59,61,63,64,67,68,73,75,79,80,82,83,88,89,91,92,93,94,96,97,99,101,105,107,108,109,112^

Studies reported multiple recruitment settings, most commonly referrals from agencies or shelters, primary care, outpatient or inpatient care, or emergency department settings. Interventions were most often conducted in primary care, home-based settings, or via telephone.

### Intensity and Complexity of Social Needs Interventions

eTable 18 in Supplement 1 characterizes the intensity and complexity of studies along multiple domains. Although reporting of planned intensity was inconsistent, 68 RCTs (88%) reported at least 1 feature suggestive of high intensity (≥8 contacts, ≥30 minutes per contact, frequency every 2 weeks or more often, duration ≥6 months, or home visits).^36,40,41,42,43,45,46,47,48,49,50,51,52,53,54,55,56,57,59,60,61,62,63,64,65,66,67,68,69,71,72,73,74,75,76,77,78,79,80,81,82,83,84,85,86,87,88,89,90,91,92,93,94,95,96,98,100,101,102,103,104,106,107,108,109,110,111,112^ Features of complexity (number of social needs addressed; whether or not a dedicated staff person mediated interactions between patients and the health care system; multiple practitioners, intervention components, behavior targets, resources offered to participants, or resources required to implement the program; and the ability to tailor the program) were more consistently reported than features of intensity. All studies reported at least 1 feature suggestive of complexity and 68 (88%) had 4 or more features that suggested complexity.^36,37,38,40,41,42,43,45,46,47,48,49,50,51,52,53,54,55,56,57,58,59,60,61,62,63,65,66,67,68,69,71,72,73,74,75,76,77,78,79,80,81,82,83,84,85,86,87,88,89,90,91,92,93,94,95,96,98,99,100,101,102,103,104,105,106,108,112^

### Intensity

The number of planned participant contacts (either in person or via telephone, text, or mail) ranged from 1^39,70,97^ to 25^56^ in the 35 studies (45%) that included these data.^39,41,42,43,45,46,47,49,50,53,56,57,58,59,60,61,63,64,68,70,71,73,75,79,81,84,91,94,97,98,105,107,109,110,112^ Intervention periods ranged from 2 weeks to 2 years. Planned contacts ranged from a single encounter to encounters at different intervals. Eight studies (10%) reported the planned duration of each contact, which ranged from 15 to 120 minutes.^42,45,59,60,75,94,104,107^ Twenty-six interventions (34%) included home visits, which presumably required a higher level of intensity to complete.^40,41,43,46,47,48,49,57,61,68,71,72,73,74,75,76,79,83,85,87,89,95,101,108,111,112^

Sixteen interventions (21%) reported on the actual number of contacts or their duration.^36,40,41,46,61,62,77,84,87,89,98,101,102,106,110,112^ The number of actual contacts (calls or text messages) ranged from 0 to 681. One intervention evaluating a care coordination intervention planned 24 contacts per participant, but the number of actual contacts ranged from 1 to 90.^41^ In 9 studies (12%) that reported on actual but not planned contacts, numbers ranged from no visits to a median of 79 care coordination activities or contacts per client.^36,40,62,77,87,89,101,102,106^

### Complexity

Regardless of whether studies were attempting to address social needs alone or both medical and social needs, they generally aimed to change multiple participant behaviors. Only a minority expected to affect a single participant behavior (22 RCTs [29%]) (Table 2).^38,39,42,44,45,55,59,61,63,64,66,68,70,82,86,92,95,97,105,106,109,111^

**Table 2.** Intervention Features

Key question	Randomized clinical trials, No. (%) (N = 77)
1: What is the intensity (eg, time, duration, or frequency) and complexity (eg, components and resources involved) of social needs interventions?	
Intensity	
Reported at least 1 feature indicating high intensity	
Yes	68 (88)
No	9 (12)
Reported any intensity feature	
Yes	73 (95)
No	4 (5)
Reported planned No. of contacts	
Yes	35 (45)
NR	42 (55)
Reported planned duration of contacts	
Yes	8 (10)
NR	69 (90)
Included home visits	
Yes	26 (34)
No	51 (66)
Reported data on actual No. of contacts or duration	
Yes	16 (21)
No	61 (79)
Complexity	
Reported any complexity feature	
Yes	77 (100)
No	0
Level of complexity	
≥4 Features suggesting complexity	68 (88)
≤3 Features suggesting complexity	9 (12)
Behaviors or actions the intervention intended to address (modified iCAT-SR dimension 2), No.	
≥3	40 (52)
2	2 (3)
1	22 (29)
Varies by patient need^a^	13 (17)
Reported staff mediating patient–health system interaction	
Yes	56 (73)
No	21 (27)
Multiple practitioners involved	
Yes	32 (43)
No	42 (57)
NR	3 (NA)
Practitioner^b^	
Health care practitioners (eg, doctors, nurses, or therapists)	32 (43)
Community health workers and navigators	31 (42)
Other nonprofessionals, including volunteers and study staff	22 (30)
Social worker	16 (22)
Case manager	14 (19)
Lawyers	2 (3)
NR	3 (NA)
Intervention components (modified iCAT-SR dimension 1), No.	
>1	67 (87)
1	10 (13)
Intervention component type^b^	
Active assistance with resources (vouchers, appointment scheduling, or enrollment form help)	62 (81)
Patient education (including on health, other social needs, or resources)	47 (61)
Passive referrals	28 (36)
Providing on-site resources	21 (27)
Screening	26 (34)
Health care practitioner education	5 (6)
Intervention recipient^b^	
Patient	66 (86)
Caregiver	15 (19)
Physician or other clinical staff	3 (4)
Community-based organizations	1 (1)
Did the intervention provide resources for participants?	
Referrals to resources, practitioners, or other supports	53 (69)
Information or educational materials, excluding referrals	41 (53)
Other	15 (19)
Supplies (eg, household items or monitoring devices)	11 (14)
Transportation assistance	11 (14)
Economic supports (eg, rent or utility assistance, nonfood vouchers, or money [excluding incentives for study participation])	8 (10)
Food (eg, food box or food voucher)	7 (9)
No, the intervention did not include provision of resources	3 (4)
Was tailoring, adaptation, or flexibility of the intervention intended? (modified iCAT-SR dimension 4)	
Yes, the intervention was tailored, could be adapted, and/or delivered flexibly	63 (82)
No, the intervention was delivered to all in the same way	10 (13)
Unclear or not stated	4 (5)
Among interventions with tailoring, what was the degree of tailoring? (iCAT-SR dimension 4) (n = 63)	
Highly tailored or very flexible	34 (54)
Moderately tailored or moderately flexible	23 (37)
Minimally tailored or slightly flexible	6 (10)
What was required to implement and/or deliver the intervention?	
Additional staff or nonstaff personnel (eg, community health workers, care coordinators, or peer mentors)	69 (90)
Referral sources	45 (58)
Time and/or space for visits or appointments (eg, added clinical visit, home visit, or telephone follow-up)	43 (56)
Training for staff or nonstaff personnel	38 (49)
Monetary or economic investment (not including existing available economic supports or incentives for participation)	11 (14)
Other	8 (10)
None	1 (1)
2: Can the effect of individual or combinations of intervention components on behavioral, health, or health care utilization outcomes be measured?	
How was the intervention or intervention components intended to be delivered? (iCAT dimension 1)	
>1 Component and some or all delivered as a bundle	44 (57)
>1 Component; may be integrated into a package	22 (29)
1 Component	11 (14)
Indicate the degree to which the effects of the intervention are dependent on the context or setting in which it is implemented. (iCAT-SR dimension 7)	
Highly dependent	15 (19)
Moderately dependent^c^	49 (64)
Independent^d^	13 (17)
Unclear or unable to assess	0
Indicate the degree to which the effects of the intervention are changed by individual-level factors (ie, recipient or practitioner factors) (iCAT-SR dimension 9)^e^	
Highly dependent on individual-level factors	51 (66)
Moderately dependent on individual-level factors	23 (30)
Independent of individual-level factors	3 (4)
Unclear or unable to assess	0

Abbreviations: iCAT-SR, Intervention Complexity Assessment Tool for Systematic Reviews; NA, not applicable; NR, not reported.

^a^ When the intervention was designed to address varying patient needs, the number of behaviors or actions intended to be addressed may have varied.

^b^ Percentages add up to more than 100% because practitioner, intervention components, and intervention recipients were not mutually exclusive (eg, more than 1 practitioner could have provided an intervention).

^c^ The effects of the intervention are likely to be transferrable across a limited range of settings only (eg, only within a specific country or health system).

^d^ The effects of the intervention do not appear to be highly dependent on the implementation setting (ie, it is anticipated that the effects of the intervention will be similar across a wide range of contexts or settings).

^e^ Highly, the effects of the intervention were modified by both recipient and practitioner factors; moderately, the effects of the intervention were modified by 1 or more recipient or practitioner factors; independent, the effects of the intervention are not modified substantially by recipient or practitioner factors.

The majority of studies reported including staff (case managers, care coordinators, patient navigators, community health workers, promotoras, or peer navigators) who mediated interactions between patients and health and/or social care systems (56 RCTs [73%]).^36,39,40,41,46,47,48,49,50,51,52,53,55,56,57,58,59,60,61,62,65,66,67,68,69,71,72,73,74,75,76,77,78,79,80,81,82,85,86,87,88,89,90,91,92,93,95,98,101,102,103,104,106,108,111,112^ Seventy-four studies specified the type of intervention practitioner (74 RCTS [96%])^36,37,38,39,40,41,42,43,44,45,46,47,48,49,50,51,52,53,54,55,56,57,58,59,60,61,62,63,64,65,66,67,68,69,70,71,72,73,74,75,76,77,78,79,80,81,82,83,84,85,86,87,88,89,90,91,92,93,94,95,96,98,99,100,101,102,103,104,105,106,108,110,111,112^; the majority of these 74 RCTs generally involved multiple practitioners (32 RCTs [43%]).^37,38,41,45,46,51,52,53,54,57,58,59,60,61,62,63,69,71,74,76,77,81,82,87,89,91,92,95,99,101,105,108^ Among the 74 studies reporting type of intervention practitioner, practitioners most frequently included health care practitioners (32 RCTs [43%]),^37,38,41,45,46,51,52,53,54,57,58,59,60,61,63,66,71,74,77,82,84,87,89,91,92,95,99,100,101,105,108,110^ community health workers (31 RCTs [42%]),^36,37,38,39,40,42,46,48,49,50,54,56,57,59,61,62,67,68,72,73,76,85,87,88,91,92,95,98,101,102,108^ and other non–health care professionals including volunteers and study staff (22 RCTs [30%]) (Table 2).^37,43,44,45,53,58,60,63,64,69,70,71,80,81,86,94,96,99,103,105,108,111^

Of the 77 RCTs, most included multiple components (67 RCTs [87%]).^36,37,38,40,41,42,43,44,45,46,47,48,49,50,51,52,53,54,55,56,57,58,59,60,61,62,63,64,65,66,67,68,69,71,72,73,74,75,76,77,79,80,82,83,84,85,86,87,88,89,90,91,92,93,94,95,98,99,100,101,102,104,105,106,107,108,112^ Most frequently, these included active assistance (such as scheduling appointments or filling out forms) (62 RCTs [81%])^38,39,40,41,42,43,44,45,46,47,48,49,50,51,52,53,54,56,57,58,59,62,63,65,66,67,68,69,70,71,72,73,74,75,76,77,78,79,80,82,83,84,85,86,87,89,90,91,92,93,95,96,97,98,99,101,104,106,108,109,111,112^ and patient education (47 RCTs [61%]).^37,40,41,42,43,45,46,47,49,50,52,54,55,56,57,58,59,60,61,64,71,73,74,75,78,80,81,82,84,85,86,89,92,94,95,96,99,101,102,103,105,106,107,108,110,111,112^ The component least commonly described was education of health care practitioners (5 RCTs [6%]).^37,41,57,58,108^

The vast majority of studies included provision of resources to participants (74 RCTs [96%]) as part of the intervention.^36,37,38,40,41,42,43,44,45,46,48,49,50,51,52,53,54,55,56,57,58,59,60,61,62,63,64,65,66,67,68,69,70,71,72,73,74,75,76,77,78,79,80,81,82,83,84,85,86,87,88,89,90,91,92,93,94,95,96,97,98,99,100,101,102,103,104,105,106,108,109,110,111,112^ These interventions typically described offering multiple resources (median [IQR] 2 [1-2] resources); only 28 studies listed a single resource being offered to participants.^36,42,50,51,56,67,68,70,78,79,80,82,83,84,87,89,91,92,93,94,97,100,103,106,108,109,111,112^ The resources provided to participants included referrals to practitioners, resource agencies, or other supports (53 RCTs [69%])^36,37,38,41,43,44,45,46,48,49,50,51,52,53,54,55,56,57,58,59,60,61,62,65,66,67,68,71,72,74,75,76,77,78,79,80,81,83,85,86,87,88,89,91,94,95,98,100,101,102,104,106,108^; information or educational materials (41 RCTs [53%])^37,40,41,42,44,46,48,49,52,55,57,58,59,60,61,63,64,66,70,71,72,73,74,75,77,81,84,85,86,88,96,98,99,101,102,103,104,105,110,111,112^; supplies, such as clothing or allergen impermeable bedding (11 RCTs [14%])^40,45,46,49,57,60,62,76,99,104,105^; and transportation (11 RCTs [14%]).^43,45,53,65,72,74,76,77,90,97,101^ Fewer studies provided economic supports, such as vouchers (8 RCTs [10%])^62,65,69,76,92,93,95,99^ or food resources (7 RCTs [9%]).^45,61,63,64,77,96,109^

For each listed resource, the complexity of effort in providing the resource and the degree of tailoring the resources varied substantially. Table 3 lists examples of resources that varied in level of tailoring. Most of the 63 studies^36,40,41,42,43,45,46,47,48,49,50,51,52,53,54,55,56,57,58,59,60,61,62,63,65,66,67,68,71,72,73,74,75,76,77,78,79,80,82,83,84,85,86,87,88,89,90,91,92,94,95,96,98,99,100,101,102,103,104,106,108,109,112^ that noted tailored offerings also listed community health workers, navigators, or other personnel as key intermediaries in providing the resource (46 RCTs [73%]).^36,41,47,48,49,50,51,52,53,55,56,57,58,59,60,61,62,65,66,67,68,71,72,73,74,75,76,77,79,80,82,85,86,87,88,89,90,91,92,95,98,101,102,103,104,106^ Studies frequently described additional staffing needs to conduct interventions (69 RCTs [90%]).^36,37,38,39,40,41,42,43,44,45,46,47,48,49,50,51,52,53,56,57,58,59,60,61,62,63,64,65,66,67,68,69,71,72,73,74,75,76,77,78,79,80,81,82,83,84,85,86,87,88,89,90,91,92,93,94,95,98,100,101,102,103,104,106,108,109,110,111,112^ Staffing included community health workers, practitioners, behavioral health specialists, care coordinators, social workers, peer navigators, and health educators, among others. Fewer studies explicitly described time or space requirements for interventions (43 RCTs [56%])^38,41,42,43,45,46,48,49,50,51,53,55,56,57,58,59,60,62,64,66,67,71,72,73,74,75,76,77,79,80,86,87,88,89,90,91,94,101,102,103,104,107,108^ or training needs for staff (38 RCTs [49%]).^36,40,42,46,48,49,50,53,55,56,57,58,60,64,68,71,73,76,79,81,84,85,86,87,88,89,90,91,94,95,98,102,103,104,105,110,111,112^ Training encompassed education on motivational interviewing, implicit bias, home assessment, care navigation, financial coaching, and specific conditions such as hypertension.

**Table 3.** Examples of Standardized Resources and Resources Tailored to Participant Needs

Resource type	Standardized resource example^a^	Tailored resource example
Information materials (excluding referrals)	20-min video about services^42^	Coaches identified parent strengths and navigated participants to cost-saving services and public benefits^104^
Economic supports	Subsidized housing^95^	Funds loaned to cover apartment security deposit^62^
Food	Biweekly fresh healthy foods^61^	Home-delivered food boxes tailored to nutritional needs and ethnic food preferences every 2 weeks, for 24 weeks^63^
Transportation assistance	Taxicab vouchers^97^	Navigators helped patients access medical transportation assistance through the state Medicaid system^53^
Supplies	Cell phones^76^	Program staff helped clients obtain donated furniture and appliances^62^
Referrals to resources or practitioners	Accompanied to clinic and introduced to care team^45^	Interventionists provided referrals to other health center resources if indicated^59^
Other	Free primary health care, radiology, and laboratory services^38^	Pro bono legal services provided to families with specific legal needs^54^

^a^ Standardized resources may have initially been developed via sociocultural tailoring.

### Measuring the Effects of Individual or Combinations of Intervention Components on Behavioral Outcomes, Health Outcomes, or Health Care Utilization Outcomes

All trials by design addressed overall effectiveness, but in most, the value of individual components could not be discerned by study design, analyses of components, or prior evidence. Regarding design, more than one-third of the interventions (27 RCTs [35%]) addressed both medical and social needs and compared them with usual care; these studies cannot speak to the effects of addressing social needs specifically.^41,46,47,48,50,51,52,54,56,57,58,59,60,65,69,70,73,74,76,77,82,84,90,92,101,104,108^ Only 4 studies (5%) compared usual care plus a single component addressing social needs with usual care alone; these studies directly address the causal effect of a single social needs intervention component.^81,103,110,111^ No study reported the use of multiphase optimization strategies.^113^

Regarding analyses of intervention components or characteristics, 13 studies (17%) planned or reported subanalyses (a priori or post hoc).^36,37,45,57,59,71,79,86,89,92,96,98,106^ Specifically, 1 study^57^ noted the infeasibility of randomizing all permutations of intervention components and described planned qualitative and quantitative approaches (implementation of components, mediator analyses, and perceptions of staff regarding effectiveness) to assess the effectiveness of components. One study^45^ reported on the results of a 2 × 2 factorial design. Eleven other studies reported on variations in outcomes by number of interactions, duration of interactions, or level of engagement or fidelity to planned interventions.^36,37,59,71,79,86,89,92,96,98,106^ The remaining majority (60 RCTs [78%]) were not designed or analyzed to address the effectiveness of intervention components. At the same time, the majority of interventions that did not plan or report subanalyses were moderately or highly dependent on context (51 RCTs [66%])^38,39,40,41,42,43,44,46,48,49,51,52,53,54,56,58,60,61,62,64,65,66,67,70,72,73,74,75,76,78,80,82,83,84,85,87,88,90,91,93,94,95,99,100,101,102,103,107,108,111,112^ and individual factors related to the patient or practitioner (61 RCTs [79%]).^38,39,40,41,42,43,44,46,47,48,49,50,51,52,53,54,55,56,58,60,61,62,63,64,65,66,67,69,72,73,74,75,76,77,78,80,81,82,83,84,85,87,88,90,91,93,94,95,97,99,100,101,102,103,105,107,108,109,110,111,112^ As a result, the effects of individual intervention components could not often be distinguished from the potential moderating effects of contextual factors. Finally, multicomponent studies rarely reported prior evidence (6 of 67 RCTs [9%]) to justify specific components (eTable 19 in Supplement 1),^49,71,91,99,104,107^ but often cited evidence for broad approaches, community needs, or prevalence of social needs as overall justification.

## Discussion

The findings of this review of a scoping review suggest that the social needs interventions described in published RCTs are often both highly complex and intensive. Although high levels of intensity and complexity may be essential to meaningfully address complex social issues, some evidence suggests higher intensity interventions do not universally result in better health outcomes.^114^ The majority of the intervention studies included in this review did not report assessing (by design; mediator, moderator, or other analyses; or citing prior studies) how different intervention components independently modify health or utilization outcomes, thereby heightening the challenge of implementing and scaling these programs.

The underlying complexity and interaction of social risk factors, context, setting, and outcomes limit the generalizability of RCTs testing single or even multiple intervention components in factorial designs.^114^ To address challenges of replication and scalability, several recent systematic reviews have called for new primary research that employs economic evaluations,^22^ larger controlled and rigorous studies,^18,25^ and better descriptions of clinical integration^19^ and implementation.^20^ Future research also may be strengthened by using multiphase optimization strategies or sequential multiple assignment randomized trial designs to help isolate effective components of these complex interventions.^113^ In addition, hybrid effectiveness-implementation designs that concurrently assess both intervention effectiveness and implementation outcomes could shed light on how implementation is associated with intervention effectiveness.^115^ Systematic reviews employing qualitative synthesis, meta-regression, finite mixture models, or qualitative comparative analysis can also help identify features of complex and intense interventions most associated with beneficial outcomes.^114^ Not every combination of components is appropriate or feasible for evaluation; selecting appropriate combinations for further evaluation will require qualitative analyses and judgment and use of designs not commonly employed. These approaches may be appropriate when effectiveness is established, the intervention has widespread applicability, intervention components are designed and intended to be separable, and understanding the effects of individual components is relevant to policy and practice. However, these types of studies also require larger sample sizes, more resources, and more time. All types of social needs intervention studies would benefit from better and more consistent reporting standards.

### Limitations

This study has limitations. First, information on social determinants of health is evolving; our broad and comprehensive search terms may have been insufficient to capture all relevant studies and their ancillary publications. Our searches were restricted to studies published in the health services literature. As a result, we likely missed studies indexed solely in the social sciences or economics literature. We also excluded nonrandomized studies that may have provided insights on selected combinations of intervention components. Second, intervention rationale and design were not always clearly reported. As a result, our subjective decisions may have led us to inadvertently exclude relevant studies; dual independent review may have mitigated this concern to some extent. Third, this review of a scoping review excluded comparative effectiveness trials of social needs interventions that may have undertaken more granular comparisons. Fourth, we relied on intensity metrics based on modal values. Future studies should establish thresholds that correspond to workforce and participant expectations. Fifth, studies varied substantially in whether and how intensity and complexity were reported; as a result, patterns observed may at least in part reflect variations in reporting rather than intervention design. Sixth, we did not intend to synthesize and grade the evidence on specific outcomes; future syntheses should couple evaluations of intensity and complexity with intervention effectiveness.

## Conclusions

Social needs interventions are typically complex, intense, and include multiple components. By design, RCTs of these interventions address overall effectiveness but are rarely designed to distinguish the causal effects of specific components, despite being resource intensive. Future studies with hybrid effectiveness-implementation or sequential designs and more standardized reporting of intervention intensity and complexity could help stakeholders assess the return on investment of these interventions.

## Notes

Supplement 1. **eTable 1.** Ovid MEDLINE® Search String and Yield for Access to Care MEDLINE Search (Ovid MEDLINE®) (April 6, 2023) **eTable 2.** Cochrane Library (Including Both Cochrane Database of Systematic Reviews and Cochrane Central Register of Controlled Trials) Search String and Yield for Access to Care (April 6, 2023) **eTable 3.** Ovid MEDLINE® Search String and Yield for Food Insecurity, Housing, Education and Literacy, Financial Strain, Employment, Transportation, Utilities, Social Isolation, Early Childhood Development, Legal Services, and Childcare (Ovid MEDLINE®) (April 6, 2023) **eTable 4.** Cochrane Library (Including Both Cochrane Database of Systematic Reviews and Cochrane Central Register of Controlled Trials) Search String and Yield for Food Insecurity, Housing, Education and Literacy, Financial Strain, Employment, Transportation, Utilities, Social Isolation, Early Childhood Development, Legal Services, and Childcare (April 6, 2023) **eTable 5.** Ovid MEDLINE® Search String and Yield for Interpersonal Violence MEDLINE Search (Ovid MEDLINE®) (April 6, 2023) **eTable 6.** Cochrane Library (Including Both Cochrane Database of Systematic Reviews and Cochrane Central Register of Controlled Trials) Search String and Yield for Interpersonal Violence (April 6, 2023) **eTable 7.** Ovid MEDLINE® Search String and Yield for Access to Care MEDLINE Search (Ovid MEDLINE®) (February 7, 2023) **eTable 8.** Cochrane Library (Including Both Cochrane Database of Systematic Reviews and Cochrane Central Register of Controlled Trials) Search String and Yield for Access to Care (February 1, 2023) **eTable 9.** Ovid MEDLINE® Search String and Yield for Food Insecurity, Housing, Education and Literacy, Financial Strain, Employment, Transportation, Utilities, Social Isolation, Early Childhood Development, Legal Services, and Childcare (Ovid MEDLINE®) (February 7, 2023) **eTable 10.** Cochrane Library (Including Both Cochrane Database of Systematic Reviews and Cochrane Central Register of Controlled Trials) Search String and Yield for Food Insecurity, Housing, Education and Literacy, Financial Strain, Employment, Transportation, Utilities, Social Isolation, Early Childhood Development, Legal Services, and Childcare (February 1, 2023) **eTable 11.** Ovid MEDLINE® Search String and Yield for Interpersonal Violence MEDLINE Search (Ovid MEDLINE®) (February 7, 2023) **eTable 12.** Cochrane Library (Including Both Cochrane Database of Systematic Reviews and Cochrane Central Register of Controlled Trials) Search String and Yield for Interpersonal Violence (February 1, 2023) **eTable 13.** Inclusion and Exclusion Criteria **eMethods.** Identification of Social Needs **eFigure 1.** Screening Approach **eResults.** Identification of Social Needs **eTable 14.** Risk of Bias Domains and Ratings **eTable 15.** iCAT SR Dimensions, Assessment Categories, and Elaboration and Explanations **eTable 16.** RCT Abstraction Form Items Adapted From iCAT **eFigure 2.** Article Flow **eTable 17.** Description of how Social Needs Were Identified **eTable 18.** Measures of Intervention Intensity and Complexity **eTable 19.** Justification of Intervention Components
eReferences.

Supplement 2.
Data Sharing Statement
