# Supplementary material for: Evaluating Intensity, Complexity, and Potential for Causal Inference in Social Needs Interventions: A Review of a Scoping Review
Source: JAMA Netw Open. 2024 Jun 21;7(6):e2417994. doi: 10.1001/jamanetworkopen.2024.17994 (PMC11193129; doi:10.1001/jamanetworkopen.2024.17994)
Supplement: Supplement 1. — eTable 1. Ovid MEDLINE® Search String and Yield for Access to Care MEDLINE Search (Ovid MEDLINE®) (April 6, 2023) eTable 2. Cochrane Library (Including Both Cochrane Database of Systematic Reviews and Cochrane Central Register of Controlled Trials) Search String and Yield for Access to Care (April 6, 2023) eTable 3. Ovid MEDLINE® Search String and Yield for Food Insecurity, Housing, Education and Literacy, Financial Strain, Employment, Transportation, Utilities, Social Isolation, Early Childhood Development, Legal Services, and Childcare (Ovid MEDLINE®) (April 6, 2023) eTable 4. Cochrane Library (Including Both Cochrane Database of Systematic Reviews and Cochrane Central Register of Controlled Trials) Search String and Yield for Food Insecurity, Housing, Education and Literacy, Financial Strain, Employment, Transportation, Utilities, Social Isolation, Early Childhood Development, Legal Services, and Childcare (April 6, 2023) eTable 5. Ovid MEDLINE® Search String and Yield for Interpersonal Violence MEDLINE Search (Ovid MEDLINE®) (April 6, 2023) eTable 6. Cochrane Library (Including Both Cochrane Database of Systematic Reviews and Cochrane Central Register of Controlled Trials) Search String and Yield for Interpersonal Violence (April 6, 2023) eTable 7. Ovid MEDLINE® Search String and Yield for Access to Care MEDLINE Search (Ovid MEDLINE®) (February 7, 2023) eTable 8. Cochrane Library (Including Both Cochrane Database of Systematic Reviews and Cochrane Central Register of Controlled Trials) Search String and Yield for Access to Care (February 1, 2023) eTable 9. Ovid MEDLINE® Search String and Yield for Food Insecurity, Housing, Education and Literacy, Financial Strain, Employment, Transportation, Utilities, Social Isolation, Early Childhood Development, Legal Services, and Childcare (Ovid MEDLINE®) (February 7, 2023) eTable 10. Cochrane Library (Including Both Cochrane Database of Systematic Reviews and Cochrane Central Register of Controlled Trials) Search String an [file jamanetwopen-e2417994-s001.pdf]

Supplemental Online Content

Viswanathan M, Kennedy SM, Sathe N, et al. Evaluating intensity, complexity, and potential for causal inference in social needs interventions. *JAMA Netw Open*. 2024;7(6):e2417994. doi:10.1001/jamanetworkopen.2024.17994

eTable 1. Ovid MEDLINE® Search String and Yield for Access to Care MEDLINE Search (Ovid MEDLINE®) (April 6, 2023)

eTable 2. Cochrane Library (Including Both Cochrane Database of Systematic Reviews and Cochrane Central Register of Controlled Trials) Search String and Yield for Access to Care (April 6, 2023)

eTable 3. Ovid MEDLINE® Search String and Yield for Food Insecurity, Housing, Education and Literacy, Financial Strain, Employment, Transportation, Utilities, Social Isolation, Early Childhood Development, Legal Services, and Childcare (Ovid MEDLINE®) (April 6, 2023)

eTable 4. Cochrane Library (Including Both Cochrane Database of Systematic Reviews and Cochrane Central Register of Controlled Trials) Search String and Yield for Food Insecurity, Housing, Education and Literacy, Financial Strain, Employment, Transportation, Utilities, Social Isolation, Early Childhood Development, Legal Services, and Childcare (April 6, 2023)

eTable 5. Ovid MEDLINE® Search String and Yield for Interpersonal Violence MEDLINE Search (Ovid MEDLINE®) (April 6, 2023)

eTable 6. Cochrane Library (Including Both Cochrane Database of Systematic Reviews and Cochrane Central Register of Controlled Trials) Search String and Yield for Interpersonal Violence (April 6, 2023)

eTable 7. Ovid MEDLINE® Search String and Yield for Access to Care MEDLINE Search (Ovid MEDLINE®) (February 7, 2023)

eTable 8. Cochrane Library (Including Both Cochrane Database of Systematic Reviews and Cochrane Central Register of Controlled Trials) Search String and Yield for Access to Care (February 1, 2023)

eTable 9. Ovid MEDLINE® Search String and Yield for Food Insecurity, Housing, Education and Literacy, Financial Strain, Employment, Transportation, Utilities, Social Isolation, Early Childhood Development, Legal Services, and Childcare (Ovid MEDLINE®) (February 7, 2023)

eTable 10. Cochrane Library (Including Both Cochrane Database of Systematic Reviews and Cochrane Central Register of Controlled Trials) Search String and Yield for Food Insecurity, Housing, Education and Literacy, Financial Strain, Employment,

© 2024 Viswanathan M et al. *JAMA Network Open.*

Transportation, Utilities, Social Isolation, Early Childhood Development, Legal Services, and Childcare (February 1, 2023)

eTable 11. Ovid MEDLINE® Search String and Yield for Interpersonal Violence MEDLINE Search (Ovid MEDLINE®) (February 7, 2023)

eTable 12. Cochrane Library (Including Both Cochrane Database of Systematic Reviews and Cochrane Central Register of Controlled Trials) Search String and Yield for Interpersonal Violence (February 1, 2023)

eTable 13. Inclusion and Exclusion Criteria

eMethods. Identification of Social Needs

eFigure1. Screening Approach

eResults. Identification of Social Needs

eTable 14. Risk of Bias Domains and Ratings

eTable 15. iCAT SR Dimensions, Assessment Categories, and Elaboration and Explanations

eTable 16. RCT Abstraction Form Items Adapted from iCAT

eFigure 2. Article Flow

eTable 17. Description of how Social Needs Were Identified

eTable 18. Measures of Intervention Intensity and Complexity

eTable 19. Justification of Intervention Components

eReferences

This supplemental material has been provided by the authors to give readers additional information about their work.

This supplement includes tables from the February 2023 and April 2023 search updates. To access prior search information, see the reports at https://www.pcori.org/impact/evidence-maps-and-visualizations/social-needs-interventions-improve-health-outcomes.

eTable 1. Ovid MEDLINE® Search String and Yield for Access to Care MEDLINE Search (Ovid MEDLINE®) (April 6, 2023)

Search	Query	Items Found
1	Social Determinants of Health.mp. [mp=title, book title, abstract, original title, name of substance word, subject heading word, floating sub-heading word, keyword heading word, organism supplementary concept word, protocol supplementary concept word, rare disease supplementary concept word, unique identifier, synonyms, population supplementary concept word, anatomy supplementary concept word]	11953
2	Social Conditions.mp. [mp=title, book title, abstract, original title, name of substance word, subject heading word, floating sub-heading word, keyword heading word, organism supplementary concept word, protocol supplementary concept word, rare disease supplementary concept word, unique identifier, synonyms, population supplementary concept word, anatomy supplementary concept word]	12017
3	Social Environment.mp. [mp=title, book title, abstract, original title, name of substance word, subject heading word, floating sub-heading word, keyword heading word, organism supplementary concept word, protocol supplementary concept word, rare disease supplementary concept word, unique identifier, synonyms, population supplementary concept word, anatomy supplementary concept word]	49316
4	Social Class.mp. [mp=title, book title, abstract, original title, name of substance word, subject heading word, floating sub-heading word, keyword heading word, organism supplementary concept word, protocol supplementary concept word, rare disease supplementary concept word, unique identifier, synonyms, population supplementary concept word, anatomy supplementary concept word]	49319
5	Socioeconomic Factors.mp. [mp=title, book title, abstract, original title, name of substance word, subject heading word, floating sub-heading word, keyword heading word, organism supplementary concept word, protocol supplementary concept word, rare disease supplementary concept word, unique identifier, synonyms, population supplementary concept word, anatomy supplementary concept word]	177732
6	(social* adj1 determin*).ti,ab,kf.	13304
7	((determinant* or determinate*) adj2 health).ti,ab,kf.	14781
8	((social* or socio*) adj1 condition*).ti,ab,kf.	7803
9	((social* or socio*) adj1 environment*).ti,ab,kf.	13486
10	((social* or socio*) adj1 (factor* or gradient*)).ti,ab,kf.	45737
11	((social* or socio*) adj1 (need* or require*)).ti,ab,kf.	3223
12	((social* or socio*) adj1 (equit* or inequit* or disparit* or equal* or inequal*)).ti,ab,kf.	11327
13	((social* or socio*) adj1 (hardship* or depriv* or challeng* or difficult* or barrier* or vulnerab* or disadvantag*)).ti,ab,kf.	16764
14	((social* or socio*) adj1 risk*).ti,ab,kf.	3448
15	((social* or socio*) adj1 (status* or circumstance* or position* or class* or standing)).ti,ab,kf.	72046
16	1 or 2 or 3 or 4 or 5 or 6 or 7 or 8 or 9 or 10 or 11 or 12 or 13 or 14 or 15	349839
17	Early Intervention, Educational.mp. [mp=title, book title, abstract, original title, name of substance word, subject heading word, floating sub-heading word, keyword heading word, organism supplementary concept word, protocol supplementary concept word, rare disease supplementary concept word, unique identifier, synonyms, population supplementary concept word, anatomy supplementary concept word]	3494
18	Internet-Based Intervention.mp. [mp=title, book title, abstract, original title, name of substance word, subject heading word, floating sub-heading word, keyword heading word, organism supplementary concept word, protocol supplementary concept word, rare disease supplementary concept word, unique identifier, synonyms, population supplementary concept word, anatomy supplementary concept word]	1485
19	Early Medical Intervention.mp. [mp=title, book title, abstract, original title, name of substance word, subject heading word, floating sub-heading word, keyword heading word, organism supplementary concept word, protocol supplementary concept word,	3641

Search	Query	Items Found
	rare disease supplementary concept word, unique identifier, synonyms, population supplementary concept word, anatomy supplementary concept word]	
20	Needs Assessment.mp. [mp=title, book title, abstract, original title, name of substance word, subject heading word, floating sub-heading word, keyword heading word, organism supplementary concept word, protocol supplementary concept word, rare disease supplementary concept word, unique identifier, synonyms, population supplementary concept word, anatomy supplementary concept word]	36288
21	Program Development.mp. [mp=title, book title, abstract, original title, name of substance word, subject heading word, floating sub-heading word, keyword heading word, organism supplementary concept word, protocol supplementary concept word, rare disease supplementary concept word, unique identifier, synonyms, population supplementary concept word, anatomy supplementary concept word]	32353
22	(Referral and Consultation).mp. [mp=title, book title, abstract, original title, name of substance word, subject heading word, floating sub-heading word, keyword heading word, organism supplementary concept word, protocol supplementary concept word, rare disease supplementary concept word, unique identifier, synonyms, population supplementary concept word, anatomy supplementary concept word]	77783
23	Pilot Projects.mp. [mp=title, book title, abstract, original title, name of substance word, subject heading word, floating sub-heading word, keyword heading word, organism supplementary concept word, protocol supplementary concept word, rare disease supplementary concept word, unique identifier, synonyms, population supplementary concept word, anatomy supplementary concept word]	146750
24	Social Welfare.mp. [mp=title, book title, abstract, original title, name of substance word, subject heading word, floating sub-heading word, keyword heading word, organism supplementary concept word, protocol supplementary concept word, rare disease supplementary concept word, unique identifier, synonyms, population supplementary concept word, anatomy supplementary concept word]	12048
25	Patient Navigation.mp. [mp=title, book title, abstract, original title, name of substance word, subject heading word, floating sub-heading word, keyword heading word, organism supplementary concept word, protocol supplementary concept word, rare disease supplementary concept word, unique identifier, synonyms, population supplementary concept word, anatomy supplementary concept word]	1469
26	Patient Advocacy.mp. [mp=title, book title, abstract, original title, name of substance word, subject heading word, floating sub-heading word, keyword heading word, organism supplementary concept word, protocol supplementary concept word, rare disease supplementary concept word, unique identifier, synonyms, population supplementary concept word, anatomy supplementary concept word]	25212
27	Inservice Training.mp. [mp=title, book title, abstract, original title, name of substance word, subject heading word, floating sub-heading word, keyword heading word, organism supplementary concept word, protocol supplementary concept word, rare disease supplementary concept word, unique identifier, synonyms, population supplementary concept word, anatomy supplementary concept word]	20870
28	Staff Development.mp. [mp=title, book title, abstract, original title, name of substance word, subject heading word, floating sub-heading word, keyword heading word, organism supplementary concept word, protocol supplementary concept word, rare disease supplementary concept word, unique identifier, synonyms, population supplementary concept word, anatomy supplementary concept word]	10960
29	intervention*.ti,ab,kf.	1103956
30	(need* adj2 (assessment* or evaluat* or determin*)).ti,ab,kf.	68264
31	patient navigat*.ti,ab,kf.	1313
32	patient advoca*.ti,ab,kf.	2852
33	((staff or employee*) adj2 (develop* or train* or educat* or curricul*)).ti,ab,kf.	16752
34	((social* or socio* or communit* or neighbor* or neighbour*) adj3 (refer* or partner*)).ti,ab,kf.	14223
35	17 or 18 or 19 or 20 or 21 or 22 or 23 or 24 or 25 or 26 or 27 or 28 or 29 or 30 or 31 or 32 or 33 or 34	1476158
36	Health Services Accessibility.mp. [mp=title, book title, abstract, original title, name of substance word, subject heading word, floating sub-heading word, keyword heading word, organism supplementary concept word, protocol supplementary concept word,	85360

© 2024 Viswanathan M et al. *JAMA Network Open.*

Search	Query	Items Found
	rare disease supplementary concept word, unique identifier, synonyms, population supplementary concept word, anatomy supplementary concept word]	
37	Health Equity.mp. [mp=title, book title, abstract, original title, name of substance word, subject heading word, floating sub-heading word, keyword heading word, organism supplementary concept word, protocol supplementary concept word, rare disease supplementary concept word, unique identifier, synonyms, population supplementary concept word, anatomy supplementary concept word]	7911
38	Right to Health.mp. [mp=title, book title, abstract, original title, name of substance word, subject heading word, floating sub-heading word, keyword heading word, organism supplementary concept word, protocol supplementary concept word, rare disease supplementary concept word, unique identifier, synonyms, population supplementary concept word, anatomy supplementary concept word]	1677
39	Universal Health Care.mp. [mp=title, book title, abstract, original title, name of substance word, subject heading word, floating sub-heading word, keyword heading word, organism supplementary concept word, protocol supplementary concept word, rare disease supplementary concept word, unique identifier, synonyms, population supplementary concept word, anatomy supplementary concept word]	1362
40	(primary care adj3 (access* or avail* or utiliz*)).ti,ab,kf.	3686
41	(health services adj3 (access* or avail* or utiliz*)).ti,ab,kf.	10096
42	(healthcare adj3 (access* or avail* or utiliz*)).ti,ab,kf.	19601
43	(health care adj3 (access* or avail* or utiliz*)).ti,ab,kf.	27984
44	36 or 37 or 38 or 39 or 40 or 41 or 42 or 43	137488
45	Primary Health Care.mp. [mp=title, book title, abstract, original title, name of substance word, subject heading word, floating sub-heading word, keyword heading word, organism supplementary concept word, protocol supplementary concept word, rare disease supplementary concept word, unique identifier, synonyms, population supplementary concept word, anatomy supplementary concept word]	104126
46	Comprehensive Health Care.mp. [mp=title, book title, abstract, original title, name of substance word, subject heading word, floating sub-heading word, keyword heading word, organism supplementary concept word, protocol supplementary concept word, rare disease supplementary concept word, unique identifier, synonyms, population supplementary concept word, anatomy supplementary concept word]	7459
47	General Practice.mp. [mp=title, book title, abstract, original title, name of substance word, subject heading word, floating sub-heading word, keyword heading word, organism supplementary concept word, protocol supplementary concept word, rare disease supplementary concept word, unique identifier, synonyms, population supplementary concept word, anatomy supplementary concept word]	49453
48	General Practitioners.mp. [mp=title, book title, abstract, original title, name of substance word, subject heading word, floating sub-heading word, keyword heading word, organism supplementary concept word, protocol supplementary concept word, rare disease supplementary concept word, unique identifier, synonyms, population supplementary concept word, anatomy supplementary concept word]	40736
49	Family Practice/	66938
50	Physicians, Family/	17169
51	Physicians, Primary Care/	4317
52	Primary Care Nursing/	565
53	Nurse Practitioners/	18795
54	Family Nurse Practitioners/	75
55	Pediatric Nurse Practitioners/	187
56	Physician Assistants/	6297
57	Family Nursing/	1564
58	Community Health Nursing/	19761
59	Community Health Centers/	7511
60	Community Mental Health Centers/	3032
61	Community Health Services/	33035
62	Community Mental Health Services/	19022
63	Community Health Workers/	6535
64	Safety-net Providers/	1317
65	primary care.ti,ab,kf.	125354
66	primary health care.ti,ab,kf.	29023

© 2024 Viswanathan M et al. *JAMA Network Open.*

Search	Query	Items Found
67	((family or general or primary) adj1 (medicine or practice or practitioner* or physician* or doctor* or provider* or clinic* or clinician*)).ti,ab,kf.	130334
68	36 or 37 or 38 or 39 or 40 or 41 or 42 or 43 or 44 or 45 or 46 or 47 or 48 or 49 or 50 or 51 or 52 or 53 or 54 or 55 or 56 or 57 or 58 or 59 or 60 or 61 or 62 or 63 or 64 or 65 or 66 or 67	519625
69	16 and 35 and 68	10318
70	limit 69 to (yr="1995 -Current" and (systematic reviews pre 2019 or systematic reviews))	584
71	(systematic adj3 (review or assess* or eval*)).ti.	177714
72	69 and 71	227
73	70 or 72	586
74	35 and 44	30705
75	limit 74 to (yr="1995 -Current" and (systematic reviews pre 2019 or systematic reviews))	2252
76	71 and 74	900
77	75 or 76	2257
78	73 or 77	2475
79	limit 78 to last year	318

eTable 2. Cochrane Library (Including Both Cochrane Database of Systematic Reviews and Cochrane Central Register of Controlled Trials) Search String and Yield for Access to Care (April 6, 2023)

ID	Search	Hits
#1	social*:ti,ab,kw near/1 determin*:ti,ab,kw	611
#2	(determinant* or determinate*):ti,ab,kw near/2 health:ti,ab,kw	649
#3	(social* or socio*):ti,ab,kw near/1 condition*:ti,ab,kw	480
#4	(social* or socio*):ti,ab,kw near/1 environment*:ti,ab,kw	1845
#5	(social* or socio*):ti,ab,kw near/1 (factor* or gradient*):ti,ab,kw	6270
#6	(social* or socio*):ti,ab,kw near/1 (need* or require*):ti,ab,kw	326
#7	(social* or socio*):ti,ab,kw near/1 (equit* or inequit* or disparit* or equal* or inequal*):ti,ab,kw	268
#8	(social* or socio*):ti,ab,kw near/1 (hardship* or depriv* or challeng* or difficult* or barrier* or vulnerab* or disadvantag*):ti,ab,kw	1478
#9	(social* or socio*):ti,ab,kw near/1 risk*:ti,ab,kw	302
#10	(social* or socio*):ti,ab,kw near/1 (status* or circumstance* or position* or class* or standing):ti,ab,kw	6356
#11	#1 OR #2 OR #3 OR #4 OR #5 OR #6 OR #7 OR #8 OR #9 OR #10	16202
#12	intervention*:ti,ab,kw	534075
#13	need*:ti,ab,kw near/2 (assessment* or evaluat* or determin*):ti,ab,kw	10318
#14	program*:ti,ab,kw near/2 develop*:ti,ab,kw	4926
#15	pilot:ti,ab,kw next project*:ti,ab,kw	26244
#16	patient*:ti,ab,kw near/1 navigat*:ti,ab,kw	759
#17	patient*:ti,ab,kw near/2 advoca*:ti,ab,kw	447
#18	(staff or employee*):ti,ab,kw near/2 (develop* or train* or educat* or curricul*):ti,ab,kw	3157
#19	(social* or socio* or communit* or neighbor* or neighbour*):ti,ab,kw near/3 (refer* or partner*):ti,ab,kw	1895
#20	#12 OR #13 OR #14 OR #15 OR #16 OR #17 OR #18 OR #19	559953
#21	"primary care":ti,ab,kw near/3 (access* or avail* or utiliz*or utilis*):ti,ab,kw	334
#22	"health services":ti,ab,kw near/3 (access* or avail* or utiliz*or utilis*):ti,ab,kw	1533
#23	healthcare:ti,ab,kw near/3 (access* or avail* or utiliz*or utilis*):ti,ab,kw	1171
#24	"health care":ti,ab,kw near/3 (access* or avail* or utiliz*or utilis*):ti,ab,kw	1818
#25	#21 OR #22 OR #23 OR #24	4452
#26	primary:ti,ab,kw next care:ti,ab,kw	21368
#27	comprehensive:ti,ab,kw next care:ti,ab,kw	373
#28	"primary health care":ti,ab,kw	8252
#29	"comprehensive health care":ti,ab,kw	113

© 2024 Viswanathan M et al. *JAMA Network Open.*

ID	Search	Hits
#30	comprehensive:ti,ab,kw next healthcare:ti,ab,kw	19
#31	primary:ti,ab,kw next healthcare:ti,ab,kw	882
#32	(safety-net:ti,ab,kw or "safety net":ti,ab,kw) next clinic*:ti,ab,kw	94
#33	"community health center":ti,ab,kw	344
#34	"community health centers":ti,ab,kw	764
#35	"federally qualified health center":ti,ab,kw	176
#36	"federally qualified health centers":ti,ab,kw	164
#37	fqhc:ti,ab,kw	109
#38	(family or general or primary):ti,ab,kw near/2 (medicine or practice or practitioner* or physician* or doctor* or provider* or clinic* or clinician* or nurs*):ti,ab,kw	32580
#39	#26 OR #27 OR #28 OR #29 OR #30 OR #31 OR #32 OR #33 OR #34 OR #35 OR #36 OR #37 OR #38	46379
#40	#25 OR #39	49590
#41	#11 AND #20 AND #40	1419
#42	#20 AND #25	2963
#43	#41 OR #42 with Cochrane Library publication date in The last year	429

eTable 3. Ovid MEDLINE® Search String and Yield for Food Insecurity, Housing, Education and Literacy, Financial Strain, Employment, Transportation, Utilities, Social Isolation, Early Childhood Development, Legal Services, and Childcare (Ovid MEDLINE®) (April 6, 2023)

Search	Query	Items Found
1	Social Determinants of Health.mp. [mp=title, book title, abstract, original title, name of substance word, subject heading word, floating sub-heading word, keyword heading word, organism supplementary concept word, protocol supplementary concept word, rare disease supplementary concept word, unique identifier, synonyms, population supplementary concept word, anatomy supplementary concept word]	11953
2	Social Conditions.mp. [mp=title, book title, abstract, original title, name of substance word, subject heading word, floating sub-heading word, keyword heading word, organism supplementary concept word, protocol supplementary concept word, rare disease supplementary concept word, unique identifier, synonyms, population supplementary concept word, anatomy supplementary concept word]	12017
3	Social Environment.mp. [mp=title, book title, abstract, original title, name of substance word, subject heading word, floating sub-heading word, keyword heading word, organism supplementary concept word, protocol supplementary concept word, rare disease supplementary concept word, unique identifier, synonyms, population supplementary concept word, anatomy supplementary concept word]	49316
4	Social Class.mp. [mp=title, book title, abstract, original title, name of substance word, subject heading word, floating sub-heading word, keyword heading word, organism supplementary concept word, protocol supplementary concept word, rare disease supplementary concept word, unique identifier, synonyms, population supplementary concept word, anatomy supplementary concept word]	49319
5	Socioeconomic Factors.mp. [mp=title, book title, abstract, original title, name of substance word, subject heading word, floating sub-heading word, keyword heading word, organism supplementary concept word, protocol supplementary concept word, rare disease supplementary concept word, unique identifier, synonyms, population supplementary concept word, anatomy supplementary concept word]	177732
6	(social* adj1 determin*).ti,ab,kf.	13304
7	((determinant* or determinate*) adj2 health).ti,ab,kf.	14781
8	((social* or socio*) adj1 condition*).ti,ab,kf.	7803
9	((social* or socio*) adj1 environment*).ti,ab,kf.	13486
10	((social* or socio*) adj1 (factor* or gradient*)).ti,ab,kf.	45737
11	((social* or socio*) adj1 (need* or require*)).ti,ab,kf.	3223
12	((social* or socio*) adj1 (equit* or inequit* or disparit* or equal* or inequal*)).ti,ab,kf.	11327
13	((social* or socio*) adj1 (hardship* or depriv* or challeng* or difficult* or barrier* or vulnerab* or disadvantag*)).ti,ab,kf.	16764
14	((social* or socio*) adj1 risk*).ti,ab,kf.	3448

© 2024 Viswanathan M et al. *JAMA Network Open.*

Search	Query	Items Found
15	((social* or socio*) adj1 (status* or circumstance* or position* or class* or standing)).ti,ab,kf.	72046
16	1 or 2 or 3 or 4 or 5 or 6 or 7 or 8 or 9 or 10 or 11 or 12 or 13 or 14 or 15	349839
17	Early Intervention, Educational.mp. [mp=title, book title, abstract, original title, name of substance word, subject heading word, floating sub-heading word, keyword heading word, organism supplementary concept word, protocol supplementary concept word, rare disease supplementary concept word, unique identifier, synonyms, population supplementary concept word, anatomy supplementary concept word]	3494
18	Internet-Based Intervention.mp. [mp=title, book title, abstract, original title, name of substance word, subject heading word, floating sub-heading word, keyword heading word, organism supplementary concept word, protocol supplementary concept word, rare disease supplementary concept word, unique identifier, synonyms, population supplementary concept word, anatomy supplementary concept word]	1485
19	Early Medical Intervention.mp. [mp=title, book title, abstract, original title, name of substance word, subject heading word, floating sub-heading word, keyword heading word, organism supplementary concept word, protocol supplementary concept word, rare disease supplementary concept word, unique identifier, synonyms, population supplementary concept word, anatomy supplementary concept word]	3641
20	Needs Assessment.mp. [mp=title, book title, abstract, original title, name of substance word, subject heading word, floating sub-heading word, keyword heading word, organism supplementary concept word, protocol supplementary concept word, rare disease supplementary concept word, unique identifier, synonyms, population supplementary concept word, anatomy supplementary concept word]	36288
21	Program Development.mp. [mp=title, book title, abstract, original title, name of substance word, subject heading word, floating sub-heading word, keyword heading word, organism supplementary concept word, protocol supplementary concept word, rare disease supplementary concept word, unique identifier, synonyms, population supplementary concept word, anatomy supplementary concept word]	32353
22	(Referral and Consultation).mp. [mp=title, book title, abstract, original title, name of substance word, subject heading word, floating sub-heading word, keyword heading word, organism supplementary concept word, protocol supplementary concept word, rare disease supplementary concept word, unique identifier, synonyms, population supplementary concept word, anatomy supplementary concept word]	77783
23	Pilot Projects.mp. [mp=title, book title, abstract, original title, name of substance word, subject heading word, floating sub-heading word, keyword heading word, organism supplementary concept word, protocol supplementary concept word, rare disease supplementary concept word, unique identifier, synonyms, population supplementary concept word, anatomy supplementary concept word]	146750
24	Social Welfare.mp. [mp=title, book title, abstract, original title, name of substance word, subject heading word, floating sub-heading word, keyword heading word, organism supplementary concept word, protocol supplementary concept word, rare disease supplementary concept word, unique identifier, synonyms, population supplementary concept word, anatomy supplementary concept word]	12048
25	Patient Navigation.mp. [mp=title, book title, abstract, original title, name of substance word, subject heading word, floating sub-heading word, keyword heading word, organism supplementary concept word, protocol supplementary concept word, rare disease supplementary concept word, unique identifier, synonyms, population supplementary concept word, anatomy supplementary concept word]	1469
26	Patient Advocacy.mp. [mp=title, book title, abstract, original title, name of substance word, subject heading word, floating sub-heading word, keyword heading word, organism supplementary concept word, protocol supplementary concept word, rare disease supplementary concept word, unique identifier, synonyms, population supplementary concept word, anatomy supplementary concept word]	25212
27	Inservice Training.mp. [mp=title, book title, abstract, original title, name of substance word, subject heading word, floating sub-heading word, keyword heading word, organism supplementary concept word, protocol supplementary concept word, rare disease supplementary concept word, unique identifier, synonyms, population supplementary concept word, anatomy supplementary concept word]	20870

Search	Query	Items Found
28	Staff Development.mp. [mp=title, book title, abstract, original title, name of substance word, subject heading word, floating sub-heading word, keyword heading word, organism supplementary concept word, protocol supplementary concept word, rare disease supplementary concept word, unique identifier, synonyms, population supplementary concept word, anatomy supplementary concept word]	10960
29	intervention*.ti,ab,kf.	1103956
30	(need* adj2 (assessment* or evaluat* or determin*)).ti,ab,kf.	68264
31	patient navigat*.ti,ab,kf.	1313
32	patient advoca*.ti,ab,kf.	2852
33	((staff or employee*) adj2 (develop* or train* or educat* or curricul*)).ti,ab,kf.	16752
34	((social* or socio* or communit* or neighbor* or neighbour*) adj3 (refer* or partner*)).ti,ab,kf.	14223
35	17 or 18 or 19 or 20 or 21 or 22 or 23 or 24 or 25 or 26 or 27 or 28 or 29 or 30 or 31 or 32 or 33 or 34	1476158
36	Health Services Accessibility.mp. [mp=title, book title, abstract, original title, name of substance word, subject heading word, floating sub-heading word, keyword heading word, organism supplementary concept word, protocol supplementary concept word, rare disease supplementary concept word, unique identifier, synonyms, population supplementary concept word, anatomy supplementary concept word]	85360
37	Health Equity.mp. [mp=title, book title, abstract, original title, name of substance word, subject heading word, floating sub-heading word, keyword heading word, organism supplementary concept word, protocol supplementary concept word, rare disease supplementary concept word, unique identifier, synonyms, population supplementary concept word, anatomy supplementary concept word]	7911
38	Right to Health.mp. [mp=title, book title, abstract, original title, name of substance word, subject heading word, floating sub-heading word, keyword heading word, organism supplementary concept word, protocol supplementary concept word, rare disease supplementary concept word, unique identifier, synonyms, population supplementary concept word, anatomy supplementary concept word]	1677
39	Universal Health Care.mp. [mp=title, book title, abstract, original title, name of substance word, subject heading word, floating sub-heading word, keyword heading word, organism supplementary concept word, protocol supplementary concept word, rare disease supplementary concept word, unique identifier, synonyms, population supplementary concept word, anatomy supplementary concept word]	1362
40	(primary care adj3 (access* or avail* or utiliz*)).ti,ab,kf.	3686
41	(health services adj3 (access* or avail* or utiliz*)).ti,ab,kf.	10096
42	(healthcare adj3 (access* or avail* or utiliz*)).ti,ab,kf.	19601
43	(health care adj3 (access* or avail* or utiliz*)).ti,ab,kf.	27984
44	36 or 37 or 38 or 39 or 40 or 41 or 42 or 43	137488
45	Primary Health Care.mp. [mp=title, book title, abstract, original title, name of substance word, subject heading word, floating sub-heading word, keyword heading word, organism supplementary concept word, protocol supplementary concept word, rare disease supplementary concept word, unique identifier, synonyms, population supplementary concept word, anatomy supplementary concept word]	104126
46	Comprehensive Health Care.mp. [mp=title, book title, abstract, original title, name of substance word, subject heading word, floating sub-heading word, keyword heading word, organism supplementary concept word, protocol supplementary concept word, rare disease supplementary concept word, unique identifier, synonyms, population supplementary concept word, anatomy supplementary concept word]	7459
47	General Practice.mp. [mp=title, book title, abstract, original title, name of substance word, subject heading word, floating sub-heading word, keyword heading word, organism supplementary concept word, protocol supplementary concept word, rare disease supplementary concept word, unique identifier, synonyms, population supplementary concept word, anatomy supplementary concept word]	49453
48	General Practitioners.mp. [mp=title, book title, abstract, original title, name of substance word, subject heading word, floating sub-heading word, keyword heading word, organism supplementary concept word, protocol supplementary concept word, rare disease supplementary concept word, unique identifier, synonyms, population supplementary concept word, anatomy supplementary concept word]	40736
49	Family Practice/	66938

© 2024 Viswanathan M et al. *JAMA Network Open.*

Search	Query	Items Found
50	Physicians, Family/	17169
51	Physicians, Primary Care/	4317
52	Primary Care Nursing/	565
53	Nurse Practitioners/	18795
54	Family Nurse Practitioners/	75
55	Pediatric Nurse Practitioners/	187
56	Physician Assistants/	6297
57	Family Nursing/	1564
58	Community Health Nursing/	19761
59	Community Health Centers/	7511
60	Community Mental Health Centers/	3032
61	Community Health Services/	33035
62	Community Mental Health Services/	19022
63	Community Health Workers/	6535
64	Safety-net Providers/	1317
65	primary care.ti,ab,kf.	125354
66	primary health care.ti,ab,kf.	29023
67	((family or general or primary) adj1 (medicine or practice or practitioner* or physician* or doctor* or provider* or clinic* or clinician*)).ti,ab,kf.	130334
68	36 or 37 or 38 or 39 or 40 or 41 or 42 or 43 or 44 or 45 or 46 or 47 or 48 or 49 or 50 or 51 or 52 or 53 or 54 or 55 or 56 or 57 or 58 or 59 or 60 or 61 or 62 or 63 or 64 or 65 or 66 or 67	519625
69	16 and 35 and 68	10318
70	limit 69 to (yr="1995 -Current" and (systematic reviews pre 2019 or systematic reviews))	584
71	(systematic adj3 (review or assess* or eval*)).ti.	177714
72	69 and 71	227
73	70 or 72	586
74	35 and 44	30705
75	limit 74 to (yr="1995 -Current" and (systematic reviews pre 2019 or systematic reviews))	2252
76	71 and 74	900
77	75 or 76	2257
78	73 or 77	2475
79	limit 78 to last year	318
80	"Social Determinants of Health"/	6221
81	Social Conditions/	9624
82	Social Environment/	44466
83	Social Class/	44527
84	Socioeconomic Factors/	171226
85	(social* adj1 determin*).ti,ab,kf.	13304
86	((determinant* or determinate*) adj2 health).ti,ab,kf.	14781
87	((social* or socio*) adj1 condition*).ti,ab,kf.	7803
88	((social* or socio*) adj1 environment*).ti,ab,kf.	13486
89	((social* or socio*) adj1 (factor* or gradient*)).ti,ab,kf.	45737
90	((social* or socio*) adj1 (need* or require*)).ti,ab,kf.	3223
91	((social* or socio*) adj1 (equit* or inequit* or disparit* or equal* or inequal*)).ti,ab,kf.	11327
92	((social* or socio*) adj1 (hardship* or depriv* or challeng* or difficult* or barrier* or vulnerab* or disadvantag*)).ti,ab,kf.	16764
93	((social* or socio*) adj1 risk*).ti,ab,kf.	3448
94	((social* or socio*) adj1 (status* or circumstance* or position* or class*)).ti,ab,kf.	71736
95	Food Supply/	15559
96	Hunger/	6004
97	(food adj2 (secur* or insecur* or unstable or stable or stabilit* or instabilit* or uncertain* or vulnerab* or hardship* or insufficien* or stress*)).ti,ab,kf.	14939
98	food desert*.ti,ab,kf.	278
99	Housing/	19986
100	Almshouses/	53
101	Public Housing/	1601

© 2024 Viswanathan M et al. *JAMA Network Open.*

Search	Query	Items Found
102	((hous* or home) adj3 (secur* or insecur* or unstable or stable or stabilit* or instabilit* or uncertain* or vulnerab* or hardship* or insufficien* or stress*)).ti,ab,kf.	7249
103	Homeless Persons/	9695
104	Homeless Youth/	1420
105	(homeless* or houseless*).ti,ab,kf.	12114
106	Transportation/	11764
107	Transportation Facilities/	61
108	Parking Facilities/	364
109	transportation*.ti.	4306
110	commut*.ti,ab,kf.	4199
111	Educational Status/	58403
112	Academic Failure/	57
113	Literacy/	1540
114	Reading/	25450
115	(literacy or literate or illitera*).ti,ab,kf.	29471
116	(read* adj2 (proficien* or skill* or comprehension or level*)).ti,ab,kf.	8287
117	((education* or academic* or schola* or school*) adj2 (achieve* or status or attain* or equit* or inequit* or disparit* or equal* or inequalit* or level* or background*)).ti,ab,kf.	98354
118	((education* or academic* or schola* or school*) adj2 (opportunit* or disadvantage* or advantage* or marginal* or disenfranchis* or vulnerab*)).ti,ab,kf.	4971
119	Poverty/	43432
120	Poverty Areas/	6639
121	((economic* or income* or financ*) adj2 (achieve* or status or attain* or equit* or inequit* or disparit* or equal* or inequalit* or level* or background*)).ti,ab,kf.	39681
122	((economic* or income* or financ*) adj2 (opportunit* or disadvantage* or advantage* or marginal* or disenfranchis* or vulnerab* or low or strain* or strugg* or stable or unstable or stabilit* or instabilit* or difficult* or problem*)).ti,ab,kf.	63387
123	(poverty or indigent* or indigency or impoverish*).ti.	6063
124	Employment/	50231
125	Unemployment/	7756
126	unemployment.ti,ab,kf.	11936
127	unemployed.ti,ab,kf.	9515
128	underemploy*.ti,ab,kf.	392
129	(occupation* adj2 (status or level or class)).ti,ab,kf.	7239
130	jobless*.ti,ab,kf.	303
131	workless*.ti,ab,kf.	33
132	(employment adj2 (status or securit* or insecurit* or marginal* or precarious* or terminat*)).ti,ab,kf.	10922
133	Child Care/	6085
134	(child adj2 care).ti,ab,kf.	10553
135	Social Isolation/	16078
136	(social* adj2 isolat*).ti,ab,kf.	11176
137	Legal Services/	45
138	(legal adj2 service*).ti,ab,kf.	739
139	((water or power or electric* or gas or sewer or sanit* or phone or internet or cable or satellite) adj3 (utility or utilities)).ti,ab,kf.	1444
140	Early Intervention, Educational/	3493
141	Child Development/	50129
142	Language Development/	11994
143	((child* or toddler or infant*) adj3 (educat* or develop*)).ti,ab,kf.	90820
144	80 or 81 or 82 or 83 or 84 or 85 or 86 or 87 or 88 or 89 or 90 or 91 or 92 or 93 or 94 or 95 or 96 or 97 or 98 or 99 or 100 or 101 or 102 or 103 or 104 or 105 or 106 or 107 or 108 or 109 or 110 or 111 or 112 or 113 or 114 or 115 or 116 or 117 or 118 or 119 or 120 or 121 or 122 or 123 or 124 or 125 or 126 or 127 or 128 or 129 or 130 or 131 or 132	691657
145	133 or 134 or 135 or 136 or 137 or 138 or 139 or 140 or 141 or 142 or 143	174096
146	Mass Screening/	115767
147	(Surveys and Questionnaires).mp. [mp=title, book title, abstract, original title, name of substance word, subject heading word, floating sub-heading word, keyword heading	563461

Search	Query	Items Found
	word, organism supplementary concept word, protocol supplementary concept word, rare disease supplementary concept word, unique identifier, synonyms, population supplementary concept word, anatomy supplementary concept word]	
148	screen*.ti,ab,kf.	814425
149	(instrument* or tool*).ti.	130764
150	146 or 147 or 148 or 149	1478232
151	Needs Assessment/	32365
152	Program Development/	30278
153	(Referral and Consultation).mp. [mp=title, book title, abstract, original title, name of substance word, subject heading word, floating sub-heading word, keyword heading word, organism supplementary concept word, protocol supplementary concept word, rare disease supplementary concept word, unique identifier, synonyms, population supplementary concept word, anatomy supplementary concept word]	77783
154	Pilot Projects/	146144
155	Social Welfare/	9731
156	Food Assistance/	1785
157	Public Assistance/	3012
158	Patient Navigation/	1033
159	Patient Advocacy/	24202
160	Inservice Training/	20738
161	Staff Development/	9855
162	intervention*.ti,ab,kf.	1103956
163	(need* adj2 (assessment* or evaluat* or determin*)).ti,ab,kf.	68264
164	(food adj2 (assist* or aid or help*)).ti,ab,kf.	1659
165	((hous* or home) adj2 (assist* or aid or help*)).ti,ab,kf.	2798
166	(transportation adj2 (assist* or aid or help*)).ti,ab,kf.	262
167	((education* or academic* or schola* or school*) adj2 (assist* or aid or help*)).ti,ab,kf.	4924
168	((employment or occupation* or job*) adj2 (assist* or aid or help*)).ti,ab,kf.	1156
169	((economic* or income* or financ*) adj2 (assist* or aid or help*)).ti,ab,kf.	2940
170	patient navigat*.ti,ab,kf.	1313
171	patient advoca*.ti,ab,kf.	2852
172	((staff or employee*) adj2 (develop* or train* or educat* or curricul*)).ti,ab,kf.	16752
173	((social* or socio* or communit* or neighbor* or neighbour*) adj3 (refer* or partner*)).ti,ab,kf.	14223
174	((utility or utilities) adj2 (assist* or help or aid)).ti,ab,kf.	162
175	(legal adj2 (assist* or help or aid)).ti,ab,kf.	439
176	151 or 152 or 153 or 154 or 155 or 156 or 157 or 158 or 159 or 160 or 161 or 162 or 163 or 164 or 165 or 166 or 167 or 168 or 169 or 170 or 171 or 172 or 173	1482810
177	150 or 176	2765450
178	174 or 175	600
179	Primary Health Care/	90776
180	Comprehensive Health Care/	6782
181	General Practice/	15326
182	General Practitioners/	10419
183	Family Practice/	66938
184	Physicians, Family/	17169
185	Physicians, Primary Care/	4317
186	Primary Care Nursing/	565
187	Nurse Practitioners/	18795
188	Family Nurse Practitioners/	75
189	Pediatric Nurse Practitioners/	187
190	Physician Assistants/	6297
191	Family Nursing/	1564
192	Community Health Nursing/	19761
193	Community Health Centers/	7511
194	Community Mental Health Centers/	3032
195	Community Health Services/	33035
196	Community Mental Health Services/	19022
197	Community Health Workers/	6535

© 2024 Viswanathan M et al. *JAMA Network Open.*

Search	Query	Items Found
198	Safety-net Providers/	1317
199	primary care.ti,ab,kf.	125354
200	primary health care.ti,ab,kf.	29023
201	((family or general or primary) adj1 (medicine or practice or practitioner* or physician* or doctor* or provider* or clinic* or clinician*)).ti,ab,kf.	130334
202	Patient-Centered Care/	22740
203	Patient Care Team/	69323
204	Health Services/	27476
205	"Delivery of Health Care"/	115024
206	Emergency Medicine/	15106
207	Pediatric Emergency Medicine/	491
208	exp emergency medical services/	167351
209	(emergency adj2 (medicine or servic* or room* or department* or physician* or doctor* or provider* or clinician*)).ti,ab,kf.	151355
210	179 or 180 or 181 or 182 or 183 or 184 or 185 or 186 or 187 or 188 or 189 or 190 or 191 or 192 or 193 or 194 or 195 or 196 or 197 or 198 or 199 or 200 or 201 or 202 or 203 or 204 or 205 or 206 or 207 or 208 or 209	813461
211	144 and 177 and 210	26606
212	211 and "Case Reports".sa_pubt.	173
213	211 not 212	26433
214	limit 213 to english language	24982
215	limit 214 to yr="2020 -Current"	4731
216	177 or 178	2765838
217	145 and 210 and 216	4028
218	limit 217 to case reports	100
219	217 not 218	3928
220	limit 219 to english language	3699
221	limit 220 to yr="2020 -Current"	633
222	215 or 221	5181
223	(202007* or 202008* or 202009* or 202010* or 202011* or 202012* or 2021* or 2022*).ed.	3151311
224	222 and 223	4601
225	limit 224 to last year	1522
226	"Social Determinants of Health"/	6221
227	Social Conditions/	9624
228	Social Environment/	44466
229	Social Class/	44527
230	Socioeconomic Factors/	171226
231	(social* adj1 determin*).ti,ab,kf.	13304
232	((determinant* or determinate*) adj2 health).ti,ab,kf.	14781
233	((social* or socio*) adj1 condition*).ti,ab,kf.	7803
234	((social* or socio*) adj1 environment*).ti,ab,kf.	13486
235	((social* or socio*) adj1 (factor* or gradient*)).ti,ab,kf.	45737
236	((social* or socio*) adj1 (need* or require*)).ti,ab,kf.	3223
237	((social* or socio*) adj1 (equit* or inequit* or disparit* or equal* or inequal*)).ti,ab,kf.	11327
238	((social* or socio*) adj1 (hardship* or depriv* or challeng* or difficult* or barrier* or vulnerab* or disadvantag*)).ti,ab,kf.	16764
239	((social* or socio*) adj1 risk*).ti,ab,kf.	3448
240	((social* or socio*) adj1 (status* or circumstance* or position* or class*)).ti,ab,kf.	71736
241	Food Supply/	15559
242	Hunger/	6004
243	(food adj2 (secur* or insecur* or unstable or stable or stabilit* or instabilit* or uncertain* or vulnerab* or hardship* or insufficien* or stress*)).ti,ab,kf.	14939
244	food desert*.ti,ab,kf.	278
245	Housing/	19986
246	Almshouses/	53
247	Public Housing/	1601
248	((hous* or home) adj3 (secur* or insecur* or unstable or stable or stabilit* or instabilit* or uncertain* or vulnerab* or hardship* or insufficien* or stress*)).ti,ab,kf.	7249

© 2024 Viswanathan M et al. *JAMA Network Open.*

Search	Query	Items Found
249	Homeless Persons/	9695
250	Homeless Youth/	1420
251	(homeless* or houseless*).ti,ab,kf.	12114
252	Transportation/	11764
253	Transportation Facilities/	61
254	Parking Facilities/	364
255	transportation*.ti.	4306
256	commut*.ti,ab,kf.	4199
257	Educational Status/	58403
258	Academic Failure/	57
259	Literacy/	1540
260	Reading/	25450
261	(literacy or literate or illitera*).ti,ab,kf.	29471
262	(read* adj2 (proficien* or skill* or comprehension or level*)).ti,ab,kf.	8287
263	((education* or academic* or schola* or school*) adj2 (achieve* or status or attain* or equit* or inequit* or disparit* or equal* or inequalit* or level* or background*)).ti,ab,kf.	98354
264	((education* or academic* or schola* or school*) adj2 (opportunit* or disadvantage* or advantage* or marginal* or disenfranchis* or vulnerab*)).ti,ab,kf.	4971
265	Poverty/	43432
266	Poverty Areas/	6639
267	((economic* or income* or financ*) adj2 (achieve* or status or attain* or equit* or inequit* or disparit* or equal* or inequalit* or level* or background*)).ti,ab,kf.	39681
268	((economic* or income* or financ*) adj2 (opportunit* or disadvantage* or advantage* or marginal* or disenfranchis* or vulnerab* or low or strain* or strugg* or stable or unstable or stabilit* or instabilit* or difficult* or problem*)).ti,ab,kf.	63387
269	(poverty or indigent* or indigency or impoverish*).ti.	6063
270	Employment/	50231
271	Unemployment/	7756
272	unemployment.ti,ab,kf.	11936
273	unemployed.ti,ab,kf.	9515
274	underemploy*.ti,ab,kf.	392
275	(occupation* adj2 (status or level or class)).ti,ab,kf.	7239
276	jobless*.ti,ab,kf.	303
277	workless*.ti,ab,kf.	33
278	(employment adj2 (status or securit* or insecurit* or marginal* or precarious* or terminat*)).ti,ab,kf.	10922
279	Child Care/	6085
280	(child adj2 care).ti,ab,kf.	10553
281	Social Isolation/	16078
282	(social* adj2 isolat*).ti,ab,kf.	11176
283	Legal Services/	45
284	(legal adj2 service*).ti,ab,kf.	739
285	((water or power or electric* or gas or sewer or sanit* or phone or internet or cable or satellite) adj3 (utility or utilities)).ti,ab,kf.	1444
286	Early Intervention, Educational/	3493
287	Child Development/	50129
288	Language Development/	11994
289	((child* or toddler or infant*) adj3 (educat* or develop*)).ti,ab,kf.	90820
290	226 or 227 or 228 or 229 or 230 or 231 or 232 or 233 or 234 or 235 or 236 or 237 or 238 or 239 or 240 or 241 or 242 or 243 or 244 or 245 or 246 or 247 or 248 or 249 or 250 or 251 or 252 or 253 or 254 or 255 or 256 or 257 or 258 or 259 or 260 or 261 or 262 or 263 or 264 or 265 or 266 or 267 or 268 or 269 or 270 or 271 or 272 or 273 or 274 or 275 or 276 or 277 or 278	691657
291	279 or 280 or 281 or 282 or 283 or 284 or 285 or 286 or 287 or 288 or 289	174096
292	Mass Screening/	115767
293	(Surveys and Questionnaires).mp. [mp=title, book title, abstract, original title, name of substance word, subject heading word, floating sub-heading word, keyword heading word, organism supplementary concept word, protocol supplementary concept word,	563461

© 2024 Viswanathan M et al. *JAMA Network Open.*

Search	Query	Items Found
	rare disease supplementary concept word, unique identifier, synonyms, population supplementary concept word, anatomy supplementary concept word]	
294	screen*.ti,ab,kf.	814425
295	(instrument* or tool*).ti.	130764
296	292 or 293 or 294 or 295	1478232
297	Needs Assessment/	32365
298	Program Development/	30278
299	(Referral and Consultation).mp. [mp=title, book title, abstract, original title, name of substance word, subject heading word, floating sub-heading word, keyword heading word, organism supplementary concept word, protocol supplementary concept word, rare disease supplementary concept word, unique identifier, synonyms, population supplementary concept word, anatomy supplementary concept word]	77783
300	Pilot Projects/	146144
301	Social Welfare/	9731
302	Food Assistance/	1785
303	Public Assistance/	3012
304	Patient Navigation/	1033
305	Patient Advocacy/	24202
306	Inservice Training/	20738
307	Staff Development/	9855
308	intervention*.ti,ab,kf.	1103956
309	(need* adj2 (assessment* or evaluat* or determin*)).ti,ab,kf.	68264
310	(food adj2 (assist* or aid or help*)).ti,ab,kf.	1659
311	((hous* or home) adj2 (assist* or aid or help*)).ti,ab,kf.	2798
312	(transportation adj2 (assist* or aid or help*)).ti,ab,kf.	262
313	((education* or academic* or schola* or school*) adj2 (assist* or aid or help*)).ti,ab,kf.	4924
314	((employment or occupation* or job*) adj2 (assist* or aid or help*)).ti,ab,kf.	1156
315	((economic* or income* or financ*) adj2 (assist* or aid or help*)).ti,ab,kf.	2940
316	patient navigat*.ti,ab,kf.	1313
317	patient advoca*.ti,ab,kf.	2852
318	((staff or employee*) adj2 (develop* or train* or educat* or curricul*)).ti,ab,kf.	16752
319	((social* or socio* or communit* or neighbor* or neighbour*) adj3 (refer* or partner*)).ti,ab,kf.	14223
320	((utility or utilities) adj2 (assist* or help or aid)).ti,ab,kf.	162
321	(legal adj2 (assist* or help or aid)).ti,ab,kf.	439
322	297 or 298 or 299 or 300 or 301 or 302 or 303 or 304 or 305 or 306 or 307 or 308 or 309 or 310 or 311 or 312 or 313 or 314 or 315 or 316 or 317 or 318 or 319	1482810
323	296 or 322	2765450
324	320 or 321	600
325	Primary Health Care/	90776
326	Comprehensive Health Care/	6782
327	General Practice/	15326
328	General Practitioners/	10419
329	Family Practice/	66938
330	Physicians, Family/	17169
331	Physicians, Primary Care/	4317
332	Primary Care Nursing/	565
333	Nurse Practitioners/	18795
334	Family Nurse Practitioners/	75
335	Pediatric Nurse Practitioners/	187
336	Physician Assistants/	6297
337	Family Nursing/	1564
338	Community Health Nursing/	19761
339	Community Health Centers/	7511
340	Community Mental Health Centers/	3032
341	Community Health Services/	33035
342	Community Mental Health Services/	19022
343	Community Health Workers/	6535
344	Safety-net Providers/	1317

Search	Query	Items Found
345	primary care.ti,ab,kf.	125354
346	primary health care.ti,ab,kf.	29023
347	((family or general or primary) adj1 (medicine or practice or practitioner* or physician* or doctor* or provider* or clinic* or clinician*)).ti,ab,kf.	130334
348	Patient-Centered Care/	22740
349	Patient Care Team/	69323
350	Health Services/	27476
351	"Delivery of Health Care"/	115024
352	Emergency Medicine/	15106
353	Pediatric Emergency Medicine/	491
354	exp emergency medical services/	167351
355	(emergency adj2 (medicine or servic* or room* or department* or physician* or doctor* or provider* or clinician*)).ti,ab,kf.	151355
356	325 or 326 or 327 or 328 or 329 or 330 or 331 or 332 or 333 or 334 or 335 or 336 or 337 or 338 or 339 or 340 or 341 or 342 or 343 or 344 or 345 or 346 or 347 or 348 or 349 or 350 or 351 or 352 or 353 or 354 or 355	813461
357	290 and 323 and 356	26606
358	357 and "Case Reports".sa_pubt.	173
359	357 not 358	26433
360	limit 359 to english language	24982
361	limit 360 to yr="2020 -Current"	4731
362	323 or 324	2765838
363	291 and 356 and 362	4028
364	limit 363 to case reports	100
365	363 not 364	3928
366	limit 365 to english language	3699
367	limit 366 to yr="2020 -Current"	633
368	361 or 367	5181
369	limit 368 to last year	1969

eTable 4. Cochrane Library (Including Both Cochrane Database of Systematic Reviews and Cochrane Central Register of Controlled Trials) Search String and Yield for Food Insecurity, Housing, Education and Literacy, Financial Strain, Employment, Transportation, Utilities, Social Isolation, Early Childhood Development, Legal Services, and Childcare (April 6, 2023)

ID	Search	Hits
#1	social*:ti,ab,kw near/1 determin*:ti,ab,kw	611
#2	(determinant* or determinate*):ti,ab,kw near/2 health:ti,ab,kw	649
#3	(social* or socio*):ti,ab,kw near/1 condition*:ti,ab,kw	480
#4	(social* or socio*):ti,ab,kw near/1 environment*:ti,ab,kw	1845
#5	(social* or socio*):ti,ab,kw near/1 (factor* or gradient*):ti,ab,kw	6270
#6	(social* or socio*):ti,ab,kw near/1 (need* or require*):ti,ab,kw	326
#7	(social* or socio*):ti,ab,kw near/1 (equit* or inequit* or disparit* or equal* or inequal*):ti,ab,kw	268
#8	(social* or socio*):ti,ab,kw near/1 (hardship* or depriv* or challeng* or difficult* or barrier* or vulnerab* or disadvantag*):ti,ab,kw	1478
#9	(social* or socio*):ti,ab,kw near/1 risk*:ti,ab,kw	302
#10	(social* or socio*):ti,ab,kw near/1 (status* or circumstance* or position* or class* or standing):ti,ab,kw	6356
#11	food*:ti,ab,kw near/2 (supply or secur* or insecur* or unstable or stable or stabilit* or instabilit* or uncertain* or vulnerab* or hardship* or insufficien* or stress*):ti,ab,kw	1298
#12	food:ti,ab,kw next desert*:ti,ab,kw	12
#13	(hous* or home):ti,ab,kw near/3 (secur* or insecur* or unstable or stable or stabilit* or instabilit* or uncertain* or vulnerab* or hardship* or insufficien* or stress*):ti,ab,kw	900
#14	(homeless* or houseless*):ti,ab,kw	1131
#15	Transportation*:ti,ab,kw	2059
#16	commut*:ti,ab,kw	272
#17	(literacy or literate or illitera*):ti,ab,kw	6892
#18	read*:ti,ab,kw near/2 (proficien* or skill* or comprehension or level*):ti,ab,kw	1118
#19	(education* or academic* or schola* or school*):ti,ab,kw near/2 (achieve* or fail* or status or attain* or equit* or inequit* or disparit* or equal* or inequalit* or level* or background*):ti,ab,kw	12480
#20	(education* or academic* or schola* or school*):ti,ab,kw near/2 (opportunit* or disadvantage* or advantage* or marginal* or disenfranchis* or vulnerab*):ti,ab,kw	297
#21	(economic* or income* or financ*):ti,ab,kw near/2 (achieve* or status or attain* or equit* or inequit* or disparit* or equal* or inequalit* or level* or background*):ti,ab,kw	2630
#22	(economic* or income* or financ*):ti,ab,kw near/2 (opportunit* or disadvantage* or advantage* or marginal* or disenfranchis* or vulnerab* or low or strain* or strugg* or stable or unstable or stabilit* or instabilit* or difficult* or problem* or stress*):ti,ab,kw	7408
#23	(poverty or indigent* or indigency or impoverish*):ti,ab,kw	3758
#24	unemployment:ti,ab,kw	945
#25	unemployed:ti,ab,kw	717
#26	underemployed:ti,ab,kw	10
#27	(occupation* or job):ti,ab,kw near/2 (status or level or class):ti,ab,kw	559
#28	jobless*:ti,ab,kw	5
#29	workless*:ti,ab,kw	2
#30	(employment or job or occupation*):ti,ab,kw near/2 (status or securit* or insecurit* or marginal* or precarious* or terminat*):ti,ab,kw	1688
#31	child:ti,ab,kw near/2 care:ti,ab,kw	4455
#32	social*:ti,ab,kw near/2 isolat*:ti,ab,kw	1326
#33	legal:ti,ab,kw near/2 service*:ti,ab,kw	42
#34	(water or power or electric* or gas or sewer or sanit* or phone or internet or cable or satellite):ti,ab,kw near/3 (utility or utilities):ti,ab,kw	65
#35	(child* or toddler or infant*):ti,ab,kw near/3 (educat* or develop* or language*):ti,ab,kw	14635
#36	#5 OR #6 OR #7 OR #8 OR #9 OR #10 OR #11 OR #12 OR #13 OR #14 OR #15 OR #16 OR #17 OR #18 OR #19 OR #20 OR #21 OR #22 OR #23 OR #24 OR #25 OR #26 OR #27 OR #28 OR #29 OR #30	45092
#37	#31 OR #32 OR #33 OR #34 OR #35	19706
#38	screen*:ti,ab,kw	92789

© 2024 Viswanathan M et al. *JAMA Network Open.*

ID	Search	Hits
#39	(instrument* or tool*):ti	7220
#40	#38 OR #39	99233
#41	intervention*:ti,ab,kw	534075
#42	need*:ti,ab,kw near/2 (assessment* or evaluat* or determin*):ti,ab,kw	10318
#43	program*:ti,ab,kw near/2 develop*:ti,ab,kw	4926
#44	pilot:ti,ab,kw next project*:ti,ab,kw	26244
#45	food:ti,ab,kw near/2 (assist* or aid or help*):ti,ab,kw	346
#46	(hous* or home):ti,ab,kw near/2 (assist* or aid or help*):ti,ab,kw	475
#47	transportation*:ti,ab,kw near/2 (assist* or aid or help*):ti,ab,kw	36
#48	(education* or academic* or schola* or school*):ti,ab,kw near/2 (assist* or aid or help*):ti,ab,kw	935
#49	(employment or occupation* or job*):ti,ab,kw near/2 (assist* or aid or help*):ti,ab,kw	111
#50	(economic* or income* or financ*):ti,ab,kw near/2 (assist* or aid or help*):ti,ab,kw	378
#51	patient*:ti,ab,kw near/1 navigat*:ti,ab,kw	759
#52	patient*:ti,ab,kw near/2 advoca*:ti,ab,kw	447
#53	(staff or employee*):ti,ab,kw near/2 (develop* or train* or educat* or curricul*):ti,ab,kw	3157
#54	(social* or socio* or communit* or neighbor* or neighbour*):ti,ab,kw near/3 (refer* or partner*):ti,ab,kw	1895
#55	(utility or utilities):ti,ab,kw near/2 (assist* or help or aid):ti,ab,kw	13
#56	legal:ti,ab,kw near/2 (assist* or help or aid):ti,ab,kw	27
#57	#41 OR #42 OR #43 OR #44 OR #45 OR #46 OR #47 OR #48 OR #49 OR #50 OR #51 OR #52 OR #53 OR #54 OR #55 OR #56	560720
#58	#40 OR #57	611794
#59	#36 AND #58	29582
#60	#37 AND #58	11684
#61	primary:ti,ab,kw next care:ti,ab,kw	21368
#62	comprehensive:ti,ab,kw next care:ti,ab,kw	373
#63	"primary health care":ti,ab,kw	8252
#64	"comprehensive health care":ti,ab,kw	113
#65	comprehensive:ti,ab,kw next healthcare:ti,ab,kw	19
#66	primary:ti,ab,kw next healthcare:ti,ab,kw	882
#67	(safety-net:ti,ab,kw or "safety net":ti,ab,kw) next clinic*:ti,ab,kw	94
#68	"community health center":ti,ab,kw	344
#69	"community health centers":ti,ab,kw	764
#70	"federally qualified health center":ti,ab,kw	176
#71	"federally qualified health centers":ti,ab,kw	164
#72	fqhc:ti,ab,kw	109
#73	(family or general or primary):ti,ab,kw near/2 (medicine or practice or practitioner* or physician* or doctor* or provider* or clinic* or clinician* or nurs*):ti,ab,kw	32580
#74	emergency:ti,ab,kw near/2 (medicine or servic* or room* or department* or physician* or doctor* or provider* or clinician*):ti,ab,kw	19849
#75	#61 OR #62 OR #63 OR #64 OR #65 OR #66 OR #67 OR #68 OR #69 OR #70 OR #71 OR #72 OR #73 OR #74	64380
#76	#59 AND #75	4018
#77	#60 AND #75	1116
#78	#76 OR #77 with Cochrane Library publication date in The last year	449

eTable 5. Ovid MEDLINE® Search String and Yield for Interpersonal Violence MEDLINE Search (Ovid MEDLINE®) (April 6, 2023)

Search	Query	Items Found
1	Physical Abuse/	1131
2	Gun Violence/	439
3	gender based violence.mp. [mp=title, book title, abstract, original title, name of substance word, subject heading word, floating sub-heading word, keyword heading word, organism supplementary concept word, protocol supplementary concept word,	1533

© 2024 Viswanathan M et al. *JAMA Network Open.*

Search	Query	Items Found
	rare disease supplementary concept word, unique identifier, synonyms, population supplementary concept word, anatomy supplementary concept word]	
4	Elder Abuse.mp. [mp=title, book title, abstract, original title, name of substance word, subject heading word, floating sub-heading word, keyword heading word, organism supplementary concept word, protocol supplementary concept word, rare disease supplementary concept word, unique identifier, synonyms, population supplementary concept word, anatomy supplementary concept word]	3088
5	rape.mp. [mp=title, book title, abstract, original title, name of substance word, subject heading word, floating sub-heading word, keyword heading word, organism supplementary concept word, protocol supplementary concept word, rare disease supplementary concept word, unique identifier, synonyms, population supplementary concept word, anatomy supplementary concept word]	11335
6	workplace violence.mp. [mp=title, book title, abstract, original title, name of substance word, subject heading word, floating sub-heading word, keyword heading word, organism supplementary concept word, protocol supplementary concept word, rare disease supplementary concept word, unique identifier, synonyms, population supplementary concept word, anatomy supplementary concept word]	2183
7	torture.mp. [mp=title, book title, abstract, original title, name of substance word, subject heading word, floating sub-heading word, keyword heading word, organism supplementary concept word, protocol supplementary concept word, rare disease supplementary concept word, unique identifier, synonyms, population supplementary concept word, anatomy supplementary concept word]	2871
8	((elder* or geriatric* or aged or interpersonal or gun* or workplace) adj2 (violen* or abus* or neglect* or maltreat* or batter*)).ti.	3282
9	1 or 2 or 3 or 4 or 5 or 6 or 7 or 8	23012
10	Mass Screening.mp. [mp=title, book title, abstract, original title, name of substance word, subject heading word, floating sub-heading word, keyword heading word, organism supplementary concept word, protocol supplementary concept word, rare disease supplementary concept word, unique identifier, synonyms, population supplementary concept word, anatomy supplementary concept word]	118218
11	Anonymous Testing.mp. [mp=title, book title, abstract, original title, name of substance word, subject heading word, floating sub-heading word, keyword heading word, organism supplementary concept word, protocol supplementary concept word, rare disease supplementary concept word, unique identifier, synonyms, population supplementary concept word, anatomy supplementary concept word]	545
12	Mass Chest X-Ray.mp. [mp=title, book title, abstract, original title, name of substance word, subject heading word, floating sub-heading word, keyword heading word, organism supplementary concept word, protocol supplementary concept word, rare disease supplementary concept word, unique identifier, synonyms, population supplementary concept word, anatomy supplementary concept word]	1960
13	Multiphasic Screening.mp. [mp=title, book title, abstract, original title, name of substance word, subject heading word, floating sub-heading word, keyword heading word, organism supplementary concept word, protocol supplementary concept word, rare disease supplementary concept word, unique identifier, synonyms, population supplementary concept word, anatomy supplementary concept word]	1158
14	risk.mp. [mp=title, book title, abstract, original title, name of substance word, subject heading word, floating sub-heading word, keyword heading word, organism supplementary concept word, protocol supplementary concept word, rare disease supplementary concept word, unique identifier, synonyms, population supplementary concept word, anatomy supplementary concept word]	2792981
15	logistic models.mp. [mp=title, book title, abstract, original title, name of substance word, subject heading word, floating sub-heading word, keyword heading word, organism supplementary concept word, protocol supplementary concept word, rare disease supplementary concept word, unique identifier, synonyms, population supplementary concept word, anatomy supplementary concept word]	154031
16	Protective Factors.mp. [mp=title, book title, abstract, original title, name of substance word, subject heading word, floating sub-heading word, keyword heading word, organism supplementary concept word, protocol supplementary concept word, rare disease supplementary concept word, unique identifier, synonyms, population supplementary concept word, anatomy supplementary concept word]	17148

© 2024 Viswanathan M et al. *JAMA Network Open.*

Search	Query	Items Found
17	Risk Assessment.mp. [mp=title, book title, abstract, original title, name of substance word, subject heading word, floating sub-heading word, keyword heading word, organism supplementary concept word, protocol supplementary concept word, rare disease supplementary concept word, unique identifier, synonyms, population supplementary concept word, anatomy supplementary concept word]	341398
18	Adverse Outcome Pathways.mp. [mp=title, book title, abstract, original title, name of substance word, subject heading word, floating sub-heading word, keyword heading word, organism supplementary concept word, protocol supplementary concept word, rare disease supplementary concept word, unique identifier, synonyms, population supplementary concept word, anatomy supplementary concept word]	644
19	"Healthcare Failure Mode and Effect Analysis".mp. [mp=title, book title, abstract, original title, name of substance word, subject heading word, floating sub-heading word, keyword heading word, organism supplementary concept word, protocol supplementary concept word, rare disease supplementary concept word, unique identifier, synonyms, population supplementary concept word, anatomy supplementary concept word]	217
20	Risk Factors.mp. [mp=title, book title, abstract, original title, name of substance word, subject heading word, floating sub-heading word, keyword heading word, organism supplementary concept word, protocol supplementary concept word, rare disease supplementary concept word, unique identifier, synonyms, population supplementary concept word, anatomy supplementary concept word]	1155293
21	(screen* or risk).ti.	677701
22	10 or 11 or 12 or 13 or 14 or 15 or 16 or 17 or 18 or 19 or 20 or 21	3040838
23	9 and 22	5508
24	exp "Surveys and Questionnaires"/	1203238
25	exp Diagnosis/	9312808
26	interven*.ti.	169091
27	24 or 25 or 26	10129523
28	9 and 27	5227
29	23 or 28	8904
30	limit 29 to english language	8410
31	limit 30 to last year	703

eTable 6. Cochrane Library (Including Both Cochrane Database of Systematic Reviews and Cochrane Central Register of Controlled Trials) Search String and Yield for Interpersonal Violence (April 6, 2023)

ID	Search	Hits
#1	physical abuse	2676
#2	gun violence	46
#3	gender based violence	537
#4	elder abuse	1059
#5	rape	455
#6	workplace violence	105
#7	torture	90
#8	((elder* or geriatric* or aged or interpersonal or gun* or workplace) NEAR/2 (violen* or abus* or neglect* or maltreat* or batter*)):ti	119
#9	#1 OR #2 OR #3 OR #4 OR #5 OR #6 OR #7 OR #8	4189
#10	Mass Screening	11693
#11	Anonymous Testing	372
#12	Mass Chest X-Ray	624
#13	Multiphasic Screening	61
#14	risk	286855
#15	logistic models	11861
#16	Risk Assessment	86883
#17	Adverse Outcome Pathways	1888
#18	"Healthcare Failure Mode and Effect Analysis"	4
#19	Risk Factors	96047

© 2024 Viswanathan M et al. *JAMA Network Open.*

ID	Search	Hits
#20	#10 OR #11 OR #12 OR #13 OR #14 OR #15 OR #16 OR #17 OR #18 OR #19	301025
#21	#9 AND #20	1993
#22	MeSH descriptor: [Surveys and Questionnaires] explode all trees	69959
#23	MeSH descriptor: [Diagnosis] explode all trees	442250
#24	interven*:ti	75859
#25	#22 OR #23 OR #24	529402
#26	#9 AND #25	1288
#27	#26 OR #21 with Cochrane Library publication date in The last year	179

eTable 7. Ovid MEDLINE® Search String and Yield for Access to Care MEDLINE Search (Ovid MEDLINE®) (February 7, 2023)

ID	Search	Hits
1	Social Determinants of Health.mp. [mp=title, book title, abstract, original title, name of substance word, subject heading word, floating sub-heading word, keyword heading word, organism supplementary concept word, protocol supplementary concept word, rare disease supplementary concept word, unique identifier, synonyms]	11608
2	Social Conditions.mp. [mp=title, book title, abstract, original title, name of substance word, subject heading word, floating sub-heading word, keyword heading word, organism supplementary concept word, protocol supplementary concept word, rare disease supplementary concept word, unique identifier, synonyms]	11974
3	Social Environment.mp. [mp=title, book title, abstract, original title, name of substance word, subject heading word, floating sub-heading word, keyword heading word, organism supplementary concept word, protocol supplementary concept word, rare disease supplementary concept word, unique identifier, synonyms]	49166
4	Social Class.mp. [mp=title, book title, abstract, original title, name of substance word, subject heading word, floating sub-heading word, keyword heading word, organism supplementary concept word, protocol supplementary concept word, rare disease supplementary concept word, unique identifier, synonyms]	49146
5	Socioeconomic Factors.mp. [mp=title, book title, abstract, original title, name of substance word, subject heading word, floating sub-heading word, keyword heading word, organism supplementary concept word, protocol supplementary concept word, rare disease supplementary concept word, unique identifier, synonyms]	177322
6	(social* adj1 determin*).ti,ab,kf.	12879
7	((determinant* or determinate*) adj2 health).ti,ab,kf.	14378
8	((social* or socio*) adj1 condition*).ti,ab,kf.	7744
9	((social* or socio*) adj1 environment*).ti,ab,kf.	13299
10	((social* or socio*) adj1 (factor* or gradient*)).ti,ab,kf.	45226
11	((social* or socio*) adj1 (need* or require*)).ti,ab,kf.	3153
12	((social* or socio*) adj1 (equit* or inequit* or disparit* or equal* or inequal*)).ti,ab,kf.	11125
13	((social* or socio*) adj1 (hardship* or depriv* or challeng* or difficult* or barrier* or vulnerab* or disadvantag*)).ti,ab,kf.	16495
14	((social* or socio*) adj1 risk*).ti,ab,kf.	3391
15	((social* or socio*) adj1 (status* or circumstance* or position* or class* or standing)).ti,ab,kf.	71354
16	1 or 2 or 3 or 4 or 5 or 6 or 7 or 8 or 9 or 10 or 11 or 12 or 13 or 14 or 15	347520
17	Early Intervention, Educational.mp. [mp=title, book title, abstract, original title, name of substance word, subject heading word, floating sub-heading word, keyword heading word, organism supplementary concept word, protocol supplementary concept word, rare disease supplementary concept word, unique identifier, synonyms]	3476
18	Internet-Based Intervention.mp. [mp=title, book title, abstract, original title, name of substance word, subject heading word, floating sub-heading word, keyword heading word, organism supplementary concept word, protocol supplementary concept word, rare disease supplementary concept word, unique identifier, synonyms]	1456
19	Early Medical Intervention.mp. [mp=title, book title, abstract, original title, name of substance word, subject heading word, floating sub-heading word, keyword heading word, organism supplementary concept word, protocol supplementary concept word, rare disease supplementary concept word, unique identifier, synonyms]	3635

© 2024 Viswanathan M et al. *JAMA Network Open.*

ID	Search	Hits
20	Needs Assessment.mp. [mp=title, book title, abstract, original title, name of substance word, subject heading word, floating sub-heading word, keyword heading word, organism supplementary concept word, protocol supplementary concept word, rare disease supplementary concept word, unique identifier, synonyms]	36196
21	Program Development.mp. [mp=title, book title, abstract, original title, name of substance word, subject heading word, floating sub-heading word, keyword heading word, organism supplementary concept word, protocol supplementary concept word, rare disease supplementary concept word, unique identifier, synonyms]	32310
22	(Referral and Consultation).mp. [mp=title, book title, abstract, original title, name of substance word, subject heading word, floating sub-heading word, keyword heading word, organism supplementary concept word, protocol supplementary concept word, rare disease supplementary concept word, unique identifier, synonyms]	77439
23	Pilot Projects.mp. [mp=title, book title, abstract, original title, name of substance word, subject heading word, floating sub-heading word, keyword heading word, organism supplementary concept word, protocol supplementary concept word, rare disease supplementary concept word, unique identifier, synonyms]	145708
24	Social Welfare.mp. [mp=title, book title, abstract, original title, name of substance word, subject heading word, floating sub-heading word, keyword heading word, organism supplementary concept word, protocol supplementary concept word, rare disease supplementary concept word, unique identifier, synonyms]	12010
25	Patient Navigation.mp. [mp=title, book title, abstract, original title, name of substance word, subject heading word, floating sub-heading word, keyword heading word, organism supplementary concept word, protocol supplementary concept word, rare disease supplementary concept word, unique identifier, synonyms]	1452
26	Patient Advocacy.mp. [mp=title, book title, abstract, original title, name of substance word, subject heading word, floating sub-heading word, keyword heading word, organism supplementary concept word, protocol supplementary concept word, rare disease supplementary concept word, unique identifier, synonyms]	25181
27	Inservice Training.mp. [mp=title, book title, abstract, original title, name of substance word, subject heading word, floating sub-heading word, keyword heading word, organism supplementary concept word, protocol supplementary concept word, rare disease supplementary concept word, unique identifier, synonyms]	20867
28	Staff Development.mp. [mp=title, book title, abstract, original title, name of substance word, subject heading word, floating sub-heading word, keyword heading word, organism supplementary concept word, protocol supplementary concept word, rare disease supplementary concept word, unique identifier, synonyms]	10946
29	intervention*.ti,ab,kf.	1090783
30	(need* adj2 (assessment* or evaluat* or determin*)).ti,ab,kf.	67605
31	patient navigat*.ti,ab,kf.	1293
32	patient advoca*.ti,ab,kf.	2807
33	((staff or employee*) adj2 (develop* or train* or educat* or curricul*)).ti,ab,kf.	16607
34	((social* or socio* or communit* or neighbor* or neighbour*) adj3 (refer* or partner*)).ti,ab,kf.	14034
35	17 or 18 or 19 or 20 or 21 or 22 or 23 or 24 or 25 or 26 or 27 or 28 or 29 or 30 or 31 or 32 or 33 or 34	1461120
36	Health Services Accessibility.mp. [mp=title, book title, abstract, original title, name of substance word, subject heading word, floating sub-heading word, keyword heading word, organism supplementary concept word, protocol supplementary concept word, rare disease supplementary concept word, unique identifier, synonyms]	85007
37	Health Equity.mp. [mp=title, book title, abstract, original title, name of substance word, subject heading word, floating sub-heading word, keyword heading word, organism supplementary concept word, protocol supplementary concept word, rare disease supplementary concept word, unique identifier, synonyms]	7597
38	Right to Health.mp. [mp=title, book title, abstract, original title, name of substance word, subject heading word, floating sub-heading word, keyword heading word, organism supplementary concept word, protocol supplementary concept word, rare disease supplementary concept word, unique identifier, synonyms]	1652
39	Universal Health Care.mp. [mp=title, book title, abstract, original title, name of substance word, subject heading word, floating sub-heading word, keyword heading	1344

© 2024 Viswanathan M et al. *JAMA Network Open.*

ID	Search	Hits
	word, organism supplementary concept word, protocol supplementary concept word, rare disease supplementary concept word, unique identifier, synonyms]	
40	(primary care adj3 (access* or avail* or utiliz*)).ti,ab,kf.	3649
41	(health services adj3 (access* or avail* or utiliz*)).ti,ab,kf.	9929
42	(healthcare adj3 (access* or avail* or utiliz*)).ti,ab,kf.	19133
43	(health care adj3 (access* or avail* or utiliz*)).ti,ab,kf.	27667
44	36 or 37 or 38 or 39 or 40 or 41 or 42 or 43	136048
45	Primary Health Care.mp. [mp=title, book title, abstract, original title, name of substance word, subject heading word, floating sub-heading word, keyword heading word, organism supplementary concept word, protocol supplementary concept word, rare disease supplementary concept word, unique identifier, synonyms]	103499
46	Comprehensive Health Care.mp. [mp=title, book title, abstract, original title, name of substance word, subject heading word, floating sub-heading word, keyword heading word, organism supplementary concept word, protocol supplementary concept word, rare disease supplementary concept word, unique identifier, synonyms]	7445
47	General Practice.mp. [mp=title, book title, abstract, original title, name of substance word, subject heading word, floating sub-heading word, keyword heading word, organism supplementary concept word, protocol supplementary concept word, rare disease supplementary concept word, unique identifier, synonyms]	49239
48	General Practitioners.mp. [mp=title, book title, abstract, original title, name of substance word, subject heading word, floating sub-heading word, keyword heading word, organism supplementary concept word, protocol supplementary concept word, rare disease supplementary concept word, unique identifier, synonyms]	40473
49	Family Practice/	66843
50	Physicians, Family/	17134
51	Physicians, Primary Care/	4303
52	Primary Care Nursing/	564
53	Nurse Practitioners/	18761
54	Family Nurse Practitioners/	75
55	Pediatric Nurse Practitioners/	189
56	Physician Assistants/	6271
57	Family Nursing/	1565
58	Community Health Nursing/	19759
59	Community Health Centers/	7500
60	Community Mental Health Centers/	3029
61	Community Health Services/	32991
62	Community Mental Health Services/	19012
63	Community Health Workers/	6477
64	Safety-net Providers/	1305
65	primary care.ti,ab,kf.	124201
66	primary health care.ti,ab,kf.	28774
67	((family or general or primary) adj1 (medicine or practice or practitioner* or physician* or doctor* or provider* or clinic* or clinician*)).ti,ab,kf.	129590
68	36 or 37 or 38 or 39 or 40 or 41 or 42 or 43 or 44 or 45 or 46 or 47 or 48 or 49 or 50 or 51 or 52 or 53 or 54 or 55 or 56 or 57 or 58 or 59 or 60 or 61 or 62 or 63 or 64 or 65 or 66 or 67	516321
69	16 and 35 and 68	10226
70	limit 69 to (yr="1995 -Current" and (systematic reviews pre 2019 or systematic reviews))	574
71	(systematic adj3 (review or assess* or eval*)).ti.	173525
72	69 and 71	222
73	70 or 72	576
74	35 and 44	30354
75	limit 74 to (yr="1995 -Current" and (systematic reviews pre 2019 or systematic reviews))	2216
76	71 and 74	888
77	75 or 76	2221
78	73 or 77	2436
79	limit 78 to last year	279

© 2024 Viswanathan M et al. *JAMA Network Open.*

eTable 8. Cochrane Library (Including Both Cochrane Database of Systematic Reviews and Cochrane Central Register of Controlled Trials) Search String and Yield for Access to Care (February 1, 2023)

ID	Search	Hits
#1	social*:ti,ab,kw near/1 determin*:ti,ab,kw	576
#2	(determinant* or determinate*):ti,ab,kw near/2 health:ti,ab,kw	612
#3	(social* or socio*):ti,ab,kw near/1 condition*:ti,ab,kw	473
#4	(social* or socio*):ti,ab,kw near/1 environment*:ti,ab,kw	1827
#5	(social* or socio*):ti,ab,kw near/1 (factor* or gradient*):ti,ab,kw	6230
#6	(social* or socio*):ti,ab,kw near/1 (need* or require*):ti,ab,kw	316
#7	(social* or socio*):ti,ab,kw near/1 (equit* or inequit* or disparit* or equal* or inequal*):ti,ab,kw	255
#8	(social* or socio*):ti,ab,kw near/1 (hardship* or depriv* or challeng* or difficult* or barrier* or vulnerab* or disadvantag*):ti,ab,kw	1448
#9	(social* or socio*):ti,ab,kw near/1 risk*:ti,ab,kw	301
#10	(social* or socio*):ti,ab,kw near/1 (status* or circumstance* or position* or class* or standing):ti,ab,kw	6158
#11	#1 OR #2 OR #3 OR #4 OR #5 OR #6 OR #7 OR #8 OR #9 OR #10	15875
#12	intervention*:ti,ab,kw	522797
#13	need*:ti,ab,kw near/2 (assessment* or evaluat* or determin*):ti,ab,kw	10181
#14	program*:ti,ab,kw near/2 develop*:ti,ab,kw	4863
#15	pilot:ti,ab,kw next project*:ti,ab,kw	26017
#16	patient*:ti,ab,kw near/1 navigat*:ti,ab,kw	742
#17	patient*:ti,ab,kw near/2 advoca*:ti,ab,kw	440
#18	(staff or employee*):ti,ab,kw near/2 (develop* or train* or educat* or curricul*):ti,ab,kw	3114
#19	(social* or socio* or communit* or neighbor* or neighbour*):ti,ab,kw near/3 (refer* or partner*):ti,ab,kw	1860
#20	#12 OR #13 OR #14 OR #15 OR #16 OR #17 OR #18 OR #19	548479
#21	"primary care":ti,ab,kw near/3 (access* or avail* or utiliz*or utilis*):ti,ab,kw	330
#22	"health services":ti,ab,kw near/3 (access* or avail* or utiliz*or utilis*):ti,ab,kw	1524
#23	healthcare:ti,ab,kw near/3 (access* or avail* or utiliz*or utilis*):ti,ab,kw	1149
#24	"health care":ti,ab,kw near/3 (access* or avail* or utiliz*or utilis*):ti,ab,kw	1763
#25	#21 OR #22 OR #23 OR #24	4371
#26	primary:ti,ab,kw next care:ti,ab,kw	21124
#27	comprehensive:ti,ab,kw next care:ti,ab,kw	367
#28	"primary health care":ti,ab,kw	8158
#29	"comprehensive health care":ti,ab,kw	112
#30	comprehensive:ti,ab,kw next healthcare:ti,ab,kw	18
#31	primary:ti,ab,kw next healthcare:ti,ab,kw	867
#32	(safety-net:ti,ab,kw or "safety net":ti,ab,kw) next clinic*:ti,ab,kw	91
#33	"community health center":ti,ab,kw	338
#34	"community health centers":ti,ab,kw	749
#35	"federally qualified health center":ti,ab,kw	169
#36	"federally qualified health centers":ti,ab,kw	160
#37	fqhc:ti,ab,kw	104
#38	(family or general or primary):ti,ab,kw near/2 (medicine or practice or practitioner* or physician* or doctor* or provider* or clinic* or clinician* or nurs*):ti,ab,kw	32151
#39	#26 OR #27 OR #28 OR #29 OR #30 OR #31 OR #32 OR #33 OR #34 OR #35 OR #36 OR #37 OR #38	45772
#40	#25 OR #39	48927
#41	#11 AND #20 AND #40	1387
#42	#20 AND #25	2907
#43	#41 OR #42 with Cochrane Library publication date in The last year, in Cochrane Reviews, Trials	414

© 2024 Viswanathan M et al. *JAMA Network Open.*

eTable 9. Ovid MEDLINE® Search String and Yield for Food Insecurity, Housing, Education and Literacy, Financial Strain, Employment, Transportation, Utilities, Social Isolation, Early Childhood Development, Legal Services, and Childcare (Ovid MEDLINE®) (February 7, 2023)

ID	Search	Hits
1	"Social Determinants of Health"/	6086
2	Social Conditions/	9607
3	Social Environment/	44391
4	Social Class/	44368
5	Socioeconomic Factors/	170898
6	(social* adj1 determin*).ti,ab,kf.	12879
7	((determinant* or determinate*) adj2 health).ti,ab,kf.	14378
8	((social* or socio*) adj1 condition*).ti,ab,kf.	7744
9	((social* or socio*) adj1 environment*).ti,ab,kf.	13299
10	((social* or socio*) adj1 (factor* or gradient*)).ti,ab,kf.	45226
11	((social* or socio*) adj1 (need* or require*)).ti,ab,kf.	3153
12	((social* or socio*) adj1 (equit* or inequit* or disparit* or equal* or inequal*)).ti,ab,kf.	11125
13	((social* or socio*) adj1 (hardship* or depriv* or challeng* or difficult* or barrier* or vulnerab* or disadvantag*)).ti,ab,kf.	16495
14	((social* or socio*) adj1 risk*).ti,ab,kf.	3391
15	((social* or socio*) adj1 (status* or circumstance* or position* or class*)).ti,ab,kf.	71052
16	Food Supply/	15422
17	Hunger/	5984
18	(food adj2 (secur* or insecur* or unstable or stable or stabilit* or instabilit* or uncertain* or vulnerab* or hardship* or insufficien* or stress*)).ti,ab,kf.	14575
19	food desert*.ti,ab,kf.	276
20	Housing/	19906
21	Almshouses/	53
22	Public Housing/	1593
23	((hous* or home) adj3 (secur* or insecur* or unstable or stable or stabilit* or instabilit* or uncertain* or vulnerab* or hardship* or insufficien* or stress*)).ti,ab,kf.	7121
24	Homeless Persons/	9421
25	Homeless Youth/	1417
26	(homeless* or houseless*).ti,ab,kf.	11972
27	Transportation/	11705
28	Transportation Facilities/	61
29	Parking Facilities/	364
30	transportation*.ti.	4270
31	commut*.ti,ab,kf.	4162
32	Educational Status/	57865
33	Academic Failure/	55
34	Literacy/	1496
35	Reading/	25344
36	(literacy or literate or illitera*).ti,ab,kf.	28967
37	(read* adj2 (proficien* or skill* or comprehension or level*)).ti,ab,kf.	8180
38	((education* or academic* or schola* or school*) adj2 (achieve* or status or attain* or equit* or inequit* or disparit* or equal* or inequalit* or level* or background*)).ti,ab,kf.	97096
39	((education* or academic* or schola* or school*) adj2 (opportunit* or disadvantage* or advantage* or marginal* or disenfranchis* or vulnerab*)).ti,ab,kf.	4908
40	Poverty/	43244
41	Poverty Areas/	6629
42	((economic* or income* or financ*) adj2 (achieve* or status or attain* or equit* or inequit* or disparit* or equal* or inequalit* or level* or background*)).ti,ab,kf.	39123
43	((economic* or income* or financ*) adj2 (opportunit* or disadvantage* or advantage* or marginal* or disenfranchis* or vulnerab* or low or strain* or strugg* or stable or unstable or stabilit* or instabilit* or difficult* or problem*)).ti,ab,kf.	62597
44	(poverty or indigent* or indigency or impoverish*).ti.	6012
45	Employment/	50005
46	Unemployment/	7736

© 2024 Viswanathan M et al. *JAMA Network Open.*

ID	Search	Hits
47	unemployment.ti,ab,kf.	11830
48	unemployed.ti,ab,kf.	9412
49	underemploy*.ti,ab,kf.	387
50	(occupation* adj2 (status or level or class)).ti,ab,kf.	7172
51	jobless*.ti,ab,kf.	295
52	workless*.ti,ab,kf.	33
53	(employment adj2 (status or securit* or insecurit* or marginal* or precarious* or terminat*)).ti,ab,kf.	10775
54	Child Care/	6061
55	(child adj2 care).ti,ab,kf.	10488
56	Social Isolation/	15992
57	(social* adj2 isolat*).ti,ab,kf.	10964
58	Legal Services/	44
59	(legal adj2 service*).ti,ab,kf.	735
60	((water or power or electric* or gas or sewer or sanit* or phone or internet or cable or satellite) adj3 (utility or utilities)).ti,ab,kf.	1435
61	Early Intervention, Educational/	3475
62	Child Development/	50039
63	Language Development/	11924
64	((child* or toddler or infant*) adj3 (educat* or develop*)).ti,ab,kf.	89962
65	1 or 2 or 3 or 4 or 5 or 6 or 7 or 8 or 9 or 10 or 11 or 12 or 13 or 14 or 15 or 16 or 17 or 18 or 19 or 20 or 21 or 22 or 23 or 24 or 25 or 26 or 27 or 28 or 29 or 30 or 31 or 32 or 33 or 34 or 35 or 36 or 37 or 38 or 39 or 40 or 41 or 42 or 43 or 44 or 45 or 46 or 47 or 48 or 49 or 50 or 51 or 52 or 53	685665
66	54 or 55 or 56 or 57 or 58 or 59 or 60 or 61 or 62 or 63 or 64	172833
67	Mass Screening/	115493
68	(Surveys and Questionnaires).mp. [mp=title, book title, abstract, original title, name of substance word, subject heading word, floating sub-heading word, keyword heading word, organism supplementary concept word, protocol supplementary concept word, rare disease supplementary concept word, unique identifier, synonyms]	559608
69	screen*.ti,ab,kf.	806012
70	(instrument* or tool*).ti.	129542
71	67 or 68 or 69 or 70	1465173
72	Needs Assessment/	32327
73	Program Development/	30260
74	(Referral and Consultation).mp. [mp=title, book title, abstract, original title, name of substance word, subject heading word, floating sub-heading word, keyword heading word, organism supplementary concept word, protocol supplementary concept word, rare disease supplementary concept word, unique identifier, synonyms]	77439
75	Pilot Projects/	145105
76	Social Welfare/	9721
77	Food Assistance/	1747
78	Public Assistance/	3010
79	Patient Navigation/	1026
80	Patient Advocacy/	24193
81	Inservice Training/	20737
82	Staff Development/	9851
83	intervention*.ti,ab,kf.	1090783
84	(need* adj2 (assessment* or evaluat* or determin*)).ti,ab,kf.	67605
85	(food adj2 (assist* or aid or help*)).ti,ab,kf.	1615
86	((hous* or home) adj2 (assist* or aid or help*)).ti,ab,kf.	2783
87	(transportation adj2 (assist* or aid or help*)).ti,ab,kf.	256
88	((education* or academic* or schola* or school*) adj2 (assist* or aid or help*)).ti,ab,kf.	4846
89	((employment or occupation* or job*) adj2 (assist* or aid or help*)).ti,ab,kf.	1143
90	((economic* or income* or financ*) adj2 (assist* or aid or help*)).ti,ab,kf.	2880
91	patient navigat*.ti,ab,kf.	1293
92	patient advoca*.ti,ab,kf.	2807
93	((staff or employee*) adj2 (develop* or train* or educat* or curricul*)).ti,ab,kf.	16607

ID	Search	Hits
94	((social* or socio* or communit* or neighbor* or neighbour*) adj3 (refer* or partner*)).ti,ab,kf.	14034
95	((utility or utilities) adj2 (assist* or help or aid)).ti,ab,kf.	157
96	(legal adj2 (assist* or help or aid)).ti,ab,kf.	434
97	72 or 73 or 74 or 75 or 76 or 77 or 78 or 79 or 80 or 81 or 82 or 83 or 84 or 85 or 86 or 87 or 88 or 89 or 90 or 91 or 92 or 93 or 94	1467631
98	71 or 97	2739200
99	95 or 96	590
100	Primary Health Care/	90302
101	Comprehensive Health Care/	6776
102	General Practice/	15223
103	General Practitioners/	10279
104	Family Practice/	66843
105	Physicians, Family/	17134
106	Physicians, Primary Care/	4303
107	Primary Care Nursing/	564
108	Nurse Practitioners/	18761
109	Family Nurse Practitioners/	75
110	Pediatric Nurse Practitioners/	189
111	Physician Assistants/	6271
112	Family Nursing/	1565
113	Community Health Nursing/	19759
114	Community Health Centers/	7500
115	Community Mental Health Centers/	3029
116	Community Health Services/	32991
117	Community Mental Health Services/	19012
118	Community Health Workers/	6477
119	Safety-net Providers/	1305
120	primary care.ti,ab,kf.	124201
121	primary health care.ti,ab,kf.	28774
122	((family or general or primary) adj1 (medicine or practice or practitioner* or physician* or doctor* or provider* or clinic* or clinician*)).ti,ab,kf.	129590
123	Patient-Centered Care/	22624
124	Patient Care Team/	69239
125	Health Services/	27365
126	"Delivery of Health Care"/	113839
127	Emergency Medicine/	15055
128	Pediatric Emergency Medicine/	484
129	exp emergency medical services/	166306
130	(emergency adj2 (medicine or servic* or room* or department* or physician* or doctor* or provider* or clinician*)).ti,ab,kf.	149686
131	100 or 101 or 102 or 103 or 104 or 105 or 106 or 107 or 108 or 109 or 110 or 111 or 112 or 113 or 114 or 115 or 116 or 117 or 118 or 119 or 120 or 121 or 122 or 123 or 124 or 125 or 126 or 127 or 128 or 129 or 130	808318
132	65 and 98 and 131	26382
133	132 and "Case Reports".sa_pubt.	173
134	132 not 133	26209
135	limit 134 to english language	24762
136	limit 135 to yr="2020 -Current"	4511
137	98 or 99	2739581
138	66 and 131 and 137	4007
139	limit 138 to case reports	100
140	138 not 139	3907
141	limit 140 to english language	3679
142	limit 141 to yr="2020 -Current"	613
143	136 or 142	4946
144	limit 143 to last year	1734

© 2024 Viswanathan M et al. *JAMA Network Open.*

eTable 10. Cochrane Library (Including Both Cochrane Database of Systematic Reviews and Cochrane Central Register of Controlled Trials) Search String and Yield for Food Insecurity, Housing, Education and Literacy, Financial Strain, Employment, Transportation, Utilities, Social Isolation, Early Childhood Development, Legal Services, and Childcare (February 1, 2023)

ID	Search	Hits
#1	social*:ti,ab,kw near/1 determin*:ti,ab,kw	576
#2	(determinant* or determinate*):ti,ab,kw near/2 health:ti,ab,kw	612
#3	(social* or socio*):ti,ab,kw near/1 condition*:ti,ab,kw	473
#4	(social* or socio*):ti,ab,kw near/1 environment*:ti,ab,kw	1827
#5	(social* or socio*):ti,ab,kw near/1 (factor* or gradient*):ti,ab,kw	6230
#6	(social* or socio*):ti,ab,kw near/1 (need* or require*):ti,ab,kw	316
#7	(social* or socio*):ti,ab,kw near/1 (equit* or inequit* or disparit* or equal* or inequal*):ti,ab,kw	255
#8	(social* or socio*):ti,ab,kw near/1 (hardship* or depriv* or challeng* or difficult* or barrier* or vulnerab* or disadvantag*):ti,ab,kw	1448
#9	(social* or socio*):ti,ab,kw near/1 risk*:ti,ab,kw	301
#10	(social* or socio*):ti,ab,kw near/1 (status* or circumstance* or position* or class* or standing):ti,ab,kw	6158
#11	food*:ti,ab,kw near/2 (supply or secur* or insecur* or unstable or stable or stabilit* or instabilit* or uncertain* or vulnerab* or hardship* or insufficien* or stress*):ti,ab,kw	1264
#12	food:ti,ab,kw next desert*:ti,ab,kw	12
#13	(hous* or home):ti,ab,kw near/3 (secur* or insecur* or unstable or stable or stabilit* or instabilit* or uncertain* or vulnerab* or hardship* or insufficien* or stress*):ti,ab,kw	882
#14	(homeless* or houseless*):ti,ab,kw	1108
#15	Transportation*:ti,ab,kw	2027
#16	commut*:ti,ab,kw	266
#17	(literacy or literate or illitera*):ti,ab,kw	6677
#18	read*:ti,ab,kw near/2 (proficien* or skill* or comprehension or level*):ti,ab,kw	1100
#19	(education* or academic* or schola* or school*):ti,ab,kw near/2 (achieve* or fail* or status or attain* or equit* or inequit* or disparit* or equal* or inequalit* or level* or background*):ti,ab,kw	12186
#20	(education* or academic* or schola* or school*):ti,ab,kw near/2 (opportunit* or disadvantage* or advantage* or marginal* or disenfranchis* or vulnerab*):ti,ab,kw	293
#21	(economic* or income* or financ*):ti,ab,kw near/2 (achieve* or status or attain* or equit* or inequit* or disparit* or equal* or inequalit* or level* or background*):ti,ab,kw	2546
#22	(economic* or income* or financ*):ti,ab,kw near/2 (opportunit* or disadvantage* or advantage* or marginal* or disenfranchis* or vulnerab* or low or strain* or strugg* or stable or unstable or stabilit* or instabilit* or difficult* or problem* or stress*):ti,ab,kw	7302
#23	(poverty or indigent* or indigency or impoverish*):ti,ab,kw	3725
#24	unemployment:ti,ab,kw	916
#25	unemployed:ti,ab,kw	710
#26	underemployed:ti,ab,kw	10
#27	(occupation* or job):ti,ab,kw near/2 (status or level or class):ti,ab,kw	537
#28	jobless*:ti,ab,kw	5
#29	workless*:ti,ab,kw	2
#30	(employment or job or occupation*):ti,ab,kw near/2 (status or securit* or insecurit* or marginal* or precarious* or terminat*):ti,ab,kw	1628
#31	child:ti,ab,kw near/2 care:ti,ab,kw	4374
#32	social*:ti,ab,kw near/2 isolat*:ti,ab,kw	1294
#33	legal:ti,ab,kw near/2 service*:ti,ab,kw	42
#34	(water or power or electric* or gas or sewer or sanit* or phone or internet or cable or satellite):ti,ab,kw near/3 (utility or utilities):ti,ab,kw	65
#35	(child* or toddler or infant*):ti,ab,kw near/3 (educat* or develop* or language*):ti,ab,kw	14449
#36	#5 OR #6 OR #7 OR #8 OR #9 OR #10 OR #11 OR #12 OR #13 OR #14 OR #15 OR #16 OR #17 OR #18 OR #19 OR #20 OR #21 OR #22 OR #23 OR #24 OR #25 OR #26 OR #27 OR #28 OR #29 OR #30	44101
#37	#31 OR #32 OR #33 OR #34 OR #35	19417
#38	screen*:ti,ab,kw	91136

© 2024 Viswanathan M et al. *JAMA Network Open.*

ID	Search	Hits
#39	(instrument* or tool*):ti	7111
#40	#38 OR #39	97482
#41	intervention*:ti,ab,kw	522797
#42	need*:ti,ab,kw near/2 (assessment* or evaluat* or determin*):ti,ab,kw	10181
#43	program*:ti,ab,kw near/2 develop*:ti,ab,kw	4863
#44	pilot:ti,ab,kw next project*:ti,ab,kw	26017
#45	food:ti,ab,kw near/2 (assist* or aid or help*):ti,ab,kw	342
#46	(hous* or home):ti,ab,kw near/2 (assist* or aid or help*):ti,ab,kw	468
#47	transportation*:ti,ab,kw near/2 (assist* or aid or help*):ti,ab,kw	36
#48	(education* or academic* or schola* or school*):ti,ab,kw near/2 (assist* or aid or help*):ti,ab,kw	925
#49	(employment or occupation* or job*):ti,ab,kw near/2 (assist* or aid or help*):ti,ab,kw	110
#50	(economic* or income* or financ*):ti,ab,kw near/2 (assist* or aid or help*):ti,ab,kw	374
#51	patient*:ti,ab,kw near/1 navigat*:ti,ab,kw	742
#52	patient*:ti,ab,kw near/2 advoca*:ti,ab,kw	440
#53	(staff or employee*):ti,ab,kw near/2 (develop* or train* or educat* or curricul*):ti,ab,kw	3114
#54	(social* or socio* or communit* or neighbor* or neighbour*):ti,ab,kw near/3 (refer* or partner*):ti,ab,kw	1860
#55	(utility or utilities):ti,ab,kw near/2 (assist* or help or aid):ti,ab,kw	13
#56	legal:ti,ab,kw near/2 (assist* or help or aid):ti,ab,kw	26
#57	#41 OR #42 OR #43 OR #44 OR #45 OR #46 OR #47 OR #48 OR #49 OR #50 OR #51 OR #52 OR #53 OR #54 OR #55 OR #56	549239
#58	#40 OR #57	599558
#59	#36 AND #58	28841
#60	#37 AND #58	11474
#61	primary:ti,ab,kw next care:ti,ab,kw	21124
#62	comprehensive:ti,ab,kw next care:ti,ab,kw	367
#63	"primary health care":ti,ab,kw	8158
#64	"comprehensive health care":ti,ab,kw	112
#65	comprehensive:ti,ab,kw next healthcare:ti,ab,kw	18
#66	primary:ti,ab,kw next healthcare:ti,ab,kw	867
#67	(safety-net:ti,ab,kw or "safety net":ti,ab,kw) next clinic*:ti,ab,kw	91
#68	"community health center":ti,ab,kw	338
#69	"community health centers":ti,ab,kw	749
#70	"federally qualified health center":ti,ab,kw	169
#71	"federally qualified health centers":ti,ab,kw	160
#72	fqhc:ti,ab,kw	104
#73	(family or general or primary):ti,ab,kw near/2 (medicine or practice or practitioner* or physician* or doctor* or provider* or clinic* or clinician* or nurs*):ti,ab,kw	32151
#74	emergency:ti,ab,kw near/2 (medicine or servic* or room* or department* or physician* or doctor* or provider* or clinician*):ti,ab,kw	19601
#75	#61 OR #62 OR #63 OR #64 OR #65 OR #66 OR #67 OR #68 OR #69 OR #70 OR #71 OR #72 OR #73 OR #74	63545
#76	#59 AND #75	3950
#77	#60 AND #75	1099
#78	#76 OR #77 with Cochrane Library publication date in The last year, in Cochrane Reviews, Trials	457

eTable 11. Ovid MEDLINE® Search String and Yield for Interpersonal Violence MEDLINE Search (Ovid MEDLINE®) (February 7, 2023)

ID	Search	Hits
1	"Social Determinants of Health"/	6086
2	Social Conditions/	9607
3	Social Environment/	44391
4	Social Class/	44368
5	Socioeconomic Factors/	170898
6	(social* adj1 determin*).ti,ab,kf.	12879
7	((determinant* or determinate*) adj2 health).ti,ab,kf.	14378

© 2024 Viswanathan M et al. *JAMA Network Open.*

ID	Search	Hits
8	((social* or socio*) adj1 condition*).ti,ab,kf.	7744
9	((social* or socio*) adj1 environment*).ti,ab,kf.	13299
10	((social* or socio*) adj1 (factor* or gradient*)).ti,ab,kf.	45226
11	((social* or socio*) adj1 (need* or require*)).ti,ab,kf.	3153
12	((social* or socio*) adj1 (equit* or inequit* or disparit* or equal* or inequal*)).ti,ab,kf.	11125
13	((social* or socio*) adj1 (hardship* or depriv* or challeng* or difficult* or barrier* or vulnerab* or disadvantag*)).ti,ab,kf.	16495
14	((social* or socio*) adj1 risk*).ti,ab,kf.	3391
15	((social* or socio*) adj1 (status* or circumstance* or position* or class*)).ti,ab,kf.	71052
16	Food Supply/	15422
17	Hunger/	5984
18	(food adj2 (secur* or insecur* or unstable or stable or stabilit* or instabilit* or uncertain* or vulnerab* or hardship* or insufficien* or stress*)).ti,ab,kf.	14575
19	food desert*.ti,ab,kf.	276
20	Housing/	19906
21	Almshouses/	53
22	Public Housing/	1593
23	((hous* or home) adj3 (secur* or insecur* or unstable or stable or stabilit* or instabilit* or uncertain* or vulnerab* or hardship* or insufficien* or stress*)).ti,ab,kf.	7121
24	Homeless Persons/	9421
25	Homeless Youth/	1417
26	(homeless* or houseless*).ti,ab,kf.	11972
27	Transportation/	11705
28	Transportation Facilities/	61
29	Parking Facilities/	364
30	transportation*.ti.	4270
31	commut*.ti,ab,kf.	4162
32	Educational Status/	57865
33	Academic Failure/	55
34	Literacy/	1496
35	Reading/	25344
36	(literacy or literate or illitera*).ti,ab,kf.	28967
37	(read* adj2 (proficien* or skill* or comprehension or level*)).ti,ab,kf.	8180
38	((education* or academic* or schola* or school*) adj2 (achieve* or status or attain* or equit* or inequit* or disparit* or equal* or inequalit* or level* or background*)).ti,ab,kf.	97096
39	((education* or academic* or schola* or school*) adj2 (opportunit* or disadvantage* or advantage* or marginal* or disenfranchis* or vulnerab*)).ti,ab,kf.	4908
40	Poverty/	43244
41	Poverty Areas/	6629
42	((economic* or income* or financ*) adj2 (achieve* or status or attain* or equit* or inequit* or disparit* or equal* or inequalit* or level* or background*)).ti,ab,kf.	39123
43	((economic* or income* or financ*) adj2 (opportunit* or disadvantage* or advantage* or marginal* or disenfranchis* or vulnerab* or low or strain* or strugg* or stable or unstable or stabilit* or instabilit* or difficult* or problem*)).ti,ab,kf.	62597
44	(poverty or indigent* or indigency or impoverish*).ti.	6012
45	Employment/	50005
46	Unemployment/	7736
47	unemployment.ti,ab,kf.	11830
48	unemployed.ti,ab,kf.	9412
49	underemploy*.ti,ab,kf.	387
50	(occupation* adj2 (status or level or class)).ti,ab,kf.	7172
51	jobless*.ti,ab,kf.	295
52	workless*.ti,ab,kf.	33
53	(employment adj2 (status or securit* or insecurit* or marginal* or precarious* or terminat*)).ti,ab,kf.	10775
54	Child Care/	6061
55	(child adj2 care).ti,ab,kf.	10488
56	Social Isolation/	15992
57	(social* adj2 isolat*).ti,ab,kf.	10964

© 2024 Viswanathan M et al. *JAMA Network Open.*

ID	Search	Hits
58	Legal Services/	44
59	(legal adj2 service*).ti,ab,kf.	735
60	((water or power or electric* or gas or sewer or sanit* or phone or internet or cable or satellite) adj3 (utility or utilities)).ti,ab,kf.	1435
61	Early Intervention, Educational/	3475
62	Child Development/	50039
63	Language Development/	11924
64	((child* or toddler or infant*) adj3 (educat* or develop*)).ti,ab,kf.	89962
65	1 or 2 or 3 or 4 or 5 or 6 or 7 or 8 or 9 or 10 or 11 or 12 or 13 or 14 or 15 or 16 or 17 or 18 or 19 or 20 or 21 or 22 or 23 or 24 or 25 or 26 or 27 or 28 or 29 or 30 or 31 or 32 or 33 or 34 or 35 or 36 or 37 or 38 or 39 or 40 or 41 or 42 or 43 or 44 or 45 or 46 or 47 or 48 or 49 or 50 or 51 or 52 or 53	685665
66	54 or 55 or 56 or 57 or 58 or 59 or 60 or 61 or 62 or 63 or 64	172833
67	Mass Screening/	115493
68	(Surveys and Questionnaires).mp. [mp=title, book title, abstract, original title, name of substance word, subject heading word, floating sub-heading word, keyword heading word, organism supplementary concept word, protocol supplementary concept word, rare disease supplementary concept word, unique identifier, synonyms]	559608
69	screen*.ti,ab,kf.	806012
70	(instrument* or tool*).ti.	129542
71	67 or 68 or 69 or 70	1465173
72	Needs Assessment/	32327
73	Program Development/	30260
74	(Referral and Consultation).mp. [mp=title, book title, abstract, original title, name of substance word, subject heading word, floating sub-heading word, keyword heading word, organism supplementary concept word, protocol supplementary concept word, rare disease supplementary concept word, unique identifier, synonyms]	77439
75	Pilot Projects/	145105
76	Social Welfare/	9721
77	Food Assistance/	1747
78	Public Assistance/	3010
79	Patient Navigation/	1026
80	Patient Advocacy/	24193
81	Inservice Training/	20737
82	Staff Development/	9851
83	intervention*.ti,ab,kf.	1090783
84	(need* adj2 (assessment* or evaluat* or determin*)).ti,ab,kf.	67605
85	(food adj2 (assist* or aid or help*)).ti,ab,kf.	1615
86	((hous* or home) adj2 (assist* or aid or help*)).ti,ab,kf.	2783
87	(transportation adj2 (assist* or aid or help*)).ti,ab,kf.	256
88	((education* or academic* or schola* or school*) adj2 (assist* or aid or help*)).ti,ab,kf.	4846
89	((employment or occupation* or job*) adj2 (assist* or aid or help*)).ti,ab,kf.	1143
90	((economic* or income* or financ*) adj2 (assist* or aid or help*)).ti,ab,kf.	2880
91	patient navigat*.ti,ab,kf.	1293
92	patient advoca*.ti,ab,kf.	2807
93	((staff or employee*) adj2 (develop* or train* or educat* or curricul*)).ti,ab,kf.	16607
94	((social* or socio* or communit* or neighbor* or neighbour*) adj3 (refer* or partner*)).ti,ab,kf.	14034
95	((utility or utilities) adj2 (assist* or help or aid)).ti,ab,kf.	157
96	(legal adj2 (assist* or help or aid)).ti,ab,kf.	434
97	72 or 73 or 74 or 75 or 76 or 77 or 78 or 79 or 80 or 81 or 82 or 83 or 84 or 85 or 86 or 87 or 88 or 89 or 90 or 91 or 92 or 93 or 94	1467631
98	71 or 97	2739200
99	95 or 96	590
100	Primary Health Care/	90302
101	Comprehensive Health Care/	6776
102	General Practice/	15223
103	General Practitioners/	10279
104	Family Practice/	66843

ID	Search	Hits
105	Physicians, Family/	17134
106	Physicians, Primary Care/	4303
107	Primary Care Nursing/	564
108	Nurse Practitioners/	18761
109	Family Nurse Practitioners/	75
110	Pediatric Nurse Practitioners/	189
111	Physician Assistants/	6271
112	Family Nursing/	1565
113	Community Health Nursing/	19759
114	Community Health Centers/	7500
115	Community Mental Health Centers/	3029
116	Community Health Services/	32991
117	Community Mental Health Services/	19012
118	Community Health Workers/	6477
119	Safety-net Providers/	1305
120	primary care.ti,ab,kf.	124201
121	primary health care.ti,ab,kf.	28774
122	((family or general or primary) adj1 (medicine or practice or practitioner* or physician* or doctor* or provider* or clinic* or clinician*)).ti,ab,kf.	129590
123	Patient-Centered Care/	22624
124	Patient Care Team/	69239
125	Health Services/	27365
126	"Delivery of Health Care"/	113839
127	Emergency Medicine/	15055
128	Pediatric Emergency Medicine/	484
129	exp emergency medical services/	166306
130	(emergency adj2 (medicine or servic* or room* or department* or physician* or doctor* or provider* or clinician*)).ti,ab,kf.	149686
131	100 or 101 or 102 or 103 or 104 or 105 or 106 or 107 or 108 or 109 or 110 or 111 or 112 or 113 or 114 or 115 or 116 or 117 or 118 or 119 or 120 or 121 or 122 or 123 or 124 or 125 or 126 or 127 or 128 or 129 or 130	808318
132	65 and 98 and 131	26382
133	132 and "Case Reports".sa_pubt.	173
134	132 not 133	26209
135	limit 134 to english language	24762
136	limit 135 to yr="2020 -Current"	4511
137	98 or 99	2739581
138	66 and 131 and 137	4007
139	limit 138 to case reports	100
140	138 not 139	3907
141	limit 140 to english language	3679
142	limit 141 to yr="2020 -Current"	613
143	136 or 142	4946
144	limit 143 to last year	1734
145	Physical Abuse/	1107
146	Gun Violence/	429
147	gender based violence.mp. [mp=title, book title, abstract, original title, name of substance word, subject heading word, floating sub-heading word, keyword heading word, organism supplementary concept word, protocol supplementary concept word, rare disease supplementary concept word, unique identifier, synonyms]	1491
148	Elder Abuse.mp. [mp=title, book title, abstract, original title, name of substance word, subject heading word, floating sub-heading word, keyword heading word, organism supplementary concept word, protocol supplementary concept word, rare disease supplementary concept word, unique identifier, synonyms]	3069
149	rape.mp. [mp=title, book title, abstract, original title, name of substance word, subject heading word, floating sub-heading word, keyword heading word, organism supplementary concept word, protocol supplementary concept word, rare disease supplementary concept word, unique identifier, synonyms]	11275

© 2024 Viswanathan M et al. *JAMA Network Open.*

ID	Search	Hits
150	workplace violence.mp. [mp=title, book title, abstract, original title, name of substance word, subject heading word, floating sub-heading word, keyword heading word, organism supplementary concept word, protocol supplementary concept word, rare disease supplementary concept word, unique identifier, synonyms]	2155
151	torture.mp. [mp=title, book title, abstract, original title, name of substance word, subject heading word, floating sub-heading word, keyword heading word, organism supplementary concept word, protocol supplementary concept word, rare disease supplementary concept word, unique identifier, synonyms]	2866
152	((elder* or geriatric* or aged or interpersonal or gun* or workplace) adj2 (violen* or abus* or neglect* or maltreat* or batter*)).ti.	3241
153	145 or 146 or 147 or 148 or 149 or 150 or 151 or 152	22821
154	Mass Screening.mp. [mp=title, book title, abstract, original title, name of substance word, subject heading word, floating sub-heading word, keyword heading word, organism supplementary concept word, protocol supplementary concept word, rare disease supplementary concept word, unique identifier, synonyms]	117931
155	Anonymous Testing.mp. [mp=title, book title, abstract, original title, name of substance word, subject heading word, floating sub-heading word, keyword heading word, organism supplementary concept word, protocol supplementary concept word, rare disease supplementary concept word, unique identifier, synonyms]	545
156	Mass Chest X-Ray.mp. [mp=title, book title, abstract, original title, name of substance word, subject heading word, floating sub-heading word, keyword heading word, organism supplementary concept word, protocol supplementary concept word, rare disease supplementary concept word, unique identifier, synonyms]	1960
157	Multiphasic Screening.mp. [mp=title, book title, abstract, original title, name of substance word, subject heading word, floating sub-heading word, keyword heading word, organism supplementary concept word, protocol supplementary concept word, rare disease supplementary concept word, unique identifier, synonyms]	1158
158	risk.mp. [mp=title, book title, abstract, original title, name of substance word, subject heading word, floating sub-heading word, keyword heading word, organism supplementary concept word, protocol supplementary concept word, rare disease supplementary concept word, unique identifier, synonyms]	2766712
159	logistic models.mp. [mp=title, book title, abstract, original title, name of substance word, subject heading word, floating sub-heading word, keyword heading word, organism supplementary concept word, protocol supplementary concept word, rare disease supplementary concept word, unique identifier, synonyms]	153762
160	Protective Factors.mp. [mp=title, book title, abstract, original title, name of substance word, subject heading word, floating sub-heading word, keyword heading word, organism supplementary concept word, protocol supplementary concept word, rare disease supplementary concept word, unique identifier, synonyms]	16924
161	Risk Assessment.mp. [mp=title, book title, abstract, original title, name of substance word, subject heading word, floating sub-heading word, keyword heading word, organism supplementary concept word, protocol supplementary concept word, rare disease supplementary concept word, unique identifier, synonyms]	339725
162	Adverse Outcome Pathways.mp. [mp=title, book title, abstract, original title, name of substance word, subject heading word, floating sub-heading word, keyword heading word, organism supplementary concept word, protocol supplementary concept word, rare disease supplementary concept word, unique identifier, synonyms]	635
163	"Healthcare Failure Mode and Effect Analysis".mp. [mp=title, book title, abstract, original title, name of substance word, subject heading word, floating sub-heading word, keyword heading word, organism supplementary concept word, protocol supplementary concept word, rare disease supplementary concept word, unique identifier, synonyms]	214
164	Risk Factors.mp. [mp=title, book title, abstract, original title, name of substance word, subject heading word, floating sub-heading word, keyword heading word, organism supplementary concept word, protocol supplementary concept word, rare disease supplementary concept word, unique identifier, synonyms]	1147393
165	(screen* or risk).ti.	671242
166	154 or 155 or 156 or 157 or 158 or 159 or 160 or 161 or 162 or 163 or 164 or 165	3013318
167	153 and 166	5457
168	exp "Surveys and Questionnaires"/	1197464

© 2024 Viswanathan M et al. *JAMA Network Open.*

ID	Search	Hits
169	exp Diagnosis/	9267695
170	interven*.ti.	167071
171	168 or 169 or 170	10080952
172	153 and 171	5180
173	167 or 172	8820
174	limit 173 to english language	8326
175	limit 174 to yr="2020 -Current"	1727
176	limit 175 to last year	620

eTable 12. Cochrane Library (Including Both Cochrane Database of Systematic Reviews and Cochrane Central Register of Controlled Trials) Search String and Yield for Interpersonal Violence (February 1, 2023)

ID	Search	Hits
#1	physical abuse	2635
#2	gun violence	45
#3	gender based violence	527
#4	elder abuse	1050
#5	rape	450
#6	workplace violence	104
#7	torture	89
#8	((elder* or geriatric* or aged or interpersonal or gun* or workplace) NEAR/2 (violen* or abus* or neglect* or maltreat* or batter*)):ti	117
#9	#1 OR #2 OR #3 OR #4 OR #5 OR #6 OR #7 OR #8	4127
#10	Mass Screening	11478
#11	Anonymous Testing	368
#12	Mass Chest X-Ray	609
#13	Multiphasic Screening	61
#14	risk	282919
#15	logistic models	11727
#16	Risk Assessment	84890
#17	Adverse Outcome Pathways	1847
#18	"Healthcare Failure Mode and Effect Analysis"	4
#19	Risk Factors	95218
#20	#10 OR #11 OR #12 OR #13 OR #14 OR #15 OR #16 OR #17 OR #18 OR #19	296836
#21	#9 AND #20	1973
#22	MeSH descriptor: [Surveys and Questionnaires] explode all trees	68241
#23	MeSH descriptor: [Diagnosis] explode all trees	402241
#24	interven*:ti	74563
#25	#22 OR #23 OR #24	488304
#26	#9 AND #25	1230
#27	#26 OR #21 with Cochrane Library publication date in The last year, in Cochrane Reviews, Trials	168

eTable 13. Inclusion and Exclusion Criteria

Category	Inclusion	Exclusion
Populations	Demographic characteristics: general population, all ages, immigrants, racial and ethnic minority groups<br>Health status: pregnant women; studies targeting people with asthma, heart disease, diabetes, hypertension, mental health conditions, or substance abuse disorders; persons with multiple chronic conditions	Demographic characteristics: no exclusion criteria<br>Health status: studies targeting people with specific diseases other than asthma, heart disease, diabetes, hypertension, mental health conditions, or substance abuse disorders
Social needs	Interventions addressing individual social needs Food insecurity, housing instability and quality, interpersonal violence (with the exclusion of intimate partner violence and child maltreatment[a]), education (including adult literacy and health literacy), financial strain, employment, social isolation, early childhood education and development, healthcare and primary care, transportation, utilities, legal services, and childcare<br>Interventions targeting single or multiple domains; can address excluded domains if 1 of the included domains above is addressed	Social needs addressed by US Preventive Services Task Force recommendations (depression, alcohol abuse, healthy diet, drug use, physical activity, tobacco use, intimate partner violence, and child maltreatment) or Centers for Disease Control and Prevention (neighborhood and built environment)<br>Other social needs not included in Healthy People 2020
Interventions	Individual level (eg, referral to social services, provision of information about resources)<br>Healthcare system level (eg, policies, programs, staff training, primary care collaboration with community services)<br>Adjustment interventions or assistance interventions	Public health and community-level policies<br>Individual-level interventions that target medical conditions and needs alone (rather than social needs alone or social needs in combination with medical needs)<br>Advocacy, alignment, or awareness interventions
Comparisons	Usual care	No comparator, comparative effectiveness
Outcomes	Behavioral outcomes, health outcomes, healthcare utilization outcomes, harms, and unanticipated outcomes	Process outcomes, social needs outcomes, cost outcomes, provider outcomes
Timing	All	None
Setting	Any setting linked with the healthcare system; conducted in the United States	Conducted outside the United States; no link with US healthcare system
Study design	Randomized controlled trials	Nonrandomized controlled trials, cohort studies, case-control studies (cases and controls defined by presence or absence of outcome), single-arm studies with data collected before and after the intervention (preintervention-postintervention)<br>Studies of head-to-head comparisons (ie, comparative effectiveness studies)<br>Case series, case reports, dissertations, modeling studies, screening tool validation studies, studies with a comparison group defined by the absence of social needs
Language	English	Non-English

[a] We excluded child maltreatment and intimate partner violence from interpersonal violence because these topics were covered by the US Preventive Services Task Force.

© 2024 Viswanathan M et al. *JAMA Network Open.*

eMethods. Identification of Social Needs

For this review of a scoping review, we also extracted information on how social needs were identified. In doing so, we used the PROGRESS-Plus framework[76] to categorize how social risk was defined and PRISMA-Equity to understand whether and how studies considered equity.[77] Specifically, we extracted information on whether and how social risk was defined (which we defined as the circumstances or context from which a social need might arise, not the social need itself [eg, low income rather than food insecurity]) , either at the individual or the population level, whether that definition of social risk was applied in study inclusion criteria, whether the participants were screened for or selected based on their social needs, whether the trial was developed in response to community need, and whether equity was stated or implied to be a goal.

© 2024 Viswanathan M et al. *JAMA Network Open.*

eFigure 1. Screening Approach

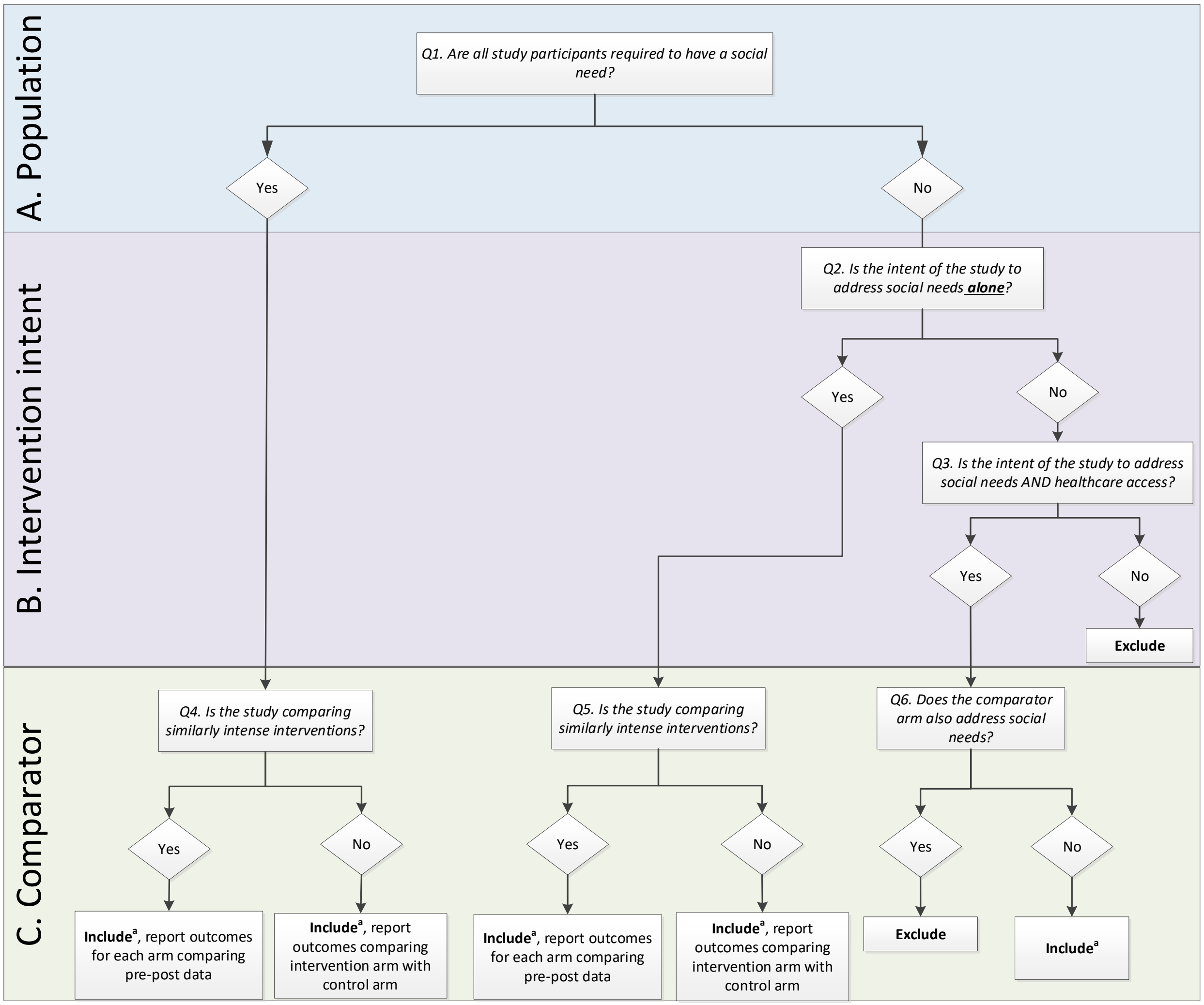

[a]if study meets all other eligibility criteria.

© 2024 Viswanathan M et al. *JAMA Network Open.*

eResults. Identification of Social Needs

1. **How are the social needs of the target population identified?**

We sought to understand the ways in which the design of social needs interventions addresses the specific needs of their intended recipients. Specifically, we sought to understand how studies (1) defined social risk, (2) screened participants or selected participants for social needs, (3) engaged community members in designing interventions, and (4) explicitly addressed equity as the rationale for the study. Overall, the findings indicate the majority of social needs RCTs define social risk and screen or select individuals or populations with needs (or proxies for needs). They are generally not explicit about whether the intervention was designed in response to a specific community need.

Specifically, the majority of RCTs explicitly defined the nature of the social risk they were seeking to address (64%; N = 49/77) (Table 2).[1,3,5,7-11,13-15,17,19-26,29,31-34,38,41,44,45,47,49,51,53-55,57-59,61,63-68,70,78-80] Among the studies that defined social risk, the majority defined it at the individual level (65%; N = 32/49).[1,5,7,9,11,14,15,17,19,22,23,25,26,31,38,41,44,45,47,51,53-55,57,59,61,64,66,67,78-80] Most studies (74%; N = 56/77) specified one or more PROGRESS-Plus features as inclusion criteria.[1,3,5-7,9-15,17,20,21,23-26,28,29,31-42,44,46,47,49-51,53,54,57,59-67,70,71,80-82] Among these studies, socioeconomic status was the most common PROGRESS criterion (70%; N = 39/56),[1,5,7,9,11,13-15,20,23-26,28,32,33,35-42,44,47,49,51,53,54,57,60,61,63,66,67,71,80,81] followed by time-dependent relationships such as transition of care from a healthcare setting or in relation to childbirth (32%; N = 18/56),[6,10,15,17,21,28,32,35,38,46,49,50,57,59,61,62,66,82] place of residence (21%; N = 12/56),[3,10,20,21,26,29,32-34,38,46,50,65,70,81,82] and personal characteristics associated with discrimination such as age (eg, adolescents or older adults) or disability (eg, blindness, specific chronic conditions) (18%; N = 10/56).[9,10,12-14,21,37,50,63,71]

Regarding screening or the selection of participants for social needs, the majority of studies attempted to assess needs through screening (44%; N = 34/77)[1,5,9-13,15,20,24,29,30,36-40,42,44,47,52-54,56,57,59,61-63,66,67,78,80,82] or assessment (12%; N = 9/77).[26,28,43,45,46,48,50,55,69] More than a third of studies bypassed the step of individual screening or assessment and instead drew study samples from populations with a known social need or a proxy for the social need, such as living

© 2024 Viswanathan M et al. *JAMA Network Open.*

in a low-income area or using safety-net healthcare (36%; N = 28/77).[2,3,6-8,14,16,21-23,25,31-35,41,49,51,58,60,64,65,68,70-72,81] A minority offered no justification or explanation for how participants were identified or selected (8%; N = 6/77).[4,17-19,27,79]

Regarding community engagement in supporting the rationale for the study, only 22% (N = 17/77) of studies reported input from the community.[8,11,13,17,19,21,26,32-35,38,41,57,68,71,82] Another 22 (29%) studies cited prevalence of the social need in the community as the rationale for the trial.[3,12-14,20,21,25,32,34,47,49,51,54,62,65-67,70,72,80-82] More than half the trials (60%; N = 46/77) either did not describe how the intervention was developed or cited prior interventions or literature as the rationale.[1,2,4-7,9,10,15-18,22-24,27-31,36,37,39,40,42-46,48,50,52,53,55,56,58-61,63,64,69,78,79,81]

Equity was not listed as an explicit or implicit rationale for most studies (62%; N = 48/77).[2,4-7,9,11,12,14-16,18,20,21,26-28,30-32,35,37,39,40,43,45,47-50,52-59,61,62,66,67,71,72,78,79,81,82] Only 6 of 77 studies defined social risk explicitly, reported that the intervention was developed in response to community need, and listed equity as the rationale.[3,13,34,64,68,70]

eTable 14. Risk of Bias Domains and Ratings

Author Year	Domain 1--Risk of bias arising from the randomization process	Domain 2--Risk of bias due to deviations from the intended interventions	Domain 3--Missing outcome data	Domain 4--Risk of bias in measurement of the outcome	Domain 5--Risk of bias in selection of the reported result	Overall Risk of Bias
Andrews, 2020[1]	Some concerns	Low	Low	Low	Low	Some concerns
Balaban, 2017[2]	Some concerns	Low	High	Low	Low	High
Berkowitz, 2019[3]	Low	Low	Some concerns	Low	Low	Some concerns
Birkhead, 1995[4]	Some concerns	Some concerns	Some concerns	Some concerns	Some concerns	Some concerns
Bovell-Ammon, 2020[5]	Some concerns	Low	Some concerns	Some concerns	Low	Some concerns
Bronstein, 2015[6]	Low	Some concerns	Low	Low	Some concerns	Low
Brown, 2022[7]	Some concerns	Low	Low	Low	Low	Some concerns
Bryce, 2021[8]	High	Low	High	Low	Low	High
Burnam, 1995[9]	Some concerns	Low	High	Some concerns	Some concerns	High
Carter, 2021[10]	Low	Low	Low	Low	Low	Low
Cauce, 1994[11]	High	Some concerns	Some concerns	Some concerns	Low	High
Cheng, 2008[12]	Low	Low	Some concerns	Low	Low	Some concerns
Corrigan, 2017[13]	Some concerns	Low	Some concerns	Some concerns	Low	Some concerns
Counsell, 2007[14]	Low	Low	Low	Low	Low	Low
Cox, 1998[15]	Low	Low	High	Some concerns	Low	High
Dixon, 2009[16]	Some concerns	Low	Some concerns	Low	Low	Some concerns
Duncan, 2020[17]	Low	Low	Low	Low	Low	Low
Eismann, 2022[18]	Some concerns	Some concerns	Low	Low	Low	Some concerns
Ell, 2017[19]	Low	Low	Some concerns	Low	Low	Some concerns
Ferrer, 2019[20]	Low	Low	High	Low	Low	High
Finkelstein, 2020[21]	Low	Low	Low	Low	Low	Low

© 2024 Viswanathan M et al. *JAMA Network Open.*

Author Year	Domain 1--Risk of bias arising from the randomization process	Domain 2--Risk of bias due to deviations from the intended interventions	Domain 3--Missing outcome data	Domain 4--Risk of bias in measurement of the outcome	Domain 5--Risk of bias in selection of the reported result	Overall Risk of Bias
Gottleib, 2020[22]	Low	Low	Some concerns	Low	Low	Some concerns
Gottlieb, 2016[23]	Low	Low	Some concerns	Low	Low	Some concerns
Guevara, 2020[24]	Low	Low	Some concerns	Low	Low	Some concerns
Hannan, 2016[25]	Some concerns	Low	Some concerns	Some concerns	Low	Some concerns
Heisler, 2022[26]	Some concerns	Low	Some concerns	Low	Low	Some concerns
Henschen, 2021[27]	Low	Low	Low	Low	Low	Low
Herman, 2000[28]	Some concerns	Some concerns	High	Some concerns	Some concerns	High
Hilgeman, 2014[29]	Low	Low	Low	Low	Low	Low
Horwitz, 2005[30]	Low	Low	Low	Low	Low	Low
Johnson, 2022[31]	Some concerns	Low	High	Some concerns	Low	High
Kangovi, 2014[32]	Low	Low	Low	Low	Low	Low
Kangovi, 2017[33]	Low	Low	Low	Low	Low	Low
Kangovi, 2018[34]	Low	Low	Some concerns	Low	Low	Some concerns
Kelley, 2020[35]	Low	Low	Low	Low	Low	Low
Kempainen, 2023[36]	Some concerns	Some concerns	Low	Low	Low	Some concerns
Kim, 2013[37]	Some concerns	Low	Low	Some concerns	Low	Some concerns
Kneipp, 2011[38]	Low	Low	Low	Some concerns	Low	Some concerns
Korr, 1996[39]	Some concerns	Low	High	High	Low	High
Krieger, 1999[40]	Low	Low	High	Low	Low	High
Krieger, 2009[41]	Low	Low	Some concerns	Some concerns	Low	Some concerns
Krieger, 2015[42]	Low	Low	Low	Some concerns	Low	Some concerns
Lin, 2017[43]	Some concerns	Some concerns	Low	Low	Low	Some concerns
Lipton, 1988[44]	Some concerns	Low	Low	Some concerns	Low	Some concerns

© 2024 Viswanathan M et al. *JAMA Network Open.*

Author Year	Domain 1--Risk of bias arising from the randomization process	Domain 2--Risk of bias due to deviations from the intended interventions	Domain 3--Missing outcome data	Domain 4--Risk of bias in measurement of the outcome	Domain 5--Risk of bias in selection of the reported result	Overall Risk of Bias
Liss, 2019[45]	Some concerns	Some concerns	Low	Low	Low	Some concerns
Lopez, 2023[46]	Some concerns	Low	Some concerns	Low	Low	Some concerns
MacKinney, 2013[47]	High	Some concerns	High	Low	Some concerns	High
McClintock, 2017[48]	Some concerns	Low	Low	Low	Low	Some concerns
Melnikow, 1997[49]	Low	Low	Low	Low	Low	Low
Mion, 2003[50]	Low	Low	Some concerns	Low	Low	Some concerns
Nyamathi, 2001[51]	Some concerns	Low	High	Some concerns	Low	High
O'Brien, 1999[52]	Low	Low	Low	Low	Low	Low
O'Connell, 2018[53]	Some concerns	Low	Low	Low	Low	Some concerns
O'Toole, 2015[54]	Some concerns	Low	Low	Low	Low	Some concerns
Post, 2021[55]	High	Low	Low	Low	Low	High
Raven, 2020[56]	Some concerns	Some concerns	Some concerns	Low	Some concerns	Some concerns
Sadowski, 2009[57]	Low	Low	Some concerns	Low	Low	Some concerns
Schickedanz, 2023[58]	Some concerns	Low	Low	Low	Low	Some concerns
Schumacher, 2017[59]	Low	Low	High	High	Low	High
Sege, 2015[60]	Low	Low	High	Low	Low	High
Shinn, 2015[61]	Some concerns	Low	Some concerns	Low	Low	Some concerns
Shumway, 2008[62]	Low	Low	Low	Low	Low	Low
Sood, 2021[63]	Some concerns	Low	Some concerns	Some concerns	Low	Some concerns
Talavera, 2021[64]	Some concerns	Low	Some concerns	Low	Some concerns	Some concerns
Theeke, 2016[65]	Some concerns	Low	Some concerns	Some concerns	Low	Some concerns
Tomita, 2012[66]	Low	Low	High	Low	Low	High
Toro, 1997[67]	Some concerns	Low	High	Some concerns	Low	High

© 2024 Viswanathan M et al. *JAMA Network Open.*

Author Year	Domain 1--Risk of bias arising from the randomization process	Domain 2--Risk of bias due to deviations from the intended interventions	Domain 3--Missing outcome data	Domain 4--Risk of bias in measurement of the outcome	Domain 5--Risk of bias in selection of the reported result	Overall Risk of Bias
Towfighi, 2021[68]	Low	Low	Low	Low	Low	Low
Waitzkin, 2011[69]	High	High	Some concerns	Some concerns	Some concerns	High
Williams, 2006[70]	Low	Low	High	Some concerns	Low	High
Wu, 2019[71]	Low	Low	Some concerns	Some concerns	Low	Some concerns
Zulman, 2017[72]	Some concerns	Some concerns	Low	Low	Low	Some concerns

© 2024 Viswanathan M et al. *JAMA Network Open.*

eTable 15. iCAT SR Dimensions, Assessment Categories, and Elaboration and Explanations

iCAT Dimension No.	iCAT Dimension Dimension-Specific Explanation[73]	Assessment Category	Assessment Category-specific Explanation[73]	Assessment Category-Specific Explanation/Elaboration[73,74] (Cochrane Methods, 2016)
1	Active components included in the intervention, in relation to the comparison.<br>An intervention component is defined as a discrete, active element of the intervention that could be implemented independently of other elements. Components vary in number and could be delivered independently of each other or be grouped together in organized bundles or looser packages of care.	More than one component and delivered as a bundle	The intervention includes more than one component and some or all of these components need to be delivered as a bundle.	A bundle is a set of intervention components that are intended to be used together to improve patient outcomes. It is expected that all of the elements of the bundle must be performed in a series of steps by one healthcare team (or other entity) within a particular timeframe for the intervention to impact on the designated outcomes. Changing a step in the process may alter the intended effect. For example, the ventilator bundle, developed to prevent ventilator-associated complications, comprises four core components: administering deep vein prophylaxis; administering medications to prevent gastric ulceration; elevating the head of the bed between 30 and 45 degrees; and providing a daily break in sedation for the purpose of assessing whether the patient can breathe independently of the ventilator.
		More than one component	The intervention includes more than one component. These components may be integrated into a package.	An integrated package is a group of intervention components that are intended to be used together but do not necessarily need to be performed in a specific order or timeframe, or simultaneously, to impact on the designated outcomes. For instance, intervention components may target different members of multi-professional teams or different levels of care such as primary, secondary and tertiary care. An integrated package could bring together different facets of care such as inputs, delivery, management and organization of services related to diagnosis, treatment, care, rehabilitation and health promotion; it could involve the integration of health services with social care, housing, education etc. For example, the NICE quality standard on drug use disorders [7] states that people accessing drug treatment should be offered a comprehensive assessment of their drug use and resources for recovery; an assessment of their personal, social and mental health needs in relation to family and carers; and support to access services which promote recovery and reintegration including housing, education, employment, personal finance, healthcare and mutual aid.

© 2024 Viswanathan M et al. *JAMA Network Open.*

iCAT Dimension No.	iCAT Dimension Dimension-Specific Explanation[73]	Assessment Category	Assessment Category-specific Explanation[73]	Assessment Category-Specific Explanation/Elaboration[73,74] (Cochrane Methods, 2016)
		One component	The intervention includes one component only.	*No category-specific explanation or elaboration*
		Varies	Varies across interventions to be considered for/included in the review.	Note that if this category is selected, review authors should consider whether the interventions included in the review are as similar as originally thought and whether this has implications for the review's inclusion criteria.
2	Behaviour or actions of intervention recipients or participants to which the intervention is directed. Behaviours or actions include taking a medication, changing a particular practice, improving knowledge or undergoing a surgical procedure; they may also include not undertaking a behaviour, such as not smoking. Behaviours or actions are targeted by the active components of the intervention.	Multi-target	Intervention directed at three or more behaviours or actions.	*No category-specific explanation or elaboration*
		Dual target	Intervention directed at two behaviours or actions.	If the intervention is directed at a set of linked behaviours, e.g., the implementation of a guideline for a range of actions (such as asthma medication, education and monitoring); self- monitoring; or fall prevention in the elderly, then it should be graded as multi-target (linked) or dual target (linked).
		Single target	Intervention directed at one behaviour or action only	A single dose single drug intervention is targeted at one action – taking a medication – and would therefore be scored as single target. In contrast, chronic medication involves prolonged repetition of a single action and should therefore be scored as multi target (linked). Other examples of a single behaviour or action include interventions to promote handwashing; to install fire alarms; and to use safety belts in cars.
		Varies	Varies across interventions to be considered for/ included in the review.	Note that if this category is selected, review authors should consider whether the interventions included in the review are as similar as originally thought and whether this has implications for the review's inclusion criteria.
3	Organisational levels and categories targeted by the intervention. Level refers to whether the intervention was directed at individuals (consumers, professionals, policy makers); groups or teams of individuals (staff of clinics, patient support groups, surgical teams etc.); or systems (communities, health systems, organisations (such as	Multi-level	Intervention directed at two or more levels.	*No category-specific explanation or elaboration*
		Multi-category	Intervention directed at two or more categories of individuals within the individual level (e.g., primary care professionals and primary care patients).	*No category-specific explanation or elaboration*
		Single category	Intervention directed only at single category of individuals within the individual level (e.g., professionals or patients or policy makers).	For cluster randomized trials, consider the levels targeted by the intervention. If the intervention is directed towards groups of clinicians and individual patients, it should be assessed as 'multi- category'. If the intervention is directed towards groups of clinicians

© 2024 Viswanathan M et al. *JAMA Network Open.*

iCAT Dimension No.	iCAT Dimension Dimension-Specific Explanation[73]	Assessment Category	Assessment Category-specific Explanation[73]	Assessment Category-Specific Explanation/Elaboration[73,74] (Cochrane Methods, 2016)
	hospitals), policy networks). Categories are groups, such as nurses or patients, within those levels			or groups of patients, it should be assessed as 'single category' as this constitutes only one category of individuals (see footnote above). If the intervention is directed at some of the individuals within a cluster rather than at the cluster as a whole, e.g., an intervention directed at a physician within a multi-professional primary care centre rather than at all the healthcare professionals in the centre, then the intervention should be assessed as 'single category'.
4	The degree of tailoring intended, or flexibility permitted across sites or individuals in applying or implementing the intervention. Tailoring implies that the intervention is intended to be modified for specific individuals, settings or circumstances, whereas flexibility implies leeway for modification if desired. Interventions may be modifiable in both content (e.g., variation in the components received by sites or individuals) and form (variation in the ways in which the components are delivered across sites or individuals).	Highly tailored/flexible	High degree of variation in implementation from site to site permitted and/or intervention designed to tailor to individuals or specific implementation settings.	*No category-specific explanation or elaboration*
		Moderately tailored/flexible	Some variation in implementation from site to site permitted (i.e., some components of the intervention are tailored/flexible while others are not).	For example, an intervention may include tailored counselling for patients, standardised patient materials and a standardised medical assessment. Most forms of counselling and related interventions, such as psychotherapy, are tailored towards individual needs and would therefore be expected to vary across individuals.
		Inflexible	Intervention implementation highly standardised with minimal variation from site to site.	For example, a standardised, non-tailored reminder letter; the insertion of a cardiac pacemaker following a standard protocol; or a standardised drug regimen.
		Varies	Varies across interventions to be considered for/included in the review.	Note that if this category is selected, review authors should consider whether the interventions included in the review are as similar as originally thought and whether this has implications for the review's inclusion criteria.
5	The level of skill required by those delivering the intervention in order to meet the intervention objectives. Skill is defined as the ability to do something, such as deliver a health promotion message appropriately or provide supportive supervision to health workers, arising from training,	High level skills	Extensive specialised skills required, i.e., new skills in addition to expected existing skills AND/OR the extension of existing skills to a highly specialised area AND/OR skills requiring extensive additional training.	Skill is defined as the ability to do something, arising from training, practice or experience.
		Intermediate level skills	Some specialised skills required, i.e., a small	*No category-specific explanation or elaboration*

© 2024 Viswanathan M et al. *JAMA Network Open.*

iCAT Dimension No.	iCAT Dimension Dimension-Specific Explanation[73]	Assessment Category	Assessment Category-specific Explanation[73]	Assessment Category-Specific Explanation/Elaboration[73,74] (Cochrane Methods, 2016)
	practice or experience. Different levels of skills may be required to deliver different interventions.		extension to the expected existing skills of professionals, decision makers or consumers.	
		Basic skills	No specialised skills required.	*No category-specific explanation or elaboration*
		Varies	Varies across interventions to be considered for/included in the review.	Note that if this category is selected, review authors should consider whether the interventions included in the review are as similar as originally thought and whether this has implications for the review's inclusion criteria.
6	The level of skill required for the targeted behaviour when entering the included studies by those receiving the intervention, in order to meet the intervention objectives.<br>Those receiving an intervention, such as an educational programme, may need skills, based on training, experience or practice, to interpret the information provided and then to apply it in their setting. For example, consumers may need a certain level of internet and health literacy to access and use health information. Again, different levels of skills may be required for different interventions	High level skills	Extensive specialised skills required.	For professionals, this is defined as basic professional training AND additional training of some sort (e.g., as a specialist physician or in the use of a particular procedure or technique).
		Intermediate level skills	Some specialised skills required.	For professionals, this is defined as their basic professional training, e.g., as a physiotherapist or nurse. If the recipient group includes a mix of 'non-specialist' and specialist health care providers (e.g., professional nurses and intensive care nurses) then, by implication, fewer specialist skills are required, and the intervention should be scored as 'intermediate'.<br>For patients / consumers, this category should be selected if the inclusion criteria for the review specify that patients / consumers in included studies need specific skills to be eligible for entry (e.g. must be proficient in the use of internet search engines) or if patients / consumers are given specific training prior to the study entry to assist them in performing the targeted behaviour (e.g. training in the use of a particular medical device such as a home blood glucose meter).
		Basic skills	No specialised skills required.	For patients / consumers, this grade should always be chosen if all comers are enrolled into the included studies. This category should not be used for professionals unless they are not using their professional skills within the context of the intervention. For example, the provision of leaflets to specialist surgeons to encourage them to wash their hands more often on hospital wards should be assessed as 'basic' as handwashing does not require

© 2024 Viswanathan M et al. *JAMA Network Open.*

iCAT Dimension No.	iCAT Dimension Dimension-Specific Explanation[73]	Assessment Category	Assessment Category-specific Explanation[73]	Assessment Category-Specific Explanation/Elaboration[73,74] (Cochrane Methods, 2016)
				any specialised or professional skills on the part of surgeons.
		Varies	Varies across interventions to be considered for/included in the review.	Note that if this category is selected, review authors should consider whether the interventions included in the review are as similar as originally thought and whether this has implications for the review's inclusion criteria.
7	The degree of interaction between intervention components, including the independence/interdependence of intervention components<br>The effectiveness of an intervention may depend on the combination of components delivered and/or the sequence of delivery. There may be synergistic ("added value") or dysynergistic effects from delivering intervention components in a particular combination, and one component delivered alone could be effective, ineffective or even harmful.	High level interaction	There is substantial interaction or inter-dependency between intervention components or actions i.e., the delivery of one intervention component impacts on the delivery of another, resulting in a synergistic effect.	*No category-specific explanation or elaboration*
		Moderate interaction	There is some degree of interaction but no evidence of synergistic effects or dysynergistic effects.	*No category-specific explanation or elaboration*
		Independent	The intervention has only one component or action, or the components act independently.	*No category-specific explanation or elaboration*
		Varies	Varies across interventions to be considered for/included in the review.	Note that if this category is selected, review authors should consider whether the interventions included in the review are as similar as originally thought and whether this has implications for the review's inclusion criteria.
		Unclear/unable to assess		*No category-specific explanation or elaboration*
8	The degree to which the effects of the intervention are dependent on the context or setting in which it is implemented.<br>The effects of an intervention may be dependent on the societal, political, economic, health system or environmental context in which the intervention	Highly context dependent	The effects of the intervention are likely to be strongly dependent on the implementation setting.	*No category-specific explanation or elaboration*
		Moderately context dependent	The effects of the intervention are likely to be transferrable across a limited range of settings only (e.g., only within a specific country or health system).	*No category-specific explanation or elaboration*

© 2024 Viswanathan M et al. *JAMA Network Open.*

iCAT Dimension No.	iCAT Dimension Dimension-Specific Explanation[73]	Assessment Category	Assessment Category-specific Explanation[73]	Assessment Category-Specific Explanation/Elaboration[73,74] (Cochrane Methods, 2016)
	is delivered. For example, an intervention may not have the same effects in primary care clinics and tertiary level hospitals, or in a health system in which care is free at the point of contact compared to one in which that is not the case.	Independent of context	The effects of the intervention do not appear to be strongly dependent on the implementation setting, i.e., it is anticipated that the effects of the intervention will be similar across a wide range of contexts or settings.	*No category-specific explanation or elaboration*
		Varies	Varies across interventions to be considered for/included in the review.	Note that if this category is selected, review authors should consider whether the interventions included in the review are as similar as originally thought and whether this has implications for the review's inclusion criteria.
		Unclear/unable to assess		*No category-specific explanation or elaboration*
9	The degree to which the effects of the intervention are changed by recipient or provider factors. The effects of an intervention may be dependent on the recipient's readiness for behaviour change or the proficiency of the person delivering the intervention.	Highly dependent on individual-level factors	The effects of the intervention are modified by both recipient and provider factors.	*No category-specific explanation or elaboration*
		Moderately dependent on individual-level factors	The effects of the intervention are modified by one of recipient or provider factors.	*No category-specific explanation or elaboration*
		Largely independent of individual-level factors	The effects of the intervention are not modified substantially by recipient or provider factors.	*No category-specific explanation or elaboration*
		Varies	Varies across interventions to be considered for/included in the review.	Note that if this category is selected, review authors should consider whether the interventions included in the review are as similar as originally thought and whether this has implications for the review's inclusion criteria.
		Unclear/unable to assess		*No category-specific explanation or elaboration*
10	The nature of the causal pathway between the intervention and the outcome it is intended to affect. This refers to pathways that involve human actions (such as behaviours) or actions within	Pathway variable, long	The causal pathway includes three or more steps between intervention and outcome or occurs over a long time period; is not linear or is variable; and/or more than one causal pathway has been proposed.	*No category-specific explanation or elaboration*

© 2024 Viswanathan M et al. *JAMA Network Open.*

iCAT Dimension No.	iCAT Dimension Dimension-Specific Explanation[73]	Assessment Category	Assessment Category-specific Explanation[73]	Assessment Category-Specific Explanation/Elaboration[73,74] (Cochrane Methods, 2016)
	organisations or systems rather than biological pathways. The causal pathway for an intervention may be clear, direct, short and linear or it may be longer, more variable, or there may be more than one causal pathway	Pathway linear, long	The causal pathway is linear but there are three or more steps between intervention and outcome.	*No category-specific explanation or elaboration*
		Pathway linear, short	The causal pathway is clear, short (only one or two steps), direct, linear.	*No category-specific explanation or elaboration*
		Varies	Varies across interventions to be considered for/included in the review.	Note that if this category is selected, review authors should consider whether the interventions included in the review are as similar as originally thought and whether this has implications for the review's inclusion criteria.
		Unclear/unable to assess		*No category-specific explanation or elaboration*

© 2024 Viswanathan M et al. *JAMA Network Open.*

eTable 16. RCT Abstraction Form Items Adapted from iCAT[73,74]

Abstraction Item Wording[a]<br>Tooltip[b]<br>Link to iCAT dimension explanation/elaboration	iCAT Dimension No.	Assessment Categories[c]
How was the intervention or intervention components intended to be delivered?<br>***Tooltip***: *"Components can be described as essential functions or principles, and associated elements and intervention activities that are judged necessary to produce desired outcomes." (HHS ASPE) Do not count usual care, standard of care or treatment as usual as a component.*<br>***Link***: *Not provided*	1	More than one component and some or all delivered as a bundle
		More than one component; may be integrated into a package
		One component
How many behaviors or actions did the intervention intend to address?<br>***Tooltip***: *Behaviors or actions include taking a medication, changing a particular practice, improving knowledge or undergoing a surgical procedure; they may also include not undertaking a behavior, such as not smoking. Behaviors or actions are targeted by the active components of the intervention.*<br>***Link:*** *See iCAT Dimension 2 for more information*	2	Three or more
		Two
		One
Indicate the degree of tailoring intended or flexibility permitted across sites or individuals in applying or implementing the intervention[d]<br>***Tooltip***: *Tailoring or adaptation may be described in terms of consideration of factors such as language, persons delivering intervention, metaphors, content, goals, methods, context (Ecological Validity framework); specific modifications such as adding, deleting, shortening, lengthening, substituting, reordering, repeating elements, integrating other approaches, loosening structure, changing setting, population, delivery mode/medium, deliverer, training, evaluation procedures, core elements.[75]*<br>***Link:*** *See iCAT Dimension 4 for more information*	4	Highly tailored/very flexible
		Moderately tailored/moderately flexible
		Minimally tailored/slightly flexible
What level of skill was required by those delivering the intervention in order to meet the intervention objectives?<br>***Tooltip:*** *Not provided*<br>***Link:*** *See iCAT Dimension 5 for more information*	5	High
		Intermediate
		Basic
What level of skill was required for the targeted behavior or action at baseline among those receiving the intervention in order to meet the intervention objectives?<br>***Tooltip:*** *Not provided*<br>***Link:*** *See iCAT Dimension 6 for more information*	6	High
		Intermediate
		Basic
What was the degree of interaction between intervention components, including the independence/interdependence of intervention components?<br>***Tooltip:*** *Not provided*<br>***Link:*** *See iCAT Dimension 7 for more information*	7	High degree of interaction or interdependence
		Moderate degree of interaction or interdependence
		No interaction/ independent
		Unclear or unable to assess
Indicate the degree to which the effects of the intervention are dependent on the context or setting in which it is implemented.<br>***Tooltip:*** *Not provided*<br>***Link***: *See iCAT Dimension 8 for more information*	8	Highly dependent on context or setting
		Moderately dependent on context or setting
		Independent of context or setting
		Unclear or unable to assess
	9	Highly dependent on individual-level factors

© 2024 Viswanathan M et al. *JAMA Network Open.*

Abstraction Item Wording[a]<br>Tooltip[b]<br>Link to iCAT dimension explanation/elaboration	iCAT Dimension No.	Assessment Categories[c]
Indicate the degree to which the effects of the intervention are changed by individual-level factors (ie, recipient or provider factors)<br>***Tooltip:*** *Not provided*<br>***Link:*** *See iCAT Dimension 9 for more information*		Moderately dependent on individual-level factors<br>Independent of individual-level factors<br>Unclear or unable to assess

[a] We did not use the iCAT items 3, 5, 6, 7, and 10 because of poor reporting and/or poor reliability of extraction.

[b] Tooltips and Links to iCAT dimension elaborations and explanations were provided as needed and determined during pilot testing of the abstraction form. All abstractors had access to the full documentation provided in Lewin et al (2017) and the Cochrane Methods (2016) document.

[c] We modified categories based on feedback from the team during pilot testing of the abstraction form; we did not include the category “varies” as it is specific to systematic reviews (planned or conducted) overall; for this project, abstraction was limited and specific to the intervention described in each of the eligible RCTs.

[d] This was a conditional item that appeared when the answer to the prior item “Was tailoring, adaptation, or flexibility of the intervention intended (eg, provision of culturally appropriate materials) or permitted (eg, accommodations to meet an unexpected need)?” was “Yes, the intervention was tailored, could be adapted, and/or delivered flexibly.”

© 2024 Viswanathan M et al. *JAMA Network Open.*

eFigure 2. Article Flow

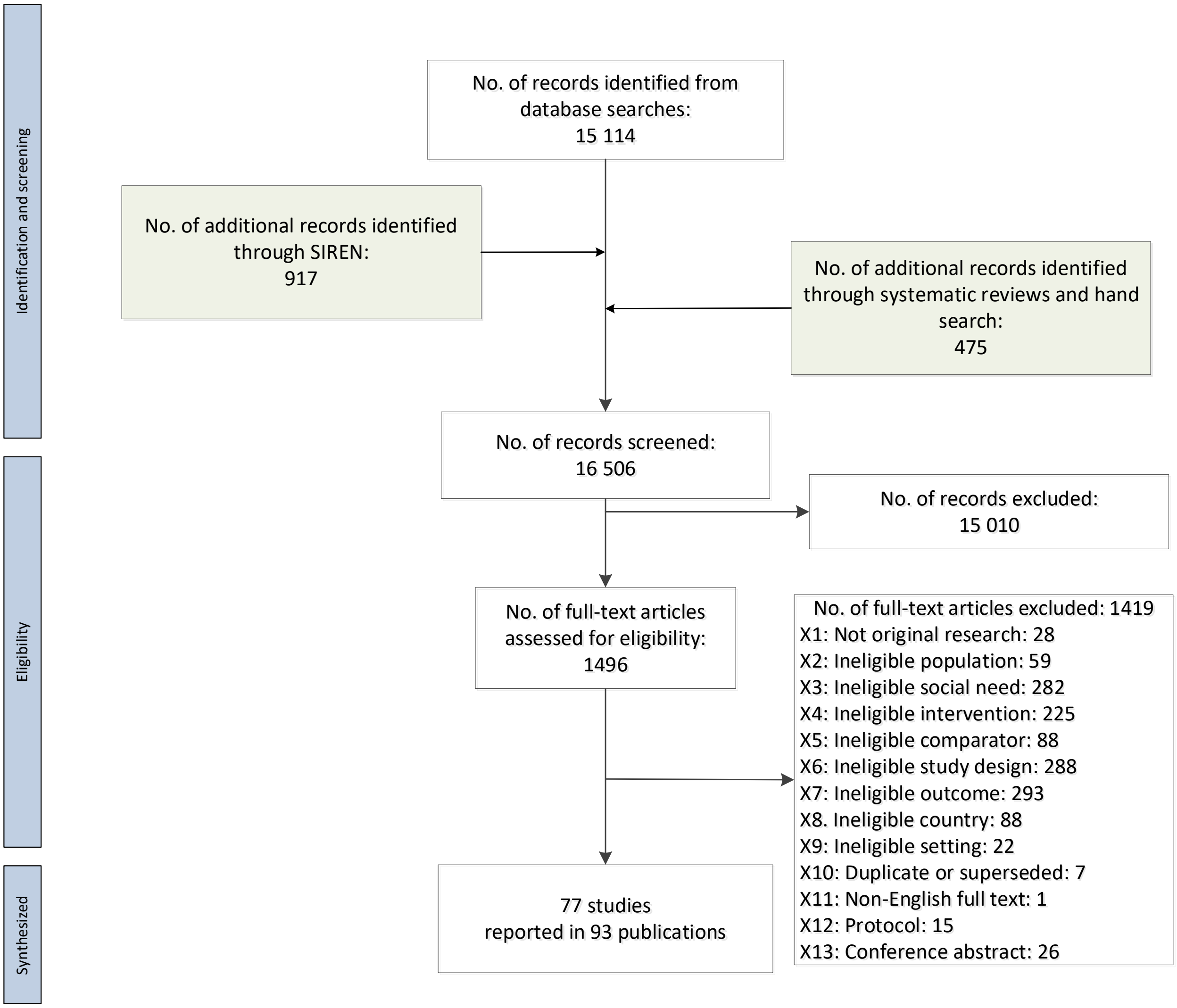

Abbreviations: No. = Number , X = Exclusion.

eTable 17. Description of how Social Needs Were Identified

How are the social needs of the target population identified? (N=77 unless noted otherwise)	N (%)
**Was disadvantage defined?**	
Yes	49 (64)
No	28 (36)
**Among those that defined disadvantage (n = 49), how was disadvantage defined?**	
Individual	32 (65)
Community	11 (22)
Both	6 (12)
**Inclusion criteria that mapped to a PROGRESS-Plus category, among those with an inclusion criteria that mapped to at least one category (n = 56)**	
Place of residence	12 (21)
Race/ethnicity/culture/language	4 (7)
Occupation	2 (4)
Gender/sex	4 (7)
Socioeconomic status	39 (70)
Personal characteristics associated with discrimination (eg, age, disability)	10 (18)
Features of relationships (eg, parents who smoke, excluded from school)	2 (4)
Time-dependent relationships (eg, leaving the hospital, respite care, other instances where a person may be temporarily at a disadvantage)	18 (32)
None of the above	21 (--)
**How was the population identified with reference to the social need addressed by the intervention?**	
Individuals were screened using study eligibility criteria (social need was an inclusion criterion)	34 (44)
Study participants were assessed for social need (social need was not an inclusion criterion)	9 (12)
High prevalence of the social need in the population	7 (9)
High prevalence of a proxy for the social need in the population	21 (27)
Study does not provide any justification or explanation	6 (8)
**How was the intervention developed to respond to community or individual social need(s)?**	
Community advisory board or other community input	17 (22)
Prevalence of social need(s) in the community	22 (29)
No justification or explanation	34 (44)
Other	12 (16)
**Does the study explicitly address health equity?**	
Explicit goals	6 (8)
In title or background or discussion, but not in explicit goals	23 (30)
Not explicit	48 (62)

© 2024 Viswanathan M et al. *JAMA Network Open.*

eTable 18. Measures of Intervention Intensity and Complexity

	Intensity					Complexity								
Author, Year	≥ 8 Contacts or Varied by Need[a]	Contacts at Least Every 2 Weeks or Varied by Need[a]	≥ 30 Minutes or Varied by Need[a]	Contact Duration ≥ 6 Months or Varied by Need[a]	Home Visit	Number of Specified Social Needs Addressed	Staff-Mediated Patient/ Health System Interaction[b]	Multiple Service Providers Involved	Multiple Interven-tion Compo-nents (iCAT dimension 1)	Active Assistance with Resources	Multiple Participant Behaviors Targeted[c] (iCAT dimension 2)	Multiple Resources Offered to Participants	Multiple Resources Required to Implement Intervention	Tailoring Permitted (iCAT dimension 4)
**Medical and social needs program**														
Birkhead, 1995[4]						2				✓				
Bovell-Ammon, 2020[5]	✓			✓		4		✓	✓	✓	✓	✓	✓	✓
Bronstein, 2015[6]	✓	✓			✓	4	✓		✓	✓	✓		✓	✓
Brown, 2022[7]	✓			✓	✓	6	✓	✓	✓	✓	✓	✓	✓	✓
Burnam, 1995[9]	✓					4	✓	✓	✓	✓			✓	✓
Caskey, 2019[81]				✓	✓	2	✓	✓	✓	✓	✓		✓	✓
Cauce, 1994[11]	✓					9	✓		✓	✓	✓	✓	✓	✓
Counsell, 2007[14]	✓			✓	✓	1	✓	✓	✓	✓	✓	✓	✓	✓
Duncan, 2020[17]						4	✓	✓	✓	✓	✓	✓	✓	✓
Ell, 2017[19]	✓				✓	1	✓		✓	✓	✓	✓	✓	✓
Finkelstein, 2020[21]	✓				✓	3	✓	✓	✓	✓	✓	✓	✓	✓
Hannan, 2016[25]	✓	✓				2			✓	✓	✓		✓	✓
Henschen, 2021[27]	✓					4	✓	✓	✓	✓	✓	✓	✓	✓
Kangovi, 2014[32]	✓				✓	5	✓		✓	✓	✓	✓	✓	✓
Kangovi, 2017[33]	✓	✓		✓		5	✓		✓	✓	✓		✓	✓
Kangovi, 2018[34]	✓	✓		✓		4	✓		✓	✓	✓		✓	✓
Kneipp, 2011[38]	✓		✓	✓	✓	1	✓		✓	✓	✓	✓	✓	✓
Krieger, 2009[41]	✓				✓	2	✓		✓	✓	✓	✓	✓	✓
Krieger, 2015[42]	✓	✓			✓	5	✓	✓	✓	✓	✓	✓	✓	✓
Lipton, 1988[44]	✓			✓		4	✓	✓	✓	✓			✓	✓
Liss, 2019[45]	✓					2	✓	✓	✓	✓	✓	✓	✓	✓

© 2024 Viswanathan M et al. *JAMA Network Open.*

Author, Year	Intensity					Complexity								
	≥ 8 Contacts or Varied by Need[a]	Contacts at Least Every 2 Weeks or Varied by Need[a]	≥ 30 Minutes or Varied by Need[a]	Contact Duration ≥ 6 Months or Varied by Need[a]	Home Visit	Number of Specified Social Needs Addressed	Staff-Mediated Patient/ Health System Interaction[b]	Multiple Service Providers Involved	Multiple Interven-tion Compo-nents (iCAT dimension 1)	Active Assistance with Resources	Multiple Participant Behaviors Targeted[c] (iCAT dimension 2)	Multiple Resources Offered to Participants	Multiple Resources Required to Implement Intervention	Tailoring Permitted (iCAT dimension 4)
MacKinney, 2013[47]						1		✓	✓	✓		✓	✓	
McClintock, 2017[48]			✓			5			✓		✓		✓	✓
Melnikow, 1997[49]						2				✓				
Nyamathi, 2001[51]		✓	✓			1	✓	✓	✓		✓	✓	✓	✓
Raven, 2020[56]	✓					4	✓	✓	✓	✓	✓	✓	✓	
Sadowski, 2009[57]	✓	✓		✓		1	✓		✓	✓	✓	✓	✓	✓
Schickedanz, 2023[58]	✓	✓	✓	✓		5	✓		✓	✓	✓	✓	✓	✓
Shumway, 2008[62]	✓			✓		4	✓	✓	✓	✓	✓		✓	✓
Sood, 2021[63]	✓					2	✓		✓	✓		✓	✓	✓
Talavera, 2021[64]	✓		✓			3	✓	✓	✓	✓		✓	✓	✓
Towfighi, 2021[68]	✓	✓			✓	3	✓	✓	✓	✓	✓	✓	✓	✓
Williams, 2006[70]				✓	✓	2	✓		✓	✓	✓	✓	✓	✓
Zulman, 2017[72]	✓	✓		✓	✓	3	✓	✓	✓	✓	✓	✓	✓	✓
**Social needs only intervention**														
Andrews, 2020[1]						2	✓	✓	✓	✓	✓	✓		✓
Balaban, 2017[2]	✓					2	✓		✓	✓	✓		✓	✓
Berkowitz, 2019[3]				✓		1				✓	✓	✓		✓
Berkowitz, 2018[78]	✓	✓				1				✓			✓	✓
Bryce, 2021[8]	✓	✓				1			✓			✓	✓	
Carter, 2021[10]	✓				✓	4	✓		✓	✓	✓	✓	✓	✓
Cheng, 2008[12]	✓				✓	2	✓	✓	✓	✓	✓	✓	✓	✓
Corrigan, 2017[13]	✓	✓				2	✓		✓		✓	✓	✓	✓
Cox, 1998[15]	✓			✓		3	✓		✓	✓			✓	✓

© 2024 Viswanathan M et al. *JAMA Network Open.*

	Intensity					Complexity								
Author, Year	≥ 8 Contacts or Varied by Need[a]	Contacts at Least Every 2 Weeks or Varied by Need[a]	≥ 30 Minutes or Varied by Need[a]	Contact Duration ≥ 6 Months or Varied by Need[a]	Home Visit	Number of Specified Social Needs Addressed	Staff-Mediated Patient/ Health System Interaction[b]	Multiple Service Providers Involved	Multiple Interven-tion Compo-nents (iCAT dimension 1)	Active Assistance with Resources	Multiple Participant Behaviors Targeted[c] (iCAT dimension 2)	Multiple Resources Offered to Participants	Multiple Resources Required to Implement Intervention	Tailoring Permitted (iCAT dimension 4)
Dixon, 2009[16]	✓				✓	1	✓	✓	✓	✓	✓		✓	✓
Eismann, 2022[18]	✓		✓			1	✓		✓		✓	✓	✓	✓
Ferrer, 2019[20]	✓			✓	✓	1	✓	✓	✓			✓		✓
Flores, 2018[80]						1	✓			✓			✓	
Gottleib, 2020[22]		✓				2	✓		✓	✓	✓	✓	✓	✓
Gottlieb, 2016[23]	✓	✓				9	✓		✓	✓		✓	✓	✓
Guevara, 2020[24]						1		✓	✓			✓	✓	
Heisler, 2022[26]	✓			✓		1	✓		✓		✓		✓	✓
Herman, 2000[28]	✓			✓	✓	3	✓		✓	✓	✓		✓	✓
High, 2000[79]				✓		1					✓	✓	✓	
Hilgeman, 2014[29]	✓		✓			1	✓		✓	✓			✓	✓
Horwitz, 2005[30]						1	✓			✓			✓	
Johnson, 2022[31]	✓					5	✓				✓		✓	✓
Kelley, 2020[35]	✓	✓		✓		4	✓	✓	✓	✓	✓	✓	✓	✓
Kempainen, 2023[36]	✓	✓		✓		1		✓	✓	✓		✓	✓	✓
Kim, 2013[37]	✓			✓		3	✓		✓			✓	✓	✓
Korr, 1996[39]	✓					4			✓	✓	✓		✓	✓
Krieger, 1999[40]					✓	1	✓		✓	✓			✓	✓
Lin, 2017[43]	✓			✓	✓	5	✓		✓	✓	✓	✓	✓	✓
Lopez, 2023[46]		✓				9		✓			✓	✓	✓	
Lumba-Brown,2020[82]				✓	✓	1	✓		✓	✓	✓		✓	✓
Mion, 2003[50]	✓					0			✓		✓		✓	✓
O'Brien, 1999[52]						1			✓	✓		✓		

© 2024 Viswanathan M et al. *JAMA Network Open.*

Author, Year	Intensity					Complexity								
	≥ 8 Contacts or Varied by Need[a]	Contacts at Least Every 2 Weeks or Varied by Need[a]	≥ 30 Minutes or Varied by Need[a]	Contact Duration ≥ 6 Months or Varied by Need[a]	Home Visit	Number of Specified Social Needs Addressed	Staff-Mediated Patient/ Health System Interaction[b]	Multiple Service Providers Involved	Multiple Interven-tion Compo-nents (iCAT dimension 1)	Active Assistance with Resources	Multiple Participant Behaviors Targeted[c] (iCAT dimension 2)	Multiple Resources Offered to Participants	Multiple Resources Required to Implement Intervention	Tailoring Permitted (iCAT dimension 4)
O'Connell, 2018[53]	✓	Not reported	Not reported	Not reported		1	✓		✓	✓	✓			
O'Toole, 2015[54]			✓	Not reported		1		✓	✓	✓		✓	✓	✓
Post, 2021[55]	✓	Not reported	Not reported	Not reported		3	✓			✓	✓		✓	✓
Schumacher, 2017[59]		Not reported	Not reported	Not reported	✓	1			✓	✓	✓	✓	✓	✓
Sege, 2015[60]	✓	Not reported	Not reported	✓	✓	5	✓	✓	✓	✓	✓		✓	✓
Shinn, 2015[61]	✓	✓	Not reported	✓	✓	1	✓	✓	✓	✓		✓	✓	✓
Theeke, 2016[65]		✓	✓			1			✓		✓			
Tomita, 2012[66]	✓	Not reported	Not reported	✓	✓	2	✓		✓	✓	✓		✓	✓
Toro, 1997[67]	✓	Not reported				8	✓	✓	✓	✓	✓	✓	✓	✓
Waitzkin, 2011[69]	✓		Not reported	✓		4	✓	✓	✓	✓	✓		✓	✓
Wu, 2019[71]	Not reported		Not reported	Not reported		1		✓	✓		✓	✓		Not reported

[a] Studies offering features of intensity at or above the modal value (eg, 8 contacts, 30 minutes, 6 months) were grouped together in a category suggestive of higher intensity. Studies that varied intensity by patient need were also included in the high-intensity category because these interventions had to be designed to accommodate high intensity for at least some patients.
[b] Studies used varied terms and titles, including care coordination, case management, care management, community health workers, promotoras, peers, patient navigators, or other coordinators.
[c] Multiple behaviors for at least some participants but may have varied by participant and need (intervention Complexity Assessment Tool for Systematic Reviews [iCAT] dimension 2).
Key

✓	Yes
	No
(shaded)	Not reported

© 2024 Viswanathan M et al. *JAMA Network Open.*

eTable 19. Justification of Intervention Components

Author, Year	Justification for intervention from publication[a]	Intervention description	Multiple Intervention Comp-onents	Was the intervention developed to respond to community or individual social need(s)?	Justification for specific components
Andrews, 2020[1]	Experimental studies have demonstrated that temporary relief of financial strain can immediately improve elements of cognitive function that are critical for decision-making and task performance (Kaur et al 2019; Mani et al 2013).	1. Women received the same materials and support as those in the control arm (informational pamphlets on skin-to-skin care and breastfeeding, and support in obtaining a breast pump).<br><br>2. Women received a transfer of $200 to an ATM card each week her infant was in the NICU, up to a maximum of $600 to be used for whatever financial challenges or social needs they perceived as impediments to getting to the NICU and providing infant care, labeled with language to encourage visits to the NICU for skin-to-skin care.	Yes	Other; intervention designed based on the team's qualitative work, which described financial barriers and financial stress as significant obstacles to NICU visitation and providing infant care	Yes
Balaban, 2017[2]	Several care transitions programs11–16 have demonstrated success in decreasing hospital readmissions. These programs have primarily targeted elderly Medicare populations or patients with high-risk diagnoses, such as heart failure. However, key groups of challenging patients, some of whom are disproportionately served at safety-net hospitals, were excluded, or underrepresented in these studies,17,18 including nonelderly patients, non-English speakers, patients with dementia, those who leave against medical advice, and the homeless.<br>Furthermore, these programs used relatively expensive licensed personnel, such as nurse practitioners, pharmacists, or social workers. Safety-net hospitals typically have limited resources for new initiatives and professional staff. Less costly	Patient navigators met with intervention patients and families to describe program, assess post-discharge needs, schedule post-discharge call with navigator.<br>After discharge, navigators conducted weekly telephone calls to help arrange transportation to appointments, address barriers to medications, identify patient concerns and facilitate communication with primary care, reassess home care needs and make connections to services, assist	Yes	No justification or explanation	Justification not specific to individual components

© 2024 Viswanathan M et al. *JAMA Network Open.*

Author, Year	Justification for intervention from publication[a]	Intervention description	Multiple Intervention Comp-onents	Was the intervention developed to respond to community or individual social need(s)?	Justification for specific components
	community health workers (CHWs)19, 20 have increased medical follow-up with primary care and specialists,21–23 improved adherence to medical regimens,24, 25 and provided meaningful social support.22 These competencies may enhance post-discharge transitional care and reduce rehospitalizations. We implemented an intervention led by hospital-based CHWs, or patient navigators (PNs), designed to reduce readmissions among high-risk patients in a safety-net medical system. We assess the effects of this intervention in two distinct patient populations—those over and age 60—groups whose distinctive distribution of medical and psychiatric comorbidities26–28 and different networks of social support29,30 shape their post-discharge needs and potentially their response to the PN intervention.	with health insurance problems and supported patient self-management.			
Berkowitz, 2018[78]	Further, clinicians currently have few options for helping patients with food insecurity in routine care, and there is little evidence regarding whether clinical programs for individuals with food insecurity can improve diet.15<br>To address these research gaps, we sought to test the feasibility and short-term impact on diet of a program that delivered medically tailored meals to individuals with type 2 diabetes, food insecurity, and hyperglycemia (hemoglobin A1c >8.0%) in a randomized cross-over trial.<br><br>From Discussion<br>Prior, nonrandomized, pre-post studies of medically tailored meals23 and food pantry based interventions24 have also suggested benefit of the direct food provision, but the use of random assignment in this study strengthens the evidence base to support a causal effect between the medically tailored meal program and improved dietary quality.	Home delivery of medically tailored meals by Community Servings, a local nonprofit organization. Meals were prepared under the guidance of a registered dietitian to be suitable not only for the participant's diagnosis of diabetes but also for other medical conditions the participant might have across 17 possible tracks (eg, chronic kidney disease or anticoagulation using warfarin). Each individual could receive meals that followed up to 3 tracks (eg, diabetes and chronic kidney disease and anticoagulation using warfarin), allowing for meals closely tailored to the specific medical	No	Other; tailored to meet needs for diabetes and other diseases; delivered to ensure access	NA; single intervention

© 2024 Viswanathan M et al. *JAMA Network Open.*

Author, Year	Justification for intervention from publication[a]	Intervention description	Multiple Intervention Comp-onents	Was the intervention developed to respond to community or individual social need(s)?	Justification for specific components
		needs of the individual. While receiving the intervention, the participant received a once weekly delivery consisting of 10 refrigerated and/or frozen meals, designed to represent 5 lunches and 5 dinners (approximately half of the participant's weekly food intake). Meals were fully prepared and could be simply heated and consumed. Food was provided only for the study participant. To test the impact of meal delivery, no additional educational intervention was included during the on-meals period			
Berkowitz, 2019[3]	A promising approach is community-supported agriculture (CSA).16−19 A common small farm vegetable and fruit distribution model, CSAs ask that before the beginning of a growing season, individuals or households purchase a share of the produce of a local farm.19 This farm will then provide, generally weekly, an allotment of seasonal produce for the subscribers. Benefits of the CSA approach include the variety of seasonally available produce, connection with a local business, and a membership-based model, which may facilitate engagement.16,17,20 These features may lead to improved diet quality. A prior feasibility study in federally qualified health center patients found that CSA participation was associated with an increase in the variety of vegetables eaten.21	Participants were given $300 per growing season for study participation and were required to purchase a CSA share. Membership entitled the participant to a weekly share of the farm's produce from June to November. Each week, the farm also provided 2 recipes and basic information about the featured foods.	No	Prevalence of social need(s) in the community	NA; single intervention
Birkhead, 1995[4]	Approximately 5000 children were eligible for measles immunization, and more than 3000 of them were known to have been immunized as a result of the	Escort accompanied the child to the pediatric clinic (same	No	No justification or explanation	NA; single intervention

© 2024 Viswanathan M et al. *JAMA Network Open.*

Author, Year	Justification for intervention from publication[a]	Intervention description	Multiple Intervention Comp-onents	Was the intervention developed to respond to community or individual social need(s)?	Justification for specific components
	outbreak measures (NYS DOH, unpublished data, 1990). As a result of this success, in January 1991 the NYS DOH instituted a policy throughout the state to screen immunization status at the time of WIC certification and at least to refer eligible children for needed immunizations. The question remained, however, what were the most effective ways to encourage immunization through the WIC program?	facility) for express lane immunization.			
Bovell-Ammon, 2020[5]	While research has demonstrated the importance of interventions to address family homelessness, 16 there are few models specifically focused on housing-unstable or homeless families with children, adults, or both who have chronic conditions or disabilities or are using more health services than usual—henceforth referred to as "medically complex families."17 Additionally, we are aware of very few randomized studies that assessed longitudinal housing, child and adult health, and economic outcomes of supportive housing for this population. To test a supportive housing intervention with the objective of improving family health, the authors, who are researchers with Children's HealthWatch at Boston Medical Center (BMC), designed and implemented a pilot randomized controlled trial (called Housing Prescriptions as Health Care) through a collaborative process with human services agencies, a Medicaid managed care payer, legal experts, and a public housing authority. Medically complex families in particular face challenges associated with health and social needs. Previous research has demonstrated that these families have increased out-of-pocket spending associated with more use of medical and education services and are at risk of losing parental income, both of which are linked to greater economic hardship.18 Thus, an intervention that responds to families' lack of financial resources as well as time burdens through the coordination of services that	1. Multifaceted supports including community-based organizations to assist with search for housing, prevent eviction, provide legal and financial services<br><br>2. Assistance to obtain public housing (application assistance and wait-list priority)<br><br>3. Case management and wraparound care: coordinated delivery of services with healthcare providers to address need for accommodations and health-related services	Yes	No justification or explanation	Justification not specific to individual components

© 2024 Viswanathan M et al. *JAMA Network Open.*

Author, Year	Justification for intervention from publication[a]	Intervention description	Multiple Intervention Comp-onents	Was the intervention developed to respond to community or individual social need(s)?	Justification for specific components
	address housing, financial, legal, social, and health needs may improve housing and health outcomes.				
Bronstein, 2015[6]	Further investigation is needed to assess effective mechanisms for preventing readmissions that implement and support practices beyond health care utilization. In addition to collaborating with PCPs or follow-up healthcare services, various accompanying services have been identified as effective in preventing hospital readmissions (Grafft et al 2010; Greenwald et al 2007). Krumholz et al (2002) studied the effects of patient education and support interventions on readmission after discharge among heart failure patients. The intervention group patients received a face-to-face education within two weeks of their discharge and a series of follow-up phone calls (weekly for four weeks, followed with biweekly for eight weeks, and terminating with monthly calls for up to a year). These interventions were performed by a nurse who received training from an interprofessional team consisting of a pharmacist, geropsychiatric clinical nurse, cardiologist, and licensed social worker (Krumholz et al., 2002). The patients who received the intervention (n = 44) and those in the control group (n = 44) both received routine medical aftercare as recommended by their physicians. Results indicated a 39 percent reduction (p = .03) in readmission rates for the intervention group in comparison with the control. In addition, a cost–benefit analysis for the intervention services provided revealed a reduction in costs of $7,515 per patient. Corroborating the conclusion that dynamics relating to care coordination, which is a core skill of social workers, in combination with routine medical care can produce significant reductions in hospital readmission rates, additional studies shine light on the significance of contextual variables often addressed by social	1.Post-discharge care coordination: At discharge, participants assigned to the intervention group received follow-up care coordination provided by the Master of Social Work interns designed to assess, identify, and alleviate barriers to patients remaining at home. The students contacted the participants in the intervention group via a follow-up phone call within three to five days post-discharge.<br>2.After that, a home visit was scheduled and paid between days seven and 14. A final phone call was made at approximately day 21 post-discharge. Additional contacts were made with participants when necessary to provide appropriate care coordination.<br>3. At each point during the three primary points of intervention, interns conducted an individualized needs assessment, identifying medication concerns, transportation issues, home care needs, home safety concerns, and behavioral barriers to follow-up care and activities post-discharge. Interns were trained to empower	Yes	No justification or explanation	Justification not specific to individual components

© 2024 Viswanathan M et al. *JAMA Network Open.*

Author, Year	Justification for intervention from publication[a]	Intervention description	Multiple Intervention Comp-onents	Was the intervention developed to respond to community or individual social need(s)?	Justification for specific components
	workers, including sociodemographics, social systems, and managed care.	patients in identifying their own solutions, including seeking assistance from appropriate sources and proactively addressing concerns before they evolved into an emergency need. In addition, interns worked with participants to ensure timely follow-up with their primary care provider.			
Brown, 2022[7]	Healthcare–based programs assisting patients with social needs—such as housing, transportation, and food security—are gaining interest as a strategy to improve health and avoid unnecessary healthcare use, but uncertainty remains about their effects (1–4). Social needs assistance programs generally involve screening patients for social risks, offering referrals, helping with service navigation, and coaching patients to access social services and care (1). Programs vary widely on key dimensions that likely influence their effectiveness, such as how patients become eligible, qualifications of personnel in case manager or navigator roles, length of enrollment, range of health and social needs addressed, and whether programs offer direct assistance or only referrals. Few studies have used randomization to evaluate the effect of social needs case management on the health care use outcomes that interest payers and policymakers (1–3), and prior randomized studies have been too small for subgroup analyses to compare effectiveness across patient groups (5–8).	1. Participants are assigned to a case manager who contacts them to explain the program and offer services for 1 year through telephone, text messages, mail, home visits and communication with healthcare providers.<br><br>2. If participants accept services, case managers used a tool to administer social needs screening, including food, transportation, housing, finances, employment, legal support, safety, and social support, unmet medical, behavioral health, dental and vision care needs.<br><br>3. Worked collaboratively to develop a patient-centered care plan<br><br>4. Supported patients' progress on goals through coaching, help with applications for public benefits, communicating with	Yes	No justification or explanation	Justification not specific to individual components

© 2024 Viswanathan M et al. *JAMA Network Open.*

Author, Year	Justification for intervention from publication[a]	Intervention description	Multiple Intervention Comp-onents	Was the intervention developed to respond to community or individual social need(s)?	Justification for specific components
		health care providers and social service agencies, referrals to social services, direct access to resources (cell phones, emergency housing funds, legal aid).			
Bryce, 2021[8]	Limited research to date has examined clinical outcomes related to fruit and vegetable prescription programs; all have been single group, pre/post-program analyses (Forbes et al., 2019; Freedman et al 2013; Marcinkevage et al 2019; Omar et al., 2017; Trapl et al 2018; Weinstein et al 2014). These studies have demonstrated a significant decrease in body mass index (BMI) (Cavanagh et al 2017) and blood pressure (BP) (York et al 2020), as well as improvement in blood glucose control (Richie 2019; Snailer 2019). A 2015 study of patients with type 2 diabetes participating in a fruit and vegetable prescription program found participants experienced a significant decrease in hemoglobin A1C percentage (HbA1C) (ie, 9.54 to 8.83) (Bryce et al 2017). However, weight and BP did not change from pre- to post-study (p >.05) (Bryce et al., 2017). Although these results are encouraging, more rigorous investigation (ie, inclusion of a comparison group and randomization of participants) is needed to strengthen the degree of evidence of the impact of fruit and vegetable prescription programs.	1. Up to $80 ($10 per visit loaded onto a debit card for up to 8 visits) for purchase of fresh fruits and vegetables at that FQHCs farmers market (the Mercado). The Mercado, located outside the entrance to the health center, is a collection of several local produce farmers. The Mercado operated every Thursday (9 a.m. to 1 p.m.) and occurred over 15 weeks.<br><br>2. The Mercado also offers other positive health-promoting activities including cooking demonstrations, nutrition education and exercise events.	Yes	Community advisory board or other community input	Justification not specific to individual components
Burnam, 1995[9]	No studies in the literature to date have included an experimental design that randomizes subjects to conditions	1. Curriculum-based groups focused on mental health and substance abuse education and rehabilitation<br><br>2. Participation in 12-step programs<br><br>3. Process-oriented groups to	Yes	No justification or explanation	Justification not specific to individual components

© 2024 Viswanathan M et al. *JAMA Network Open.*

Author, Year	Justification for intervention from publication[a]	Intervention description	Multiple Intervention Comp-onents	Was the intervention developed to respond to community or individual social need(s)?	Justification for specific components
		facilitate discussion of issues of importance to clients<br><br>4. Individual counseling and case management<br><br>5. Psychiatric consultation and medication management<br><br>6. Community activities<br><br>7. Residential services			
Carter, 2021[10]	While most CHW studies have focused on specific disease-based cohorts,21-26 some randomized clinical trials have examined the effectiveness of using CHWs to improve post-discharge outcomes in adult inpatient and outpatient general medicine populations. In a study of 222 Medicaid inpatients randomized to a CHW intervention, Kangovi et al27 found that having at least one 30-day readmission did not differ between the intervention and control groups; however, there was a significant reduction in the number of intervention participants with 2 or more 30-day readmissions. In a randomized clinical trial of 1009 inpatients older than 60 years, Balaban et al28 found that CHW care reduced 30-day readmission rates in intervention participants compared with control participants. Another randomized clinical trial by Kangovi et al29 of 592 primary care outpatients tested a 6-month CHW intervention and demonstrated a reduced likelihood of readmission. Two other randomized clinical trials examining 6-month interventions with CHWs demonstrated no significant difference in hospitalizations.30,31 To determine the effect of CHW care delivery on 30-day readmissions within an ACO population, we conducted a randomized clinical trial to test a 30-day CHW intervention for patients admitted to the internal	Intervention includes usual care<br><br>Components:<br>1.Health coaching<br>2.Psychosocial support,<br>3.Assistance obtaining any needed clinical access or social resources	Yes	No justification or explanation	Justification not specific to individual components

Author, Year	Justification for intervention from publication[a]	Intervention description	Multiple Intervention Comp-onents	Was the intervention developed to respond to community or individual social need(s)?	Justification for specific components
	medicine service in an academic medical center in Boston, Massachusetts.				
Caskey, 2019[81]	The number of children with chronic disease is increasing in the United States, with nearly 25%of children having been diagnosed with at least 1 chronic medical condition.1,2 Minority and low-income children are more likely to have a chronic disease and poorer health outcomes compared with non-Hispanic white children and children with higher income.1 Children with chronic medical conditions consume a disproportionate share of the pediatric health care expenditures in the United States, especially for inpatient care.3 Small-scale studies have demonstrated that an enhanced medical home model can decrease healthcare costs among children with chronic disease.4 Similarly, coordination of care has shown promise in improving care delivery and outcomes among children with asthma.5 However, a hospital-based comprehensive case management program resulted in an increase in cost among medically complex children.6 Further, studies of large-scale care coordination programs for Medicaid and Medicare beneficiaries have demonstrated variable impact on expenditures, with some programs decreasing cost while others appear to have had no impact.7-9 In 2014, the University of Illinois Health and Health Sciences System developed a comprehensive care coordination demonstration project designed to provide services for children and young adults with chronic health conditions living in Chicago, Illinois, who are insured by Medicaid. This program, Coordinated Healthcare for Complex Kids (CHECK),was funded by a Centers for Medicare & Medicaid Services Innovation (CMMI) Award.10 The CHECK model took a broad approach to care coordination and health promotion by addressing social determinants of health, caregiver wellness, and	1. Participants were risk stratified as low, medium, or high risk according to claims-data on healthcare utilization in the 12 months prior to enrollment.<br>2. Participants were contacted by the Coordinated Healthcare for Complex Kids program and completed an initial health assessment, a care plan was created	Yes	Prevalence of social need(s) in the community	Justification not specific to individual components

Author, Year	Justification for intervention from publication[a]	Intervention description	Multiple Intervention Comp-onents	Was the intervention developed to respond to community or individual social need(s)?	Justification for specific components
	mental health needs in addition to chronic disease management.11 The program targeted children and young adults with diagnoses of asthma, diabetes, sickle cell disease, seizure disorder, or prematurity from birth to age 25 years. All participants were enrolled in the traditional, fee-for-service state Medicaid program or a Medicaid managed care organization (MCO) in Illinois. The CHECK program provided access to multiple services, including care coordination delivered by community health workers (CHWs), mental health services delivered by mental health professionals, and disease-specific health education. Community health workers assessed individual and family needs as well as patterns of health care utilization during the year prior to enrollment to determine specific services offered to each family. For example, the family of a child with uncontrolled asthma and multiple emergency department (ED) visits may have received a home visit by a CHW to evaluate potential environmental triggers, review medications, and provide in-depth asthma education, followed by monthly telephone calls to check in on the child and family. The primary aim of the CHECK program was to decrease Medicaid expenditures during a 3-year period by decreasing unnecessary ED visits and hospitalizations. We had a unique opportunity to prospectively randomize participants to receive CHECK vs usual care (UC) to evaluate the program; this provided analytic rigor not available to many large-scale care delivery programs. This report describes an analysis evaluating the effect of the CHECK program on participants' Medicaid expenditures and ED and inpatient utilization				
Cauce, 1994[11]	Despite the seriousness of these problems, homeless street youths often fail to receive services because of a nonresponsive service system ill-prepared to meet their needs (Farrow et al, 1992). They have been described as "victims of piecemeal interventions"	1. Assessment: Intensive case managers spend a significant amount of time with youths, often doing everyday things with them.	Yes	Community advisory board or other community input	Justification not specific to individual components

© 2024 Viswanathan M et al. *JAMA Network Open.*

Author, Year	Justification for intervention from publication[a]	Intervention description	Multiple Intervention Comp-onents	Was the intervention developed to respond to community or individual social need(s)?	Justification for specific components
	(Kurtz, Jarvis, and Kurtz 1991). Homeless youths need intensive services that are integrated into a continuum of care. Furthermore, because homeless youths seldom fit into traditional molds, services must be flexible and forgiving, allowing youths to "try and fail and try again," just as they would be allowed to do within a family context (Kurtz et al 1991, p. 236). Rotheram-Borus and Bradley (1991) noted that an additional barrier to their effective treatment is the limited clinical skills of youth workers at most agencies that serve them. They noted that although many youth workers are talented professionals, few have professional mental health training. A research demonstration project was launched to provide intensive mental health case management to this group in Seattle. This intensive intervention was designed to provide more responsive, individually tailored services that are adolescent centered, culturally sensitive, and community based. The intent was to fully use existing community resources and create more flexibility within the system to meet the needs of this population. The intensive mental health intervention will be described and data from the first 3 months of the follow-up evaluation will be presented here. The larger research demonstration project is referred to as the Seattle Homeless Adolescent Research Project (SHARP); the actual intervention is called Project Passage because it attempts to help homeless youths negotiate the transition into adulthood.	2. Treatment teams: Teams meet with the youths and their support system members to develop and monitor treatment plans. This team approach assures continuity of services and decreases the splitting and manipulation of service providers that often occurs when serving adolescents.<br><br>3. Linkage to appropriate services<br><br>4. Monitoring and tracking youths<br><br>5. Advocacy for a youth's basic entitlements, such as food and housing, medication for mental illness, documentation of learning disabilities so that special education can be provided, and prenatal care.<br><br>6. Counseling/therapeutic relationship: Case managers build relationships with the youth. Although an array of services is made available, the youths choose how, when, where, and if they want a specific service.<br><br>7. Treatment planning: planning begins soon after the initial assessment and is tailored			

© 2024 Viswanathan M et al. *JAMA Network Open.*

Author, Year	Justification for intervention from publication[a]	Intervention description	Multiple Intervention Comp-onents	Was the intervention developed to respond to community or individual social need(s)?	Justification for specific components
		according to the three treatment phases. Plans include: one-to-one counseling, recreational activities with the case manager, and school or day treatment program attendance; therapeutic group work on issues such as sexual victimization and self-esteem, wilderness experiences with the case manager and other youths, community service projects or events; preparation for employment in the community and regular attendance at a school program.<br><br>8. Access to 24-hour crisis service<br><br>9. Flexible funds for purchases and recreational activities to meet needs of the youth			
Cheng, 2008[12]	The Centers for Disease Control and Prevention (CDC) comprehensively reviewed studies evaluating youth violence prevention programs.20 Interventions were categorized and assessed according to target methods and scientifically demonstrated efficacy. The CDC concluded that there are 4 strategies that offer the best hope for prevention, including (1) parent- and family based programs, (2) early childhood home visitation, (3) social-cognitive interventions with youths, and (4) mentoring strategies. Multilevel and multicomponent interventions incorporating these strategies are needed. We developed a violence prevention intervention targeting assault-injured youths who present to the ED and incorporating components of the 4 best-practice strategies	1. Assault-injured youths in the intervention received a mentor who was to meet with them more than or equal to 6 times in the subsequent 2 to 6 months. The mentors picked up youths at their homes and spent time with the youths in activities at their homes or in the community.<br><br>2. During these visits, the mentors and youths completed a 6-session, violence prevention curriculum focusing on skills	Yes	Prevalence of social need(s) in the community	Yes

Author, Year	Justification for intervention from publication[a]	Intervention description	Multiple Intervention Comp-onents	Was the intervention developed to respond to community or individual social need(s)?	Justification for specific components
	described by the CDC, including a social-cognitive theory framework.	building. The curriculum was grounded in social-cognitive theory and included sessions on conflict management and hot buttons, problem-solving, weapon safety, decision-making, and goal setting. The curriculum also included interactive activities, role-playing scenarios, and a pledge to remain nonviolent.<br><br>3. Parents of youths in the intervention were offered 3 home visits from a health educator.<br><br>4. Families received case management services, including discussion of sequelae of assault injuries, assessment of family needs, and facilitated service use. Intervention group participants received this service through the health educator.<br><br>5. Referrals were made for mental health treatment, medical services, addiction treatment, anger management programs, recreational and after-school programs, legal aid, tutoring programs, and other social services as requested by the family. Families received usual ED care and list of community resources.			

© 2024 Viswanathan M et al. *JAMA Network Open.*

Author, Year	Justification for intervention from publication[a]	Intervention description	Multiple Intervention Comp-onents	Was the intervention developed to respond to community or individual social need(s)?	Justification for specific components
Corrigan, 2017[13]	Services for people with serious mental illness have a rich history of including peer-provided interventions.21 These include treatments delivered by peer providers to address the health needs of participants with serious mental illness. Four randomized clinical trials (RCT) showed people who received versions of psychiatric case management services from peers demonstrated the same level of functional and symptom stability as those provided by professional of paraprofessional staff 22,23,24,25 though these findings have to be interpreted cautiously because they fundamentally represent support of the null hypothesis (i.e., no difference between peer and professional case managers). More recently, people with serious mental illness in hospitals receiving peer mentoring had significantly fewer hospitalizations and inpatient days during the nine months of the study.26 For the most part, these studies did not examine benefits on health needs per se, though they frequently examined overall improvements in quality of life. Moreover, the peer intervention was not informed by service guidelines that have evolved for patient navigators.14, 27 Hence, the CBPR team conducting the earlier qualitative study12 used study results to adapt navigator guidelines for the needs and priorities of African Americans with serious mental illness who were homeless.28 Here, we report findings from a subsequent RCT comparing the effectiveness of this peer navigator program (PNP) to treatment as usual (TAU). We expected to show people participating in PNP would report improvements in both psychiatric and physical health which would correspond with a better sense of recovery and improved quality of life.	1. Peer navigators used motivation interviewing to assess needs and create goals with participants.<br><br>2. Peer navigators used a manual adapted from the National Cancer Institute and worked to assist clients with area resources.<br><br>3. Peer navigators worked as advocates, health educators, liaisons between participants and their health providers, and helped resolved problems between the participant and the healthcare system.	Yes	Community advisory board or other community input, Prevalence of social need(s) in the community	Justification not specific to individual components
Counsell, 2007[14]	The Geriatric Resources for Assessment and Care of Elders (GRACE) model of primary care was developed specifically to improve the quality of care for low-income seniors. The GRACE model builds on	1. Geriatric Resources for Assessment and Care of Elders support team met with the patient in the home to conduct	Yes	Prevalence of social need(s) in the community	Justification not specific to individual components

© 2024 Viswanathan M et al. *JAMA Network Open.*

Author, Year	Justification for intervention from publication[a]	Intervention description	Multiple Intervention Comp-onents	Was the intervention developed to respond to community or individual social need(s)?	Justification for specific components
	lessons learned from prior efforts to improve the care of older adults through multidimensional assessment. Prior reviews of this literature suggest that time-limited and site-specific geriatric consultation has limited impact on the process and outcomes of care.6-8 In addition, the inpatient Acute Care for Elders (ACE) model was shown to be a cost-effective design to improve outcomes in hospitalized older patients by providing a geriatrics interdisciplinary team that integrates and enhances care delivered by the hospital attending physician.9-12 Building on the ACE model, we designed the GRACE intervention to improve the longitudinal integration of geriatric and primary care services across the continuum of care and thereby improve the likelihood that older adults receive recommended care.13 Unique features of the GRACE intervention compared with prior studies of home-based integrated geriatric care14-19 include the following: in-home assessment and care management provided by a nurse practitioner and social worker team; extensive use of specific care protocols for evaluation and management of common geriatric conditions; utilization of an integrated electronic medical record and a web-based care management tracking tool; and integration with affiliated pharmacy, mental health, home health, and community-based and inpatient geriatric care services.13	an initial comprehensive geriatric assessment.<br><br>2. Work with the larger interdisciplinary team to develop an individualized care plan; then meet face-to-face with the patient's primary care physician to discuss and modify the plan.<br><br>3. The support team implements the plan consistent with the patient's goals through face-to-face (usually in the patient's home and occasionally in the office, hospital, or nursing home) and telephone contacts with patients, family members or caregivers, and healthcare professionals and the implementation of specific protocols that include health monitoring, education, recommendations for specific health programs, and linking to existing health system and community-based services, all as an integration across the continuum of care.			
Cox, 1998[15]	One intervention that has been applied to severely and multiply disabled persons, and that can incorporate or coordinate these service components, is case management (CM). In 1990, when this project was proposed, there existed in the literature only a few descriptions of case management as applied to alcohol and substance abuse treatment, none of which were controlled studies(Birchmore-Timney and Graham 1989; McCarty et al 1991a; Steinbach, 1982;	Long-term, open-ended, outreach-oriented service focused on system advocacy and linkage activities.<br>Provision of service was not conditional on client behavior and no requirement for sobriety.<br>Program coordinated clinical	Yes	No justification or explanation	Justification not specific to individual components

© 2024 Viswanathan M et al. *JAMA Network Open.*

Author, Year	Justification for intervention from publication[a]	Intervention description	Multiple Intervention Comp-onents	Was the intervention developed to respond to community or individual social need(s)?	Justification for specific components
	Willenbring et al 1990, 1991). In the first round of Research Demonstration Projects from the National Institute on Alcohol Abuse and Alcoholism (NIAAA), four of the nine projects included CM (Argeriou and McCarty 1990). In the second round of Research Demonstration Projects all of the projects included some form of CM, and most included some form of random assignment (N IAAA 1991). Increased interest in CM is reflected in a growing number of descriptive or prescriptive articles(Abel and Cummings 1993; Bonham 1992; Conrad et al 1993; Sullivan et al 1994; Willenbring 1994), and well-controlled studies are beginning to appear in print (Jerrell and Ridgely, 1995; Martin and Scarpitti 1993), including several from the NIAAA-funded Research Demonstration Projects on homeless substance-abusing persons of which this project was one (Stahler 1995). Results from these studies are mixed. In the work most relevant to the current study, the volume edited by Stahler (1995), several projects did report favorable outcomes due to case management, but most did not. Nearly all the reports indicated an overall improvement across all treatment conditions.	care and attempted to strengthen clients' social and personal skills and encourage increased autonomy. Clients helped determine which of their needs and interests would be served first. Caseloads averaged 15 clients per case manager. Generic treatment goals were to stabilize financial conditions and housing status and encourage individuals to reduce substance use.			
Dixon, 2009[16]	Although previous work has evaluated correlates of enhanced retention in psychiatric treatment, few studies have specified interventions directly targeting continuity of care. Emerging evidence suggests that brief, low intensity case management interventions are effective in bridging the gap between inpatient and outpatient treatment (13,14).	1. The brief CTI clinician receives a referral and immediately meets with the patient to assess individual needs and barriers to outpatient care.<br><br>2. The clinician builds rapport, develops individualized treatment goals, identifies barriers to treatment, and establishes a case management plan. There are nine possible target areas for clinical focus. The focus areas are determined	Yes	No justification or explanation	Justification not specific to individual components

Author, Year	Justification for intervention from publication[a]	Intervention description	Multiple Intervention Comp-onents	Was the intervention developed to respond to community or individual social need(s)?	Justification for specific components
		by the initial assessment and ongoing reappraisal of the patient's needs.<br><br>3. After discharge, the brief CTI clinician maintains a high level of patient contact, conducting home visits, accompanying the patient to initial appointments, and providing emotional and practical support for the patient and family.			
Duncan, 2020[17]	Our goal was to develop, implement, and evaluate a comprehensive, evidence-based, post-acute care model in real-world practice. Our care model uses elements of transitional care management and ESD evaluated in our prior work with the TRAnsition Coaching for Stroke (TRACS) model that demonstrated reduced readmissions [39]. The intervention was designed to be consistent with the Centers for Medicare and Medicaid Services (CMS) care and reimbursement models for transitional care management [40].	1. Contact after hospital discharge to address medication reconciliation, assessment of new stroke symptoms, receipt of home health or outpatient services, and scheduling of follow-up appointments with primary care physician and Comprehensive Post-Acute Stroke Services providers<br><br>2. Clinic visit with series of standardized assessments<br><br>3. An individualized patient electronic care plan (Comprehensive Post-Acute Stroke Services – Care Plan) that includes coordination and coaching to incorporate recommended strategies, referrals to primary care, specialty care, rehabilitation services, and community services, and communication	Yes	Community advisory board or other community input	Justification not specific to individual components

© 2024 Viswanathan M et al. *JAMA Network Open.*

Author, Year	Justification for intervention from publication[a]	Intervention description	Multiple Intervention Comp-onents	Was the intervention developed to respond to community or individual social need(s)?	Justification for specific components
		with primary care, pharmacy, home health, and rehabilitation services<br><br>4. Evaluation of adherence to individualized plan			
Eismann, 2022[18]	Parent Connext is a positive parenting and prevention program that was designed within the pediatric primary care setting in response to the American Academy of Pediatrics recommendations above (Lott 2020). Parent Connext integrates both screening and surveillance for parenting and family psychosocial concerns as well as co-located parent coaching services within pediatric primary care. Pediatricians screen families for parenting and family psychosocial concerns at targeted well-child visits from birth to 6 years of age and routinely surveil for these concerns at all other patient visits. They use motivational interviewing skills to discuss concerns with families and connect them with resources. A similar screening model has previously been shown to reduce child protective services reports and harsh punishment by parents (Dubowitz, Feigelman, Lane, and Kim 2009). Parent coaching is a collaborative one-on-one process intended to guide, equip, and empower parents toward achieving their parenting-related goals. Parenting Specialists from a community-based organization with graduate level education work with parents on-site at the pediatric practice to identify and employ solution-focused strategies for their parenting concerns with the goal of promoting child social−emotional and behavioral health through strengthened positive parenting and family functioning. Parent coaching services are available to all families within the practice, regardless of the child's age. By offering parenting services on-site, parents can receive nearly immediate access to individualized support within a familiar environment. When a referral	1. Eliciting and understanding concerns of care giver<br><br>2. Envisioning desired parenting goals<br><br>3. Providing education on child development, parenting guidance and resources, self-care guidance, resource navigation and referral, and crisis support as appropriate<br><br>4. Collaboratively identifying intentional daily actions to support progress<br><br>5. Affirming and building on strengths as the foundation for change<br><br>6. Ongoing monitoring	Yes	Other; the intervention was designed within the pediatric primary care setting in response to the American Academy of Pediatrics recommendations.	Justification not specific to individual components

© 2024 Viswanathan M et al. *JAMA Network Open.*

Author, Year	Justification for intervention from publication[a]	Intervention description	Multiple Intervention Comp-onents	Was the intervention developed to respond to community or individual social need(s)?	Justification for specific components
	for more extensive evaluation or intervention is warranted, Parenting Specialists can serve as a bridge to connect parents with other services, such as mental health counseling. This paper is the first to describe the Parent Connext program and explores the feasibility and acceptability of its integration within 11 pediatric primary care practices.				
Ell, 2017[19]	Public health safety-net organizations, such as LAC-DHS, are configuring the delivery of behavioral health integration into their care model. Among low-income patients, depression is a common mental health disorder. Depressive disorder among patients with chronic illness can amplify patient depression relapse, recurrence, morbidity, mortality, while negatively affecting patient self-care management [10–13]. Self-care among low-income patients could also be affected by cultural beliefs, literacy, language and financial barriers that often exceed provider skills in patient communication [14]. To accelerate LACDHS healthcare improvement, the A Helping Hand (AHH) randomized clinical trial (see the trial design article) [15], which tested the comparative effectiveness of integrating community-based, promotora-led psychoeducational sessions (6 weekly psychoeducational sessions plus 3 monthly boosters) aimed at reducing the burden of depression and chronic medical illnesses on patients, families, and care providers.	1. Spanish speaking culturally concordant promotoras met with patients in person at their home or preferred setting to build self-care management skills and action planning to address problem list over 6 sessions<br><br>2. Promotoras provided 3 monthly booster sessions to reinforce education<br><br>3. Promotoras provided written materials, modeled problem-solving, and offered opportunities for patients to practice the problem-solving skills to activate self-care and patient communication with their medical provider about treatment access, healthcare concerns, and self-care management.<br><br>4. Promotoras addressed problems that arose such as family conflict, financial strain, employment issues and immigration concerns and referred to care providers or community resources as	Yes	Community advisory board or other community input	Justification not specific to individual components

Author, Year	Justification for intervention from publication[a]	Intervention description	Multiple Intervention Comp-onents	Was the intervention developed to respond to community or individual social need(s)?	Justification for specific components
		needed.<br><br>5. Promotoras documented patient progress at each intervention session<br><br>6. Promotoras referred patients to care providers and community resources for medical or psychosocial concerns			
Ferrer, 2019[20]	Given primary care's central role in managing diet-related chronic illness, our study objective was to test whether a novel collaboration between a primary care practice and a municipal food bank could improve food security while also achieving better control of type-2 diabetes mellitus.	1. Biweekly allotment of fresh produce and other healthy vegetables delivered by a Mobile Mercado truck from the San Antonio Food Bank to the clinical site, approximately 10 pounds of fresh produce and 10 pounds of canned food, including beans, vegetables, and fish or chicken.<br><br>2. Teaching by a registered dietician from the Food Bank regarding current nutritional guidelines for people with diabetes mellitus. Food Bank also screened patients to assess eligibility for other assistance programs.<br><br>3. Up to 3 home visits by a promotor affiliated with the practice. These community health workers used a rubric, the six-piece puzzle of food, physical activity, medicines, self-care, numbers (HbA1c,	Yes	Prevalence of social need(s) in the community	Justification not specific to individual components

Author, Year	Justification for intervention from publication[a]	Intervention description	Multiple Intervention Comp-onents	Was the intervention developed to respond to community or individual social need(s)?	Justification for specific components
		blood pressure, cholesterol), and trust (in healthcare team and personal relationships), to frame self-management goals.			
Finkelstein, 2020[21]	Since being profiled in Atul Gawande's seminal New Yorker article, "The Hot Spotters",10 the Camden Coalition of Healthcare Provider's (the Coalition's) program has been the flagship example of a promising super-utilizer program. The Coalition's Camden Core Model uses real-time data on hospital admissions to identify super-utilizer patients, an approach referred to as "hotspotting." Focusing on patients with chronic conditions and complex needs, and starting with the premise that the standard system is difficult to navigate for these patients, the program uses a high-touch, face-to-face care model to engage patients and connect them to appropriate medical care, government benefits, and community services, with the aim of improving health and reducing unnecessary utilization. The program has been heralded as a promising, data-driven, relationship-based, intensive care management program for super-utilizers, and federal funding has expanded versions of the model to other cities.7–16 To date, however, the only evidence of its impact is a pre-post analysis of the healthcare spending of 36 participants17 and an evaluation of four expansion sites comparing 149 program participants with propensity-score matched controls.18 More broadly, there are a number of promising observational studies of other super-utilizer programs.12,17,19–21 However, regression to the mean—the tendency for patients selected as exceptionally high-cost at a moment in time to move closer to average cost over time—may bias observational studies of super-utilizer programs towards spurious results.22,23 Although there is limited rigorous evidence of the effectiveness of super-utilizer programs, several randomized trials	1. Inpatient enrollment conducted by care team.<br><br>2. On discharge to home, multidisciplinary team conducted an initial home visit to assess needs and create health goals and a plan to meet the goals.<br><br>3. Intensive case management included continued home visits, scheduling and accompanying participants to initial primary and specialty care visits, coordination of follow-up care and medication management, conducting blood pressure and blood sugar checks, coaching participants in disease-specific self-care.<br><br>4. Case management also included helping participants apply for social services and appropriate behavioral health programs, accompanying participants to appointments as needed, arranging transportation as needed.	Yes	Community advisory board or other community input, Prevalence of social need(s) in the community	Justification not specific to individual components

© 2024 Viswanathan M et al. *JAMA Network Open.*

Author, Year	Justification for intervention from publication[a]	Intervention description	Multiple Intervention Comp-onents	Was the intervention developed to respond to community or individual social need(s)?	Justification for specific components
	of care transition programs—which, like the Camden Core Model, start with patients in the hospital and work with them post-discharge—find substantially reduced readmissions.24–29 However, the Camden Core Model targets a much more heterogeneous population with greater social and medical complexity and substantially higher healthcare utilization. Therefore, the Coalition partnered with the investigators to design a prospective randomized evaluation of this nationally recognized program.				
Flores, 2018[80]	Despite the long-standing marked insurance disparity for Latino children, not enough is known about what interventions are most effective for insuring them, particularly those eligible for Medicaid or the Children's Health Insurance Program (CHIP). There has only been one prior randomized controlled trial of an intervention specifically designed to insure Latino children.5 That study, conducted by our team, showed that employing community health workers was more effective than traditional outreach and enrollment methods in obtaining coverage for uninsured Latino children. But it did not differentiate between children eligible for Medicaid or CHIP and noneligible children; evaluated only four insurance-related outcomes; and did not examine healthcare access, care quality, satisfaction with care, or costs.	Parent mentors provided the following eight services to intervention children and families: teaching about types of insurance programs and application processes, furnishing information and assistance on meeting Medicaid or CHIP eligibility requirements, helping parents complete and submit children's insurance applications, expediting final coverage decisions by making early and frequent contacts with Medicaid or CHIP personnel, acting as family advocates by liaising between families and Medicaid or CHIP agencies, contacting Medicaid or CHIP officials to resolve situations in which children were inappropriately deemed ineligible or had coverage inappropriately discontinued, helping parents complete and submit applications for coverage renewal, and educating parents about renewing Medicaid or	No	Prevalence of social need(s) in the community	NA, single intervention

Author, Year	Justification for intervention from publication[a]	Intervention description	Multiple Intervention Comp-onents	Was the intervention developed to respond to community or individual social need(s)?	Justification for specific components
		CHIP coverage or reapplying after losing it.			
Gottleib, 2020[22]	Despite considerable diversity in existing pediatric clinical activities aimed at identifying and reducing social risk, little research has rigorously examined program effects or assessed the feasibility of scaling programs across pediatrics clinical settings.47 In this study, we compare the effectiveness of 2 social risk–related interventions that differ in intensity and potential scalability: in-person social services navigation assistance offered for up to 3 months by a trained volunteer vs standardized, written information about community and government social services resources.	1. Dyads randomized to receive in-person navigation services received them either immediately after randomization or via a telephone or in-person follow-up appointment at a different time. During these visits, navigators provided written information about resources related to the caregivers prioritized social risk factors.<br><br>2. Additionally, navigators helped to schedule appointments, complete forms, or provide other social services related counseling and assistance.<br><br>3. Following the initial visit, caregivers in the in-person assistance group were contacted by navigators by telephone, text message, or email every 2 weeks for 3 months until identified needs were met or caregivers declined further assistance, whichever came first.	Yes	No justification or explanation	Justification not specific to individual components
Gottlieb, 2016[23]	In line with these recommendations, a number of social interventions have emerged in pediatric clinical settings.21-25 Research on these efforts has primarily focused on assessing intervention processes or reductions in social needs. Such studies21-24,26,27	1.Navigators used algorithms to provide targeted information related to community, hospital, or government resources	Yes	No justification or explanation	Justification not specific to individual components

© 2024 Viswanathan M et al. *JAMA Network Open.*

Author, Year	Justification for intervention from publication[a]	Intervention description	Multiple Intervention Comp-onents	Was the intervention developed to respond to community or individual social need(s)?	Justification for specific components
	have demonstrated that social screening and referrals can increase connections with community resources and decrease social needs. However, no studies have examined whether social interventions influence children's health, to our knowledge. We address this gap by evaluating the effect on family social needs and parent-reported child global health status of an in-person screening and case management intervention targeting pediatric social needs vs an active control condition providing written resource information.	addressing needs that participants had prioritized.<br>2.Follow-up meetings were offered every 2 weeks for up to 3 months, until identified needs were met, or when caregivers declined further assistance.			
Guevara, 2020[24]	Based on these observations, The Reach Out and Read (ROR) Program was established to promote parent-child shared reading activity among poor at-risk children at pediatric well-child visits.15 Evaluations of ROR have shown beneficial effects on reading activity and language outcomes.16–18 These effects translate into an increase of 1 day/week of parent-child reading activity and a 4- to 9-point increase in expressive and receptive language scores, respectively.17 For these reasons, the American Academy of Pediatrics has recommended that pediatric clinicians promote literacy development beginning in infancy.19 Despite the encouraging results that ROR has shown to date, poor children who participate in the program still demonstrate language scores that are 6-15 points lower than national averages, and 37%-45% are still at high risk for reading problems prior to kindergarten.17,20 This may in part be the result of delays in promoting parent-child verbal interactions until children are 6 months of age or older. There may be reason to suspect that children exposed to richer and more intensive parent-child verbal interactions prior to 6 months of age may achieve greater language functioning and better outcomes.9,21,22	1. Reading promotion<br><br>2. Board books at well-child visits<br><br>3. Weekly text messages to promote literacy.	Yes	Other, Pre-existing intervention applied to younger children	Justification not specific to individual components

© 2024 Viswanathan M et al. *JAMA Network Open.*

Author, Year	Justification for intervention from publication[a]	Intervention description	Multiple Intervention Comp-onents	Was the intervention developed to respond to community or individual social need(s)?	Justification for specific components
Hannan, 2016[25]	Interventions for follow-up care with postpartum low-income first-time mothers using mobile technology (cell phone use and text messaging) by nurse practitioners (NPs) is a simple yet potentially effective method to improve care and maternal/infant health outcomes while controlling costs. This could be especially important in this group of mothers, many without health insurance, have difficulty accessing the healthcare system, financial struggles, and language barriers. Recently, interventions using mobile technology (cell phone use and text messaging) are increasingly being used in other populations for health promotion and assistance with chronic health problems.16-19 However, interventions are lacking using 2-way communication with healthcare providers via cell phone contact and texting with low-income first-time mothers.20 Such an intervention delivered by NP providers who have specialized in the care of mothers and infants hold potential to improve both maternal and infant health and to decrease healthcare charges in this vulnerable group. Therefore, the purpose of this study was to test the effects of an NP 2-way communication cell phone and texting follow-up intervention for the first 6 months postbirth in low-income first-time mothers and their healthy full-term infants.	1. 2-way cell phone contact and texting beginning on the third posthospital discharge day and then on days 7, 14, 21, and monthly to month 6 to assess health problems or concerns regarding the infant. Mothers also able to contact the NPs by cell phone or texting with any infant health concerns. The NPs documented the mother's concern and the care provided using the protocol in a clinical interaction log.<br><br>2. If a healthcare concern was stated, the NP used the AAP Pediatric Telephone Protocol to implement care. If the mother voiced a serious infant complaint such as fever, excessive crying, vomiting, lethargy, or seizure-like activity, the mother was instructed to contact the 911 emergency systems.<br><br>3. If there was no health concern, the NPs documented the mother's reply in the log.	Yes	Prevalence of social need(s) in the community	Justification not specific to individual components
Heisler, 2022[26]	One effective approach to reach vulnerable individuals facing barriers to accessing care is outreach and support from community health workers (CHWs). Defined as trained frontline health workers who are trusted members of – or closely connected to – the population served, CHWs share characteristics such as culture, language, or community with those they serve [15].... In randomized controlled trials,	CHWs contacted individuals by phone or in person to offer assistance, which included:<br><br>1. Conducting an initial comprehensive health, behavioral, and social needs assessment	Yes	Community advisory board or other community input	Justification not specific to individual components

Author, Year	Justification for intervention from publication[a]	Intervention description	Multiple Intervention Comp-onents	Was the intervention developed to respond to community or individual social need(s)?	Justification for specific components
	CHWs have improved clinical outcomes in areas such as diabetes [16–22], heart disease and hypertension [23–26], cancer screenings and literacy [27–29], readmission rates [30–32], and mental health [33–37]. Components of CHW programs supported by strong evidence include providing chronic disease care services; inclusion in team-based care models; certification based on core competencies; certification to establish standards for specialty areas; supervision by healthcare professionals; and reimbursement of services by Medicaid payment [38]. Some efficacy trials have shown that well-designed CHW programs can decrease acute care utilization and result in cost savings [17,33,39–41]. Despite this growing body of evidence, research on CHW programs that operate sustainably in real-life settings is still limited. The impact of most CHW initiatives remains limited by their dependence on short-term grants. For any model to be sustainable, a payer must cover the costs of CHW services, or alternatively, a fee-for-service billing code for CHW services must be established. Our study addresses this gap in the literature by evaluating a sustainable CHW program that was developed and implemented by Medicaid health plans.	2. Developing an individualized action plan<br><br>3. Linking members to necessary services and working with neighborhood-based healthcare and social services organizations to address each individual's unique needs<br><br>4. Providing follow-up support as needed			
Henschen, 2021[27]	We created the Complex High Admission Management Program (CHAMP) as a quality improvement initiative to improve inpatient and outpatient care and reduce inpatient readmissions of patients frequently admitted to our hospital. The CHAMP multidisciplinary team works to foster longitudinal relationships with patients who suffer from systematic discontinuity. A pilot pre-post analysis of CHAMP observed reductions in readmission;13 however, results may have been confounded by the aforementioned tendency for utilization to decline over time.10 In this study, we conducted a randomized trial	1. Upon enrolling a new patient, the Complex High Admission Management Program team conducted an in-depth psychosocial assessment, helped the patient set specific health-related goals, and attempted to develop a therapeutic relationship.<br><br>2. The team collaborated with patients to write a comprehensive care plan.	Yes	No justification or explanation	Justification not specific to individual components

Author, Year	Justification for intervention from publication[a]	Intervention description	Multiple Intervention Comp-onents	Was the intervention developed to respond to community or individual social need(s)?	Justification for specific components
	of CHAMP compared with usual care to accurately assess the program's effect on hospital readmissions.	Components of the care plan included providing medical history and personal details that enhance each patient's individuality; care recommendations for the ED, hospital, and community setting; and contact information for key team members.<br><br>3. After enrollment, when an intervention patient arrived in the ED, an intervention team member received an automatic page, enabling them to contact the patient and ED team during business hours to help guide care. If hospitalized, one or more team members visited each patient daily; intervention patients were directly cared for by hospital staff. Intervention team members created and maintained the comprehensive care plan, coordinated follow-up care, and connected patients to existing community resources.<br><br>4. After discharge, patients were scheduled to follow up with the intervention team in an outpatient clinic embedded in existing transitional care clinic space. Social workers frequently made outreach to patients beyond the inpatient and primary care setting.			

Author, Year	Justification for intervention from publication[a]	Intervention description	Multiple Intervention Comp-onents	Was the intervention developed to respond to community or individual social need(s)?	Justification for specific components
Herman, 2000[28]	Discontinuity between institutional and community treatment is a critical problem (Dorwart and Hoover 1994; Olfson et al 1998) that has contributed to a range of negative outcomes among persons with severe mental illness after institutional care. CTI is a time-limited intervention that overlaps the period before and after discharge from the institution to the community. A key aspect of CTI is that the post-discharge phase of the intervention is delivered by staff who have established relationships with patients during their institutional stay. CTI shares with long-term assertive community treatment models (Lehman et al 1997) a focus on stabilizing patients in the community through "in vivo" development of independent living skills and by building effective support networks in the community. The emphasis, however, is on maintaining continuity of care during a critical period of transition, whereas primary responsibility is gradually passed to the existing supports in the community.	1. Strengthen the individual's long-term ties to formal and informal supports including services, family, and friends.<br><br>2. Providing emotional and practical support during the critical time of transition.	Yes	Other, CTI is based on the premise that a well-timed intervention can help individuals leaving a shelter develop relationships with the community providers who can offer ongoing care	Justification not specific to individual components
High, 2000[79]	Several studies have evaluated the efficacy of litarcy promoting anticipatory guidance delivered by pediatric primary care providers as part of well-child care. In 1991, Needlman and his colleagues6 indicated that family literacy orientation could be increased by a simple intervention in which pediatricians distributed children's books to children of low-income parents at their health maintenance visits. We initially conducted a similar intervention study with multicultural, low-income families (100 intervention, 51 historical controls).7 At all scheduled well-child visits in a hospital clinic setting, primary care providers distributed children's books and educlatoin materials to parents of 6- to 38-month-old children in the intervention group. This study established the concept of Child Centered Literacy Orientation (CCLO), a measure of family's ability and willingness to engage in literacy-promoting activities with young	At this initial visit and at all subsequent well-child visits, pediatricians gave children in the intervention group an age-appropriate children's board book; an age-specific handout explaining how children can benefit from, enjoy, and interact with books; and literacy-promoting anticipatory guidance.	No	No justification or explanation	NA, single intervention

© 2024 Viswanathan M et al. *JAMA Network Open.*

Author, Year	Justification for intervention from publication[a]	Intervention description	Multiple Intervention Comp-onents	Was the intervention developed to respond to community or individual social need(s)?	Justification for specific components
	children. CCLO considered present if parents mentioned reading in answer to open-ended questions about their child's favorite activities, or their favorite things to do together, or if they usually shared books at bedtime. We found that 33% of parents had CCLA in the historical control group compared with 69% of families who received the intervention.				
Hilgeman, 2014[29]	Empirical studies examining the needs, barriers, and availability of healthcare services for military veterans who are not utilizing the VA healthcare system are not widely available. In addition, much-needed outreach interventions require empirical evidence in their favor for funders to consider their continuation or expansion. This study's enrollment intervention directly addresses these gaps. If the success of this enrollment intervention is supported, then this intervention could augment other rural health services within VHA (eg, CARE, MHICM, Home-Based PrimaryCare or Mobile Primary Care teams) and analogous services to augment health service access for nonveteran populations.	1. Motivational interviewing<br><br>2. Education<br><br>3. Patient navigation	Yes	No justification or explanation	Justification not specific to individual components
Horwitz, 2005[30]	Given the recent large increases in nondiscretionary government spending and rapidly escalating healthcare costs, significant declines in the number of uninsured Americans in the next few years are unlikely. One proposed interim policy is to increase government funding to low-cost primary care providers (ie, FQHCs) and link the uninsured to these providers. Yet, there is only limited information about the potential for these policies to change utilization patterns, improve health, or increase the efficiency of the healthcare system. By examining interventions that link uninsured patients who use the healthcare system to primary care providers, we can develop some information about the effects of such a policy, at least for one population likely to benefit from additional funds to low-cost providers.	Health promotion advocates identified participants in EDs; once enrolled, Health Promotion Advocates assisted enrolled patients in choosing one of four primary care providers, provided patients with a study identification card and brochure, and faxed all information to a case worker at the selected primary care site. Upon receipt of the referrals, case managers at each site attempted to contact and schedule appointments on-site for these patients.	No	No justification or explanation	NA, single intervention

Author, Year	Justification for intervention from publication[a]	Intervention description	Multiple Intervention Comp-onents	Was the intervention developed to respond to community or individual social need(s)?	Justification for specific components
Johnson, 2022[31]	The Veterans Health Administration (VHA) has standards for women's healthcare which build on the Patient Aligned Care Team (PACT) model to address women Veterans' unique needs.5 The PACT model is an interdisciplinary team-based approach similar to patient-centered medical homes which includes medical social workers,6 embedded behavioral health providers,7 and, most recently, peer support specialists. Peers are Veterans in recovery from a behavioral health concern with specialized training to support other Veterans.8 Peers working in PACT teams are well positioned to provide navigation and social support for broad, nondiagnostic concerns such as stress compared to clinical providers who largely address medical or psychiatric diagnostic concerns and social workers who address identified social needs.<br>Personalized Support for Progress (PSP) is a patient-centered lay-delivered intervention co-created with patients.14,15 PSP uses a prioritization tool to help patients identify their priorities, supports personalized planning, and provides emotional and pragmatic support.14,15 Non-Veteran women with depression and unmet social needs in an obstetrics and gynecology practice who received PSP demonstrated high satisfaction and reductions in depression compared to those who received tailored referrals.14 Based on our clinical experiences with women Veterans and the literature about women Veterans' needs and barriers to care, PSP was translated to VHA. The PSP prioritization tool is well suited for the competing demands16 that contribute to stress. Furthermore, the social and pragmatic support fit well with peers' role17 and was ideally suited to address women Veterans' stressors. Thus, we hypothesized PSP delivered by a peer would meet the needs of Veterans in a VHA Women's Wellness Center with high stress.	A prioritization tool asking participants to sort cards reflecting biopsychosocial concerns (e.g., Getting Basic Things I Need and Assistance with Legal Needs) into piles reflecting Biggest CHALLENGE for Me, Less of a CHALLENGE for me, and Not a CHALLENGE for Me. The peer then helped Veterans complete a structured personalized plan and provided flexible follow-up for practical and emotional support.	No	No justification or explanation	NA, single intervention

© 2024 Viswanathan M et al. *JAMA Network Open.*

Author, Year	Justification for intervention from publication[a]	Intervention description	Multiple Intervention Comp-onents	Was the intervention developed to respond to community or individual social need(s)?	Justification for specific components
Kangovi, 2014[32]	There is evidence that CHWs can improve outcomes for patients with chronic conditions 18-23 increase linkages to primary care after emergency visits for hypertension,24 and reduce hospital use for children with asthma.25 This evidence has prompted increased use of CHWs in new models of care, such as the Oregon Coordinated Care Organization.26 However, integration of CHWs into the healthcare workforce has been hampered by 3 factors. First, CHW interventions—including hiring supervision, and work practices—are often poorly standardized and difficult for health systems to reproduce. Second ,most published CHW interventions are disease specific, limiting generalizability to a growing patient population with multiple chronic diseases. Third, according to the Agency for Healthcare Research and Quality,27 many studies of CHW interventions within the United States are of low methodologic quality. Our objective was to create a standardized, exportable CHW model—Individualized Management for Patient-Centered Targets (IMPaCT)—and conduct a randomized clinical trial to test its effect on posthospital outcomes among low-income, uninsured, or Medicaid patients hospitalized with varied conditions.	1. CHWs helped inpatients set goals for recovery and then worked with patients to create an individualized action plan for achieving each of these patient-centered goals.<br><br>2. CHWs served as liaisons between patients and the inpatient care team, explaining patients' goals to the team and ensuring that patients could execute the team discharge instructions.<br><br>3. During and after hospitalization, CHWs provided tailored support based on patients' goals using telephone calls, text messages, and visits.<br><br>4. CHWs connected patients to primary care: they coached patients to schedule and attend appointments and offered to accompany patients to the first posthospital appointment.	Yes	Community advisory board or other community input, Prevalence of social need(s) in the community	Justification not specific to individual components
Kangovi, 2017[33]	A large body of literature suggests that community health workers (CHWs), trained laypeople who share socioeconomic background with patients, can effectively improve chronic disease outcomes.5–10 However, with few exceptions,11 many previous CHW interventions have been disease-specific,10 focusing, for instance, on asthma self-management, diabetes education, or cervical cancer screening. This disease specific approach, likely a consequence of disease-based grant funding, can cause fragmentation for patients with multiple chronic conditions.12 The study	1. Action planning: CHWs and participants CHWs use a semi-structured interview to create patient-driven action plans to meet the goals of one of their chronic conditions.<br><br>2. Tailored support: CHWs helped patients to execute their action plans through tailored support, largely focusing on	Yes	Community advisory board or other community input	Justification not specific to individual components

Author, Year	Justification for intervention from publication[a]	Intervention description	Multiple Intervention Comp-onents	Was the intervention developed to respond to community or individual social need(s)?	Justification for specific components
	team created IMPaCT (Individualized Management for Patient-Centered Targets),12–15 a CHW intervention that can be applied across diseases to address “upstream” socioeconomic and behavioral barriers. The intervention was designed by a community–academic–health system working group to use qualitative participatory action research with high-risk patients. In a previous randomized clinical trial of hospitalized patients with a variety of diseases,15 this intervention improved posthospital access to primary care, mental health, and quality of care while decreasing recurrent hospital readmission. IMPaCT has been adapted for various patient populations, including outpatients with multiple chronic conditions.14	upstream socio-behavioral issues.<br><br>3. Connection with long-term support: CHWs led a weekly patient support group intended to create social networks among patients who could support each other even after the intensive 6-month CHW support ended.			
Kangovi, 2018[34]	Individualized Management for Patient-Centered Targets (IMPaCT) is a standardized intervention in which CHWs provide tailored social support, navigation, and advocacy to help low-income patients achieve health goals. Two previous single-center randomized clinical trials have studied IMPaCT.15,16 The first trial, involving hospitalized patients, demonstrated that IMPaCT improved access to primary care, mental health, and patient activation and quality while reducing 30-day readmissions.9 The second trial, involving outpatients with multiple chronic conditions, demonstrated that the intervention improved chronic disease control glycosylated hemoglobin (HbA1c), body mass index (BMI), cigarettes per day (CPD), mental health, and quality of care while reducing hospitalization.6 In this trial, we tested the scalability and effectiveness of IMPaCT in 3 primary care settings important in the care of low-income patients: a Veterans Affairs (VA) medical center, a federally qualified health center, and an academic family practice clinic.	1. CHWs used a semi-structured interview guide to assess determinants of health and conduct goal setting, including tailored action plans to achieve goals.<br><br>2. CHWs provided 6 months of hands-on, tailored support including coaching, social support, advocacy, and navigation to help patients achieve their action plans.<br><br>3. CHWs helped patients identify long-term supports (neighbors, family members, church, support groups).	Yes	Community advisory board or other community input, Prevalence of social need(s) in the community	Justification not specific to individual components

© 2024 Viswanathan M et al. *JAMA Network Open.*

Author, Year	Justification for intervention from publication[a]	Intervention description	Multiple Intervention Comp-onents	Was the intervention developed to respond to community or individual social need(s)?	Justification for specific components
Kelley, 2020[35]	The most studied intervention among ED frequent users is case management (11). However, a recent comprehensive review of such programs concluded that there is a lack of high-quality evidence to recommend specific interventions (12). Most studies lacked a well-described control group to guard against regression to the mean, which biases toward positive outcomes (13).<br>The highest-quality research available includes three randomized controlled trials (RCTs) (14–16). Spillane et al (14) randomly assigned 66 frequent ED users (defined as more than 10 ED visits per year) to a control group vs intervention with individualized care plans disseminated to ED personnel, multidisciplinary case conferences, and social work or psychiatric evaluation. No difference in ED utilization was found and no cost studies were performed. Shumway et al (15) randomly assigned 252 frequent ED users presenting with ''psychosocial problems'' defined as barriers in housing, medical care, substance abuse, mental health, or financial entitlements to control vs 2-year case management provided by Master's-level psychiatric social workers. Statistically and clinically significant reductions in ED usage occurred; however, there was no reduction in hospital costs. Seaberg et al (16) randomly assigned 282 frequent ED users to control vs. patient navigators who reviewed diagnoses and prescriptions, arranged appointments and transportation, and identified community resources. An 8.9% net reduction in ED visits and 9.1% net reduction in hospital costs occurred. However, limitations included: only 45% of patients had 12-month follow-up, costs were analyzed for only one of 3 hospital systems, and primary care usage was determined from phone surveys relying on patient recall. Given the variability in interventions and results, there is a need for further delineation and testing of quality interventions.	1. Scheduling PCP Visit: Patient navigator scheduled a primary care visit for the patient and offered to accompany them for up to three visits. If the patient had a PCP, the navigator aimed to schedule the appointment with that provider. If the patient did not have a PCP, the navigator scheduled the appointment with a local community-based clinic or hospital-based primary care center, depending on appointment availability and patient preference.<br><br>2. Help Preparing for PCP Visit: Immediately prior to the first PCP visit, the patient navigator met the patient at the providers office to review questions and concerns the patient wished to have answered by the PCP. During the PCP visit, the patient navigator reminded the patient of any questions or concerns that had not been addressed.<br><br>3. Help with action items after PCP visit: After the visit, the patient navigator and patient created a task list based on the providers recommendations. For example, if the PCP ordered additional tests or specialist referrals, the navigators assisted in scheduling these	Yes	Community advisory board or other community input	Justification not specific to individual components

Author, Year	Justification for intervention from publication[a]	Intervention description	Multiple Intervention Comp-onents	Was the intervention developed to respond to community or individual social need(s)?	Justification for specific components
		additional appointments, phoned patients to remind them, identified and addressed any barriers such as transportation, and encouraged patients to follow PCP recommendations.<br><br>4. As-needed help with other social needs: When needed, navigators helped patients to access medical transportation assistance through the state Medicaid system. If the patient identified social needs such as precarious housing, food insecurity, or insurance questions, they were provided with information to connect with local resources.<br><br>5. Regular Check-Ins: Finally, the patient navigators scheduled regular phone calls to each patient every 2 weeks during weeks 0 to 4 and every 4 weeks during weeks 13 to 52. During these calls, the patient navigator asked about the patient's health, healthcare appointments, and any social needs. Additionally, navigators phoned all patients after each medical appointment to check in, discuss follow-up information/instructions, and offer assistance with clinical and/or social needs.			

© 2024 Viswanathan M et al. *JAMA Network Open.*

Author, Year	Justification for intervention from publication[a]	Intervention description	Multiple Intervention Comp-onents	Was the intervention developed to respond to community or individual social need(s)?	Justification for specific components
Kempainen, 2023[36]	There are limited studies designed to assess the impact of providing food bank staples to adults experiencing food insecurity with type 2 diabetes (Flint et al 2020). In a nonrandomized, prospective pilot study using pre-post analysis, HbA1c improved after a 6-month intervention that included food bank access, primary care referral, and diabetes self-management support (Seligman et al 2015). A subsequent randomized trial showed improvements in food security and fruit and vegetable intake in people with diabetes receiving twice-monthly food from food banks, but no differences in glycemic control (Seligman et al 2018). Furthermore, recent research suggests weekly home-delivered meals may be associated with reduced medical spending (Berkowitz et al 2018). The goals of this study included assessing the feasibility and impact of home food delivery to adults with type 2 diabetes mellitus. This intervention adds to the current literature because of the intentional collaboration between Hennepin Healthcare and Second Harvest Heartland food bank to deliver culturally tailored food to patients utilizing a randomized, controlled study design.	1. Monetary compensation for participation;<br><br>2. Referral to food resources including SNAP;<br><br>3. Home-delivered food box tailored to nutritional needs and ethnic food preferences every 2 weeks to 24 weeks (30 to 33 pounds of food including shelf stable foods, recipes, and diabetes education materials in the language corresponding to box type)	Yes	No justification or explanation	Justification not specific to individual components
Kim, 2013[37]	Studies have shown that care management can improve health outcomes for patients with particular chronic health conditions, such as diabetes (Sidorov et al 2002; Dorr et al 2005; Glazier et al. 2006; Chin et al 2007), cardiovascular disease (Harris et al 2003; Sequist et al 2006), and congestive heart failure (Gorski and Johnson 2003; DeWalt et al 2006). For patients with depression, care management has encouraged patients to talk to mental health specialists, increased their use of antidepressants, reduced their depression, improved their health, and improved their work performance and job retention (Wang et al 2007; Mohr et al 2008; Kroenke et al 2010). In addition, care management has been found to reduce the costs of care in some settings (Handley,	1. Care managers assessed individual's healthcare and social service needs.<br><br>2. Care managers conducted telephone-based outreach; Care managers followed up annually with the individuals assessed as having few healthcare needs and to be compliant with their treatment plans; followed up every 3 months with the participants that had moderate to high medical costs and had new or serious diagnoses such	Yes	No justification or explanation	Justification not specific to individual components

© 2024 Viswanathan M et al. *JAMA Network Open.*

Author, Year	Justification for intervention from publication[a]	Intervention description	Multiple Intervention Comp-onents	Was the intervention developed to respond to community or individual social need(s)?	Justification for specific components
	Shumway, and Schillinger 2008), although this might not be the norm (Peikes et al 2009). Despite the success of care management in some settings, there has not been a rigorous evaluation of this approach in a diverse set of Medicaid beneficiaries with multiple chronic conditions. For example, studies of Medicaid care management in Florida (Afifi et al 2007; Kominski et al 2008) and Virginia (Zhang et al 2008) have found improvements such as fewer hospital stays and ED visits. However, those studies used nonrandomly chosen comparison groups, and the results may have confounded the effects of the programs with other unobserved differences between the groups (Rosenbaum and Rubin 1983; Bell et al 1995; Michalopoulos, Bloom, and Hill 2004). This is an important gap in the research as more than 20 states operate some form of care management for this population (Rosenman et al 2006; Arora et al 2008). This article begins to fill this gap in knowledge by presenting results from a randomized study of a telephone care management program for blind and/or disabled Medicaid beneficiaries in Colorado, many of whom often have multiple chronic conditions	as diabetes, congenital health failure, or chronic obstructive pulmonary disease; contacted participants assessed as high medical expenses but already well connected to a treatment system every 6 months<br><br>3. Care managers made sure that everyone had a primary care provider and helped to coordinate care across providers.<br><br>4. Care managers developed care plans with goals related to healthcare (such as reducing ED use) and social service needs (such as arranging for transportation to a doctor or helping the individual to find stable housing).<br><br>5. Care managers made referrals to behavioral or mental health services for individuals with psychiatric and/or substance use disorders.			
Kneipp, 2011[38]	TANF: Taken together, these findings suggested that a work-first TANF approach that does not take into account the health needs of this population is unlikely to yield long-term gains in either self-sufficiency or health-related outcomes….<br>Case mgt: Although we focused on ways to improve women's health through TANF-related system (and policy) enhancements, our research also builds on the literature documenting the effectiveness of public health nursing (PHN) case management with similar	1. Health screening questionnaire administered to reflect the Stages of Change approach.<br><br>2. Individualized case management (usually at least 4 60-minute meetings) in the Welfare Transition Program office or home. Case	Yes	Community advisory board or other community input	Justification not specific to individual components

© 2024 Viswanathan M et al. *JAMA Network Open.*

Author, Year	Justification for intervention from publication[a]	Intervention description	Multiple Intervention Comp-onents	Was the intervention developed to respond to community or individual social need(s)?	Justification for specific components
	populations of low-income, at-risk single mothers,33,34 the importance of tailoring intervention materials to specific groups,35,36 the difficulties low-income patients have in understanding Medicaid benefits, and the application of patient-centered interventions for improving health outcomes.36,37 Specifically, we report the outcomes from a randomized controlled trial that had the following aim: to test the efficacy of a PHN case management and Medicaid knowledge and skills training program for women enrolled in WTPs	management emphasized healthcare access or entry into primary care for newly identified symptoms; care coordination; health education; health and social service referrals; obtaining preventive services, screening, and routine care; and assistance in meeting health goals.<br><br>3. Training in navigating Medicaid services.			
Korr, 1996[39]	Case management: Experience with small demonstration projects for the homeless mentally ill funded during the mid-1980s suggested that discrete services (eg, case management without access to housing) did not work and that a comprehensive approach, with "services of greater intensity, diversity, and flexibility" would be more effective (Levine and Rog 1990, p. 966). The most frequently recommended service model is intensive case management (Cohen 1989; Susser, Goldfiger, and White 1990). These services need to be carefully evaluated using experimental designs. Lack of use of experimental designs has been cited as a limitation of other studies of case management with the mentally ill (Rubii, 1992) and with the homeless mentally ill (eg, Rife, Fist, Greenley et al 1991).<br>Bridege model: The major goal was to test whether the Thresholds "Bridge" model could be adapted for the homeless mentally ill. The Bridge model, based on the Madison, Wisconsin, Rogram of Assertive Community Training (Stein and Test 1980). provides assertive outreach and service coordination....Evaluations have shown that Bridge services reduce hospital use (Witheridge. Dincin, and Appleby 1982), reduce treatment costs (Bond 1984)	1. Staff bring the client from the hospital to the place where she/he will live in the community.<br><br>2. Staff link the client to entitlements such as Supplementary Security Income and to mental health treatment services, especially medication. Clients are also linked to rehabilitative services including supported employment.<br><br>3. Staff work at the client's home or in the community to teach living skills. During the first few weeks outreach includes almost daily visits.<br><br>4. Agency generally received the client's disability check and ensured that rent was paid.	Yes	No justification or explanation	Justification not specific to individual components

© 2024 Viswanathan M et al. *JAMA Network Open.*

Author, Year	Justification for intervention from publication[a]	Intervention description	Multiple Intervention Comp-onents	Was the intervention developed to respond to community or individual social need(s)?	Justification for specific components
	and in combination with crisis housing can avoid hospitalization (Bond et al 1989).				
Krieger, 1999[40]	One strategy to improve hypertension awareness and control is through community-based blood pressure measurement pro2,3 grams. Up to one third of persons whose blood pressures are assessed in community blood pressure measurement programs have elevated levels,4' and about one third of these are unaware that they have high blood pressure. "-0~6 In addition, community blood pressure measurement programs identify persons with treated hypertension who H have poorly controlled blood pressures.4<br>...We are aware of only one previous randomized trial demonstrating the effectiveness of an intervention to enhance follow-up, and this took place in an emergency department rather than a community setting.2 We therefore conducted a randomized controlled trial to determine whether a tracking and outreach intervention delivered by community health workers improved medical follow-up of persons whose elevated blood pressure was detected during blood pressure measurement at community sites.	CHWs provided:<br><br>1. referral to medical care and assistance in locating a provider<br><br>2. appointment scheduling assistance with reminders and follow-up<br><br>3. referrals to community transportation, childcare, or other services.	Yes	No justification or explanation	Justification not specific to individual components
Krieger, 2009[41]	home visit: Home visits are an alternative means for providing self-management support. Recently, several randomized trials demonstrated that home visits improve asthma control.25-32 Most of these studies focused on reducing exposure to household asthma triggers but did not address the medical aspects of self-management, such as effective use of medications and asthma action plans. Evaluation of comprehensive home-visit programs that include both environmental and medical self-management components is needed<br>CHW: Community health workers (CHWs) are particularly well suited for making visits to low-income, ethnically diverse households that are most affected	The CHWs shared ethnic backgrounds with participants and had personal or family experience with asthma. Their clients received 1 intake and an average of 4.5 follow-up visits during the course of a year as well as interim telephone communication. At the intake visit, CHWs reviewed participants asthma control, self-management practices, and access to medical care.	Yes	Community advisory board or other community input	Justification not specific to individual components

© 2024 Viswanathan M et al. *JAMA Network Open.*

Author, Year	Justification for intervention from publication[a]	Intervention description	Multiple Intervention Comp-onents	Was the intervention developed to respond to community or individual social need(s)?	Justification for specific components
	by asthma health disparities.33-40 However, evidence for the effectiveness of CHWs' provision of self-management support is needed.	Based on this assessment, results from a home environmental checklist and allergy testing, and use of motivational interviewing methods, CHWs developed a set of protocol-driven client and CHW actions.<br><br>At follow-up visits, CHWs assessed progress and reviewed a core set of educational topics (medication use, action plans, effective use of the medical system, medical adherence, and trigger reduction). Community health workers also provided social support and advocacy for clients (eg, housing issues, insurance coverage).<br><br>CHWs fit allergen-impermeable bedding encasements on the children's beds and gave participants a low-emission vacuum with a power head and embedded dirt finder, 2-layer microfiltration vacuum bags, a high-quality doormat, a cleaning kit, and plastic medication boxes.			
Krieger, 2015[42]	Asthma self-management is a cornerstone of asthma control,2,4-6 yet many people with asthma have not had the opportunity to learn self-management skills or do not practice them…. The effectiveness of home-based self-management support for improving asthma control among children is well-established,9,10 but	The CHWs provided:<br>1. Home visits<br>2. As-needed support via telephone, email, or additional home visits<br>3. Tailored asthma	Yes	No justification or explanation	Justification not specific to individual components

© 2024 Viswanathan M et al. *JAMA Network Open.*

Author, Year	Justification for intervention from publication[a]	Intervention description	Multiple Intervention Comp-onents	Was the intervention developed to respond to community or individual social need(s)?	Justification for specific components
	the effectiveness of home visits for adults has not been well studied.	management plan<br>4. Asthma self-management skills and education<br>5. Social support and advocated for client needs (e.g., housing issues, insurance)<br>6. Referrals to community resources<br>7. In-home asthma trigger reduction<br>8. Integrated pest-management<br>9. Coordination with participants medical homes including faxed summary of each visit to the clinic			
Lin, 2017[43]	Programs to reduce ED utilization among frequent ED users have employed intensive case management, care coordination and navigation, information sharing, disease management, and education.9-18 Community health worker (CHW) programs differ from traditional case management programs by employing community members, instead of licensed case managers or social workers, to assist with patient navigation. CHWs employed in ambulatory care settings have been shown to reduce ED visits and healthcare utilization among patients with chronic illness and recent hospitalizations; however, there are no peer-reviewed randomized controlled studies of the effects of CHW programs on ED visits among frequent ED users.19-23 Prior study results showing an association between ED-based CHW programs and decreased ED use among frequent ED users have been observational in design, and a recent systemic review of ED visit reduction programs concluded that high-quality, peer-reviewed evaluations of such programs are lacking.24,25 Furthermore, a minority of existing studies on ED visit reduction programs have included information on program costs and cost	1. interdisciplinary development of acute care plans to guide ED care<br><br>2. the assignment of an ED-based CHW who assisted with care coordination and addressed social issues contributing to unmet health needs of frequent users. CHW assessed unmet social needs via telephone intake. CHWs connected with participants to address needs over phone or home visits. The CHW assisted with specific tasks tailored to each patient's needs.	Yes	No justification or explanation	Justification not specific to individual components

© 2024 Viswanathan M et al. *JAMA Network Open.*

Author, Year	Justification for intervention from publication[a]	Intervention description	Multiple Intervention Comp-onents	Was the intervention developed to respond to community or individual social need(s)?	Justification for specific components
	savings.25 We conducted a randomized controlled trial of a pilot ED-based care coordination and CHW program in order<br>to reduce ED visits, hospitalizations, and associated costs among frequent ED users.				
Lipton, 1988[44]	Studies of chronic patients who are not homeless have demonstrated that residential programs can increase patients' stay in the community as well as improve their quality of life compared with programs offering standard forms of care, such as long-term hospitalization and traditional community-based aftercare (1 2-1 5). However, little attention has been given to the longitudinal interaction between homelessness, mental illness, and the efficacy of treatment interventions, and we are not aware of any studies examining the effectiveness of residential treatment for the homeless mentally ill. This paper describes a one-year study, begun in 1983, that compared homeless patients who were assigned to a residential treatment program after discharge from inpatient care with homeless patients for whom standard post-discharge planning and treatment were arranged. The study was initiated when 25 rooms became available at the opening of St. Francis Residence II, giving the authors a rare opportunity to conduct an experimental study of residential treatment for the homeless.	1. Furnished room<br><br>2. Individualized case management<br><br>3. Coordination of public assistance or Social Security benefits<br><br>4. Medication monitoring<br><br>5. Money management<br><br>6. Meals<br><br>7. Activity therapy<br><br>8. Referrals to psychosocial and rehabilitation programs when appropriate<br><br>9. On-site psychiatric treatment is provided and hospital admissions facilitated.	Yes	No justification or explanation	Justification not specific to individual components
Liss, 2019[45]	There is very limited evidence of transitional care interventions that reduce rehospitalization in vulnerable populations. Although a reengineered safety-net hospital discharge program reduced 30-day hospital encounters by 30%,8 other randomized trials with large proportions of low SES patients reported no reductions in 30-day readmissions.7, 9, 10 A patient navigator intervention in a public safety-net system	1. The initial appointment, scheduled within 10 days of index visit discharge, consisted of comprehensive psychosocial and medical assessments to identify barriers to care and systematically address modifiable barriers. At the	Yes	No justification or explanation	Justification not specific to individual components

© 2024 Viswanathan M et al. *JAMA Network Open.*

Author, Year	Justification for intervention from publication[a]	Intervention description	Multiple Intervention Comp-onents	Was the intervention developed to respond to community or individual social need(s)?	Justification for specific components
	had differential effects across age groups—19% reduction in hospital encounters among patients age ≥ 60, but 32% increase among patients age < 60—within 180 days of inpatient discharge.11Several transitional care interventions have reduced 30-dayinpatient readmissions,8, 12, 13 and a meta-analysis found greater effects in interventions with many components, involving more individuals in care delivery, or supporting patient capacity for self-care.14 However, the strength of evidence is low because of heterogeneity in interventions, patient populations, clinical settings, and implementation strategies.15 Additionally, 30-day follow-up has limited utility as an indicator of hospital quality,16, 17 and the costs associated with an initial inpatient stay may extend beyond 30 days,18, 19 suggesting a need for evaluating longer-term effects of transitional care interventions. In this context, we conducted a pragmatic comparative effectiveness trial of a transitional care practice that serves high-risk adults following discharge from a tertiary care academic medical center.... Multiple factors could have contributed to the TC intervention's effects on inpatient admissions. In accordance with prior meta-analysis findings,14 the TC intervention had multiple components, several individuals involved in care delivery, and attempted to improve patients' capacity for self-care.	beginning of the initial TC appointment, a social work assessment screened for insurance status, housing instability, food insecurity, ability to pay for medications and medical expenses, transportation barriers, social supports, mental illness history, and education level.<br><br>2. Following assessment, identified needs were addressed using patient-centered, individually tailored care plans. Patients who screened positive for depression or anxiety, reported a history of trauma, or requested substance abuse or behavioral health services were offered therapy, psychiatry, or connection to community substance abuse treatment. Follow-up appointments at the TC practice were scheduled as needed, with most patients initially seen weekly for medication management, assistance with insurance applications, and self-management support.<br><br>3. When the patient and care team determined the patient was ready to receive care from a community-based primary care provider, a health advocate			

© 2024 Viswanathan M et al. *JAMA Network Open.*

Author, Year	Justification for intervention from publication[a]	Intervention description	Multiple Intervention Comp-onents	Was the intervention developed to respond to community or individual social need(s)?	Justification for specific components
		scheduled an appointment within 14 days with a provider (typically at an FQHC) who would become the patient's new source of primary care. The TC care team deemed patients ready to launch from the practice after they met criteria including patient education through teach-back for multiple knowledge domains such as addressing transportation barriers for community-based appointments, and documentation required to receive financial discounts at scheduled primary care appointments and implementation of a sustainable medication regimen. At the time of launch, patients were also offered ongoing access to specialty care within the Northwestern Medicine system.			
Lopez, 2023[46]	Screening: Screening for social needs within the hospital is likely achievable. The iHELP study revealed that a brief screening intervention could be implemented into the pediatric hospital admissions process and increase the detection of social needs.6<br><br>Ameliorating SDOH: As postulated in the OASIS (Outcomes from Addressing SDOH in Systems) framework, ameliorating identified unmet needs has great potential to improve health and healthcare utilization outcomes.2Within the pediatric healthcare system, primary care physician (PCP) settings have led social needs screening and referral efforts. Work by Garg et al established that a process for	WE CARE Houston screened for alcohol, caregiver education, caregiver depression, childcare, drugs, employment, food security, health insurance, housing, immigration, IPV, legal assistance, learning English, literacy, parenting, paying for medications, PCP for a family member, tobacco, transportation, and utilities. WE CARE Houston community resource handouts (One-Pagers) were developed for	No	Other, Prevalence of social needs among similar target populations, in this case families of young children with inpatient hospital stays.<br><br>Literature review to determine social	NA, single intervention

Author, Year	Justification for intervention from publication[a]	Intervention description	Multiple Intervention Comp-onents	Was the intervention developed to respond to community or individual social need(s)?	Justification for specific components
	systematic screening and referrals during well-childcare increased families' receipt of community resources.10 Similarly, a systematic review by Gottlieb et al described several randomized controlled trials (RCTs) conducted in pediatric PCP settings that successfully performed social needs screening and community resource linkages.11	each of the needs screened. They contained the resource program name, a brief description of the program, and resource contact information. They were provided by the research assistant to the intervention group based on the caregivers screening results and receptiveness to resources. Social work was also consulted for housing needs or if the family requested assistance with any other need or if a social work consult was determined necessary by the primary medical team or staff. For caregivers in the intervention group, a 2-week follow-up call was performed to confirm receipt of resource pages and to offer a new copy via email, if needed.		needs for which to screen	
Lumba-Brown,2020[82]	The novel "Empowering Youth Through Interpersonal Violence Prevention Program" (EYIPP) was implemented and developed through Social Work in the Pediatric ED at St. Louis Children's Hospital (SLCH) in 2012 as a prospective pilot study for feasibility in reducing recidivism, morbidity, and mortality in youth violence victims. The program's mission was to support a childhood free of interpersonal violence by establishing a mentoring intervention to reduce recidivism, morbidity, and mortality in youths presenting to the St. Louis Children's Hospital ED following interpersonal violence-related injury. Youth mentoring is effective when best practices are utilized and when strong relationships are formed between mentors and	EYIPP mentors are Master's level social workers, with further training for the program focusing on the five core competencies for healthy adjustment in adolescence.<br><br>Mentors acted as advocates, liaisons, therapists, and as a channel for resources for youths in the EYIPP program. They were available to youths in scheduled meetings as well as on-call via phone, described further in the program	Yes	Community advisory board or other community input, Prevalence of social need(s) in the community	Justification not specific to individual components

© 2024 Viswanathan M et al. *JAMA Network Open.*

Author, Year	Justification for intervention from publication[a]	Intervention description	Multiple Intervention Comp-onents	Was the intervention developed to respond to community or individual social need(s)?	Justification for specific components
	youths (Rhodes, 2008; Sipe 2002). In addition, youths from backgrounds of environmental risk may benefit from participation in mentoring programs (DuBois, Holloway, Valentine, and Cooper 2002). Co-location of social work and medical services in the pediatric ED allows immediate engagement of every victim of assault. Two recent studies published in Pediatrics demonstrated that high-risk youth presenting to EDs have increased risk for subsequent firearm violence and that 23% of these youths have been in recent possession of a firearm (Carter 2013; Carter 2015). These youths represent the target audience for violence intervention programs which naturally begin in the ED. Evidence demonstrates that co-location of pediatric behavior health services and medical care increases patient utilization of behavioral health services by 20% and overall pediatric behavioral health ED visits by 245% (Hacker et al 2015). Hospital-based violence intervention programs in adolescents and adults have previously demonstrated reductions in recidivism and overall cost-effectiveness (Gomez et al 2012; Juillard et al 2015; Purtle, Rich, Bloom, Rich, and Corbin 2015; Scott, Tepas, Frykberg, Taylor, and Plotkin 2002; Smith, Dobbins, Evans, Balhotra, and Dicker 2013).	description. They attended court hearings, met with deputy juvenile officers, school personnel, agencies that are already serving the family, police, and medical staff to advocate for the family and youth. Mentors also worked with willing caregivers to provide mediation and therapeutic counseling as well as modeling of parenting skills. The mentor's overall goal was to empower the youth and family to make decisions that will lead to healthy life choices and reduce recidivism:<br><br>1. Mentors met with the youth and their caregiver in the home, or inpatient if the patient was hospitalized, to discuss the services to be provided (advocacy, therapeutic interventions, education and development of conflict resolution and anger management skills) and identify goals that the family had for the program as well as mutually agreed upon places for future meetings.<br>2. Mentors met with youth program participants individually in the community as well, establishing a treatment plan with mutually agreed upon goals. A minimum of six			

© 2024 Viswanathan M et al. *JAMA Network Open.*

Author, Year	Justification for intervention from publication[a]	Intervention description	Multiple Intervention Comp-onents	Was the intervention developed to respond to community or individual social need(s)?	Justification for specific components
		sessions with the mentor was required over the follow-up period of 1 year; otherwise there were no limits to the time frame of the services or set intervals for mentor–youth meetings; these were determined by the youth's needs. The services and intervals were adjusted as the youth progressed in the program			
MacKinney, 2013[47]	One delivery model, called Project Access (PA), provides individuals who are not eligible for Medicare or Medicaid, and are financially unable to buy health insurance, access to free high-quality primary and preventive care from volunteer clinicians. PA model programs typically support the volunteer clinicians by providing free basic laboratory and radiology services. A variety of approaches are used to provide participating patients with the drugs needed to manage their chronic conditions at little or no cost. In general, PA does not provide ER or hospital care, but seeks to minimize the frequency with which participants use such services. The PA model has been successfully implemented in dozens of United States communities, ranging in population from less than 20 000 to more than 2 000 000. However, although one rationale for the program is that it will reduce costly ER visits and hospital stays, no methodologically rigorous study has sought to establish such an effect. We therefore sought to determine whether a PA model program, MilwaukeeCares, would reduce ER visits and hospitalizations among persons given immediate access to the program, compared with wait listed controls	MilwaukeeCares is an administrative system that (1) identifies uninsured individuals, (2) identifies healthcare providers willing to provide free services, and (3) connects the person in need to an appropriate primary healthcare provider.	Yes	Prevalence of social need(s) in the community	Justification not specific to individual components

© 2024 Viswanathan M et al. *JAMA Network Open.*

Author, Year	Justification for intervention from publication[a]	Intervention description	Multiple Intervention Comp-onents	Was the intervention developed to respond to community or individual social need(s)?	Justification for specific components
McClintock, 2017[48]	Our study is the first study to address both biomedical needs and social determinants of health in hypertension and depression care with a focus on blood pressure control in primary care settings.<br>The purpose of this study was to carry out a randomized controlled pilot trial to test the effectiveness of an integrated intervention for hypertension and depression incorporating patients' social determinants of health (enhanced intervention) versus an integrated intervention alone (basic intervention). Our intervention builds upon our prior work with a problem-solving based intervention (Bogner, Morales, de Vries, and Cappola 2012). It was hypothesized that in a sample of primary care patients prescribed pharmacotherapy for hypertension, patients who were randomized to receive the enhanced intervention compared with the basic intervention would demonstrate the following after a 12 week period: (1) lower blood pressure; and (2) fewer depressive symptoms. In addition, we postulated that the most common social determinants of health that patients with hypertension would want to address would be transportation, financial, or emotional needs	1. Patients identified their priority needs through a card sort<br><br>2. The interventionist created personalized management plans with patients based on the prioritized needs.	Yes	No justification or explanation	Justification not specific to individual components
Melnikow, 1997[49]	Prenatal care programs have been created to improve appointment compliance by providing case management services or various forms of outreach or incentives, including infant gifts, lotteries for prizes, and transportation, but few randomized controlled trials evaluated these interventions. We conducted a randomized controlled trial in a system of northern California family planning clinics providing prenatal care to low-income women to evaluate the effect of either a taxicab voucher for transportation to the clinic or an infant gift incentive on compliance with the first prenatal appointment.	A voucher that could be redeemed for a taxicab ride to and from the first prenatal clinic appointment	No	Prevalence of social need(s) in the community	NA, single intervention

© 2024 Viswanathan M et al. *JAMA Network Open.*

Author, Year	Justification for intervention from publication[a]	Intervention description	Multiple Intervention Comp-onents	Was the intervention developed to respond to community or individual social need(s)?	Justification for specific components
Mion, 2003[50]	A number of case management or care-coordinated programs have been implemented successfully with hospitalized elderly patients.20-26 One model not dependent on a specific disease category, the transitional model of care, has been shown to be effective for hospitalized older patients.27,28 The transitional model uses a comprehensive discharge planning protocol implemented by advanced practice nurses and short-term home follow-up by the advanced practice nurse in lieu of a visiting home nurse. Naylor et al27,28 devised the transitional model on the premise that timely and comprehensive assessment of older hospitalized patients of needed home services would reduce subsequent costly service use (ie, hospital readmissions and ED visits).They have demonstrated that the model reduced rates of hospital readmission, increased the time from the index hospital discharge to readmission, and lowered healthcare costs over the ensuing 24 weeks compared with those of older hospitalized patients receiving usual discharge and follow-up community services.28 The hospital transitional model of care might be applicable to older adults in the ED because many elderly ED patients have multiple comorbid illnesses and concomitant psychosocial needs. We theorized that identifying unmet medical, health, and social needs of older ED patients and linking them to appropriate follow-up medical care, social, or home healthcare services would delay functional decline and subsequent nursing home admission, reduce repeat ED visits, and lower hospital rates. Thus, we established a formalized linkage and referral system between an ED-based advanced practice nurse and 10 public and not-for-profit social and health community agencies.29	1. Screening for social needs and provision of care plan and referral by ED nurse.<br><br>2. ED recommendations for follow-up care or use of community agencies were given to participants or their proxies. Participants were responsible for contacting the suggested community agency or primary care provider whenever ED personnel recommended use of community services.<br><br>3. The advanced practice nurses assumed responsibility for discharge planning while the patient was in the ED, with short-term telephone follow-up as necessary until community agency personnel achieved contact with the participant.<br><br>4. Usual and customary ED care.	Yes	No justification or explanation	Justification not specific to individual components
Nyamathi, 2001[51]	Researchers experienced in providing successful interventions for dysfunctional women contend that one of the most empowering techniques is the use of	1. Women and their intimate partners engaged in a 2-hour session weekly for 6 weeks	Yes	Prevalence of social need(s) in the community	Justification not specific to

© 2024 Viswanathan M et al. *JAMA Network Open.*

Author, Year	Justification for intervention from publication[a]	Intervention description	Multiple Intervention Comp-onents	Was the intervention developed to respond to community or individual social need(s)?	Justification for specific components
	peer mentors, individuals who are respected and recognized as natural helpers, educators, and role models (Dearing, Larson, Randall, and Pope 1998).At present, however, little is known about the ability of peer mentors, trained and supported by nurses, to provide effective AIDS education and prevention programs to homeless persons. Moreover, no studies to date have been reported in the literature on whether programs can be delivered successfully to intimate homeless couples, and whether programs can be effective in promoting behavior change in both partners despite gender differences.... Broadhead et al (1998) argue that peers are most effective in recruiting and educating drug users because the latter are more likely to listen to people whom they consider to be like themselves, and as trusted individuals, peers may carry more weight than healthcare professionals. Moreover, natural helpers are thought to be<br>effective in promoting behavioral change by improving coping skills in dealing with difficult situations where unsafe behaviors commonly are used (Nutbeam, Blakey, and Pates 1991). On the other hand, the nurse case-managed approach has been successfully applied in intervention studies aimed at-risk reduction (Nyamathi, Stein, and Brecht 1995). Its success appears to lie in the comprehensive approach used. Yet no investigators have examined whether nurses can be more effective than either peer mentors or nurses and trained counselors who provide standard community AIDS testing and counseling in reducing HIV-related drug and sexual behaviors or in improving the cognitive and psychological resources of homeless persons.... Researchers have long recognized that a supportive person can provide encouragement to practice health-promoting behaviors (Norman, Talbott, Kuller, Krampe, and Stolley 1991). However, significant others also can	conducted by a peer mentor and outreach worker of the same ethnicity as the patients. These mentors had led lifestyles similar to the clients but were now sober with stable housing. This program provided group education (with one or two other couples) information on HIV/AIDS, risk behaviors, and risk-reducing and health-protecting behaviors.<br><br>2. Entry into needed agencies such as outpatients services, clinics, and social services were facilitated.<br><br>3. Culturally and linguistically appropriate materials were distributed.<br><br>4. Individuals were provided ongoing assistance in obtaining needed healthcare services.			individual components

© 2024 Viswanathan M et al. *JAMA Network Open.*

Author, Year	Justification for intervention from publication[a]	Intervention description	Multiple Intervention Comp-onents	Was the intervention developed to respond to community or individual social need(s)?	Justification for specific components
	have a negative impact on the practice of health-promoting behaviors. For example, significant others may encourage drug use and provide barriers to entering drug treatment programs (Latkin et al1996; Neaigus et al 1994). As a result, there has been mounting interest in including the intimate partners of homeless women in AIDS education and prevention programs in order to increase the effectiveness of those programs.				
O'Brien, 1999[52]	A few studies have investigated use of interventions to connect ED patients with primary care providers, including extensive patient education by nurses in the ED, referral from ED triage areas directly to office sites, and referral from ED triage to lists of available sites.8-11 The latter 2 interventions involve turning the patient away from the ED without being seen by a physician. An earlier study at Rhode Island Hospital found that 25% of ED patients do not have a primary care physician, and 25% report using the ED as their regular source of care.8 In an attempt to improve the primary care of this population, we tested a novel intervention designed to refer regular ED users to primary care.	1. Appointment for a mutually agreeable reserved time in the Medical Primary Care Unit within 2 to 3 weeks<br><br>2. Information on the date and time of the appointment, the physician's name, written directions and a map to the Medical Primary Care Unit, and information about services provided in the clinic, the clinic policy of caring for patients regardless of their ability to pay or insurance status, and availability of free medications through various programs	Yes	No justification or explanation	Justification not specific to individual components
O'Connell, 2018[53]	Rigorous research over the past 25 years, including an experimental cost-effectiveness study of supported housing, has demonstrated that programs like HUD-VASH and Housing First that offer direct access to rental subsidies, without preconditions, effectively contribute to reductions in homelessness and successful transitions into permanent and stable housing among homeless individuals living with mental illness and substance use disorders (Aubry,Goering, et al 2016; Brown, Jason, Malone, Srebnik, and Sylla 2016; Goldfinger et al 1999;	1. Veterans met with a case manager who assisted them in obtaining a Section 8 rent subsidy and locating an apartment.<br><br>2. Case manager provided intensive case management support without a specified time limit.	Yes	No justification or explanation	Justification not specific to individual components

© 2024 Viswanathan M et al. *JAMA Network Open.*

Author, Year	Justification for intervention from publication[a]	Intervention description	Multiple Intervention Comp-onents	Was the intervention developed to respond to community or individual social need(s)?	Justification for specific components
	Hurlburt, Wood, & Hough, 1996; Kasprow, Rosenheck, Frisman, and DiLella, 2000; Lehman, Dixon, Kernan, DeForge, and Postrado 1997; Lipton, Siegel, Hannigan, Samuels, and Baker 2000; Rosenheck, Kasprow, Frisman, and Liu-Mares 2003; Shern et al 1997, 2000; Tsemberis, Moran, Shinn, Asmussen, and Shern 2003). Research and expert consensus agree that the critical ingredient for successful housing outcomes in such programs seems to be direct access to a housing rental subsidy (Aubry, Nelson, & Tsemberis 2015; Aubry, Goering, et al 2016; Nelson and MacLeod 2017; Rosenheck, 2010; Stergiopoulos et al 2015; Tsemberis 2010). Yet, in Housing First models, successful housing is but an initial, albeit essential, step or means to achieving broader personal goals and improvements in quality of life (Aubry, Nelson, et al 2015). The underlying premise of Housing First is that direct access to rental subsidies and supports are thought to provide an ontologically secure foundation (i.e., feelings of safety and security in the stability of one's home [Padgett, 2007]) from which participants can make improvements in other areas of life, such as improved relationships with others, greater engagement in treatment, reductions in inpatient and emergency department use, reductions in criminal activity, and increased access to income or benefits (Macnaughton et al 2016). According to the model, these improvements can, in turn, lead to enhanced community integration, social support, and overall quality of life (Aubry, Nelson, et al 2015).Data from some, but not all, observational research studies support the notion that direct access to rental subsidies may contribute to broader, more positive gains in clinical, psychosocial, and functional outcomes. In supported housing research, the degree to which a person experiences an emotional connection with and membership in one's community				

© 2024 Viswanathan M et al. *JAMA Network Open.*

Author, Year	Justification for intervention from publication[a]	Intervention description	Multiple Intervention Comp-onents	Was the intervention developed to respond to community or individual social need(s)?	Justification for specific components
	(ie, psychological integration [Wong, 2002]) has been associated with greater social support and cohesion, quality of housing, more positive interactions with neighbors, sense of belonging, and ontological security (Cherner, Aubry, and Ecker 2017; Ecker and Aubry 2016, 2017; Nemiroff, Aubry, and Klodawsky 2011; Padgett 2007; Prince and Prince 2002; Yanos, Felton, Tsemberis, and Frye 2007). Other research suggests that having interpersonal and community resources, and especially, having a larger social support network, access to subsidized housing, and greater income were associated with greater residential stability (Aubry, Duhoux, Klodawsky, Ecker, and Hay 2016). Support from family and friends (Caton et al 2005; O'Connell and Rosenheck 2016; Zlotnick, Tam, and Robertson 2003) and support from service providers (Patterson, Currie, Rezansoff, and Somers 2015) appear to be important resources that contribute to leaving homelessness. These theories provide potential explanations as to how access to housing may enhance quality of life, but to our knowledge, no research has sought to empirically identify mediators of the relationship between housing subsidies, the key ingredient of experimental demonstrations of effective supported housing programs, and quality of life.				
O'Toole, 2015[54]	The health event itself can be a treatable moment for effecting behavior change among homeless patients, where there is often a situational motivation that can facilitate behavior changes.11 Perceiving a need for care—either from a recent diagnosis or from symptoms—is consistently associated with health seeking behavior.13	1. Personal Health Assessment: Research nurse interviewed participants about their medical history, health behaviors, and housing situations provided feedback and a brief intervention using motivational interviewing and presented summary of findings and their implications to the participant<br><br>2. Clinic Orientation: Following	Yes	Prevalence of social need(s) in the community	Justification not specific to individual components

Author, Year	Justification for intervention from publication[a]	Intervention description	Multiple Intervention Comp-onents	Was the intervention developed to respond to community or individual social need(s)?	Justification for specific components
		assignment and receipt of either Personal Health Assessment or usual care, participants were transported to the clinic by the research assistant, introduced to the clinic team (either the patient-aligned care team or homeless patient-aligned care team), depending on patient preference and team availability), given clinic tour and additional resources available at the clinic (clothes, hygiene kits, food, and benefits representatives, available to all homeless Veterans regardless of primary care enrollment)			
Post, 2021[55]	Case management has the potential to address the needs of complex patients, reducing service use and improving quality10, 11...While a number of studies have reported success in reducing ED use through case management,11, 15–18 the evidence suffers from methodological flaws, including uncontrolled pre-post studies susceptible to mean reversion12. Randomized studies are extremely rare in community-based case management interventions19 Part of a federally funded State Innovation Model20 program, the intervention sought to bridge the siloed provision of medical, mental health, and social services to address the complex and cross-sector needs of patients	Participants in the intervention were assigned to community-based case management agencies, where case workers met with participants to conduct an inventory of their social and medical needs. The case worker then contacted community resources to assist participants in navigating health and community systems.	No	No justification or explanation	NA, single intervention
Raven, 2020[56]	Permanent supportive housing , defined as subsidized housing with closely linked, voluntary supportive services (eg, case management, physical and mental health services, substance use treatment services) provides permanent housing for people with chronic homelessness and behavioral health conditions.10	1. Permanent supportive housing covered by rental subsidy<br><br>2. linked or on-site supportive services including case	Yes	No justification or explanation	Justification not specific to individual components

© 2024 Viswanathan M et al. *JAMA Network Open.*

Author, Year	Justification for intervention from publication[a]	Intervention description	Multiple Intervention Comp-onents	Was the intervention developed to respond to community or individual social need(s)?	Justification for specific components
	Permanent supportive housing is offered on a "housing first"11 basis, meaning clients are not required to be sober or engage in treatment.	management, physical and mental healthcare, substance use treatment services and vocational support.			
Sadowski, 2009[57]	Missing are intervention studies of homeless individuals with any chronic medical illness. Our study sought to determine whether an intervention that provided housing and case management for homeless adults with chronic medical illness would reduce hospitalizations and visits to the emergency department.	1. Provision of transitional housing at respite care centers - hospital case managers facilitated discharge planning and placement in respite care.<br><br>2. Placement in stable housing facilitated by respite and housing case managers.<br><br>3. Case management - intervention case managers coordinated medical care, substance abuse and mental health treatment referrals as needed.	Yes	Community advisory board or other community input	Justification not specific to individual components
Schickedanz, 2023[58]	The American Academy of Pediatrics (AAP) recommends all families be screened for financial hardship as part of preventive healthcare visits,7,8 and medical and professional organizations have recommended that clinical care address upstream economic determinants of health.9–14 A growing literature has shown clinic-based free tax preparation is readily accepted and delivers substantial income benefits.16,19,20 Clinically integrated financial services could lead to improved health outcomes through reduced financial stress, greater healthcare visit adherence, and higher preventive healthcare service receipt. Financial coaching has grown over the past decade as a standardized, evidence-based, and strengths-based approach to helping low-income families achieve their economic potential through client driven pursuit of financial goals and antipoverty	1. Intervention included usual care (telephonic clinic visit reminder calls from clinic staff 1 to 2 days in advance with voicemails left and 3 call attempts if calls were unanswered), a text message visit reminder from the health system roughly 3 days before the appointment, a mailed postcard visit reminder, and paper-based social needs screenings at each preventive visit (the same reminders and social needs screenings received by the intervention group); pediatricians and nurse	Yes	No justification or explanation	Yes

Author, Year	Justification for intervention from publication[a]	Intervention description	Multiple Intervention Comp-onents	Was the intervention developed to respond to community or individual social need(s)?	Justification for specific components
	services to improve financial literacy, income, budgeting, savings, and credit.22 In addition to regular 1-to-1 visits with a coach in which participant financial capability goals are identified and addressed through motivational interviewing and planning, financial coaching programs also routinely connect clients with underused financial resources, including federal antipoverty programs. Financial coaching has been shown to reduce financial stress, increase income, build savings, reduce debt, and improve credit in experimental and quasi-experimental studies.23–25	practitioners provided external resource referrals in response to social needs screening. Timing of follow-up not specific to component<br><br>2. The intervention included financial coaching. Financial coaches and parents worked on furthering parent financial goal plans to address areas of greatest financial potential jointly determined by participant and coach. Goals and action plans were developed through motivational interviewing and shared decision-making with coaches at each visit. Coaching also included assessment of income (including public benefits eligibility/receipt), savings, credit, debt, and taxes, as well as expense reduction opportunities through household budgets. Coaches identified parent strengths and navigated participants to and through cost-saving services and public benefits, such as affordable childcare, transportation and utilities discounts, nutrition assistance, and free tax preparation to access child tax credits. Financial coaching encounters in clinic lasted approximately 20 to 40 minutes (often before/after primary pediatricians met with the			

© 2024 Viswanathan M et al. *JAMA Network Open.*

Author, Year	Justification for intervention from publication[a]	Intervention description	Multiple Intervention Comp-onents	Was the intervention developed to respond to community or individual social need(s)?	Justification for specific components
		families), and parents were provided written referral materials and diapers when requested. Timing of follow-up not specific to component<br><br>3. Parents communicated with their coaches between visits via phone and text-based messaging at least monthly to advance financial goals. Timing of follow-up not specific to component			
Schumacher, 2017[59]	Interventions to increase patient engagement increase the use of preventive care, reduce hospital-based care and improve outcomes.6,8-12... Coaching interventions increase PAM scores, reduce hospital use, and improve medication and chronic disease self-management but have not been tested in the ED.8,9,12 Although ED use is increasing in older adults, those with limited health literacy represent a particularly high-risk group who are often under-engaged in managing their health and frequently turn to the ED for care.14-18 Strategies aimed at engaging these patients at the critical ED juncture may help them stay engaged, better manage their health and avert future health crises. We tested the impact of a coaching intervention on patient engagement and follow-up doctor visits in chronically ill patients.	The ED-to-home intervention was modeled on the CTI, an evidence-based program to increase patient engagement and reduce 30-day readmissions and healthcare costs in hospitalized patients. Trained coaches from community area agencies on aging administered the intervention. Coaches helped patients 1) schedule follow-up doctor visits; 2) recognize disease worsening; 3) reconcile medications; and 4) communicate with providers. Coaches visited patients homes within three days of ED discharge, called three times over the ensuing month, and engaged patients by helping them set achievable goals.	Yes	No justification or explanation	Justification not specific to individual components
Sege, 2015[60]	Some elements of the solution have already been demonstrated: care coordination in the context of the	1.Family support specialist: in person meetings, home visits,	Yes	Other, based on the evidence-based	Justification not specific to

© 2024 Viswanathan M et al. *JAMA Network Open.*

Author, Year	Justification for intervention from publication[a]	Intervention description	Multiple Intervention Comp-onents	Was the intervention developed to respond to community or individual social need(s)?	Justification for specific components
	patient-centered medical homes improves healthcare delivery for children with and without special healthcare needs.28 This report describes the outcomes of a randomized trial of a new approach to improve care of newborns and their families, Project Developmental Understanding and Legal Collaboration for Everyone (DULCE). DULCE implemented the Strengthening Families approach26 in a pediatric primary care setting by developing a new program based on previous efforts: the evidence-based Healthy Steps30,31 and Medical-Legal Partnership (MLP)32–34 models.	and phone contacts<br><br>2.MLP consultations and referral to resources<br><br>3.Collaborative routine healthcare visits<br><br>Families could access the family support specialist by telephone, email, text, or personal visits at the clinic. Email, text, and community visits were initiated or requested by participant.		Healthy Steps and Medical-Legal Partnership (MLP) models	individual components
Shinn, 2015[61]	The Critical Time Intervention (CTI) employs time-limited case management to support severely mentally ill men and women at risk for recurrent homelessness. Randomized controlled trials show after 18 months that adults assigned to CTI spend less time homeless (Herman et al 2011; Susser et al 1997), report reductions in psychiatric symptoms (Herman et al. 2000), exhibit lower risk for psychiatric hospitalization (Tomita and Herman 2012), and save significant costs to the homelessness service system (Jones et al 2003). This study examines an adaptation of CTI targeting homeless families with mental health problems...Family Critical Time Intervention (FCTI) represents a community-based service model for families using homeless shelters (Felix and Samuels 2006).	1. The first phase, Transition to Community, begins when families arrive at the shelter. A case manager completes a thorough family assessment that includes caregiver and child strengths and challenges and then works intensely with the mother, meeting as often as three times per week, to link the family with community resources at local agencies.<br><br>2. The second phase, TryOut, tests and adjusts the support systems established while at the shelter during the family's move into the community. There is less contact, as families are encouraged to take more control over following through with services and programs on their own. The case manager observes where the mother and	Yes	No justification or explanation	Justification not specific to individual components

© 2024 Viswanathan M et al. *JAMA Network Open.*

Author, Year	Justification for intervention from publication[a]	Intervention description	Multiple Intervention Comp-onents	Was the intervention developed to respond to community or individual social need(s)?	Justification for specific components
		family need more or fewer supports and services. The case manager aims to develop trust with the mother while maintaining boundaries around service delivery and provides her with trial and error experiences of connecting with resources in a positive manner.<br><br>3. In the final phase, Transfer to Care, the case manager reduces contact further, as families are encouraged to take full responsibility for accessing services. The case manager works with the mother to review and bolster the family's support system to ensure long-term community-based linkages.			
Shumway, 2008[62]	A variety of interventions that differ in complexity and intensity have been evaluated in preliminary studies, with promising results. A randomized trial of 3 modest interventions that provided 16 primary care providers with information about their patients' ED and hospital admissions significantly decreased ED use because providers used the information to monitor and modify their patients' service needs [10]. In a pre-post study on 24 ED frequent users, a case management program designed to involve a range of community care providers in the development of comprehensive care plans markedly reduced ED use [11]. A more comprehensive case management program designed for ED frequent users with substance use disorders was evaluated in a study comparing 10 case-managed patients with 8 similar patients who did not receive case management [12]. Relative to the year before the study, a 58% decrease in ED use was	1. Psychosocial needs assessment<br><br>2. Long-term clinical case management that included crisis intervention and individual and group supportive therapy.<br><br>3. Long-term clinical case management also included assistance in obtaining stable housing and income entitlements, linkage to medical care providers, referral to substance abuse services when needed, and ongoing assertive community outreach to maintain continuity of care.	Yes	Prevalence of social need(s) in the community	Justification not specific to individual components

© 2024 Viswanathan M et al. *JAMA Network Open.*

Author, Year	Justification for intervention from publication[a]	Intervention description	Multiple Intervention Comp-onents	Was the intervention developed to respond to community or individual social need(s)?	Justification for specific components
	observed in the case management group in the year after study entry, whereas no change in use was observed in the comparison group. Preliminary evaluations of hospital-based comprehensive clinical case management programs designed for all ED frequent users have yielded varied but promising results. One study examined the impact of hospital-based integrated case management in a pre-post study on 60 ED frequent users, comparing ED use and psychosocial problems in the 12 months before and after initiation of case management [13]. Case management was associated with improvements in housing status and linkages with medical and community services but was also associated with increased ED use. A second study evaluated clinical case management in a pre-post study on 53 patients, comparing hospital use, hospital costs, and psychosocial problems in the 12 months before and after the case management intervention [14]				
Sood, 2021[63]	ActionHealthNYC was a 1-year "direct-access" demonstration program that provided comprehensive, patient-centered, and coordinated care through a primary care home (PCH) model without involvement of a health insurance plan.10 Existing literature on patient-centered medical homes (PCMHs) has yielded mixed results, with some patient-centered medical homes not being associated with reduced cost, improved quality, or increased access to primary care.11 The direct-access model leverages components of patient-centered medical homes likely to improve access and provides comprehensive health services through a limited network within a confined geographical area. Although these programs do not offer insurance, they include transparent pricing, care coordination, and linkages to PCHs.12 Our goal was to leverage the direct-access model to increase access to care for uninsured NYC residents who did not qualify for insurance such as Medicaid or	1. Primary Care Homes offered comprehensive primary care and specialty care referrals as needed<br><br>2. Primary care appointments, appointment reminders, and outreach for missed appointments<br><br>3. Written care plan<br><br>4. Enhanced care coordination for selected participants (at least 6 care coordination encounters, pre-visit planning, and referrals to social services)<br><br>5. Member handbook which	Yes	No justification or explanation	Justification not specific to individual components

© 2024 Viswanathan M et al. *JAMA Network Open.*

Author, Year	Justification for intervention from publication[a]	Intervention description	Multiple Intervention Comp-onents	Was the intervention developed to respond to community or individual social need(s)?	Justification for specific components
	qualified health plans through the state's health insurance marketplace.	described how to get care, associated costs, and how to access customer service<br><br>6. Customer service for questions, complaints, and changes to the Primary Care Homes			
Talavera, 2021[64]	Achieving adequate disease management is hampered in Latinos by a lack of access to quality, culturally appropriate care that accommodates the significant influence of social determinants of health, which play a significant role in perpetuating health disparities [6]. In addition, an emphasis on psychosocial well-being is necessary to effective self-management support in all populations with diabetes [6–8]. This may be especially important in Latinos with diabetes who are more vulnerable to experiencing depression and diabetes distress [9], which in turn predict worse diabetes outcomes [10]. To meet the medical, social, and psychological needs of Latinos with diabetes, a patient-centered team-based care approach that adheres to the chronic care model is needed [11]. Within this framework, the integration of medical and behavioral healthcare can address the comorbidity of chronic disease and behavioral health concerns [12]. Integrated care models are effective in improving health among patients with diabetes and depression in primary care settings [13] and reducing healthcare costs and hospitalizations [14, 15]. However higher levels of integrated care are challenging to accomplish within the setting of Federally Qualified Health Centers [16], which provide care to large numbers of Latinos with diabetes. The aim of the current study was to examine the effectiveness of "Latinos Understanding the Need for Adherence in Diabetes (LUNA-D)," a culturally appropriate, patient-centered, team-based, highly	1. Co-location of the clinical team<br><br>2. Warm hand-off from the medical provider to the behavioral health provider<br><br>3. Shared treatment plan<br><br>4. Integrated medical visits with the medical provider and the specialty behavioral health provider<br><br>5. Care coordination<br><br>6. Culturally appropriate, group-health education classes led by a community health worker	Yes	Other, In the discussion: "Our model design was guided by these consensus statements, application of community-based participatory-research principles, and lessons learned from previous efforts with our Federally Qualified Community Health Center partner."	Justification not specific to individual components

Author, Year	Justification for intervention from publication[a]	Intervention description	Multiple Intervention Comp-onents	Was the intervention developed to respond to community or individual social need(s)?	Justification for specific components
	integrated model of diabetes care, at improving diabetes clinical control, as defined by HbA1c, blood pressure, and lipid levels, in a Federally Qualified Community Health Center.				
Theeke, 2016[65]	At present, healthcare providers do not have access to a recommended clinically effective treatment for loneliness. Interventions for loneliness have ranged from overemphasis on social reintegration [20–24] to individual cognitive therapies, such as mindfulness [25]. No single intervention has been determined as effective for diminishing loneliness and its negative health outcomes across age groups or populations. One recent meta-analysis of interventions suggested that effectiveness may be enhanced if interventions targeted common thought process errors that occur with loneliness [26], such as automatic thinking [27] or fears and phobias [28]. In response to this body of knowledge, we developed LISTEN, a novel intervention for loneliness. The initial development, feasibility, and acceptability of LISTEN have been published [29,30]. This initial study on LISTEN was conducted in West Virginia, a state located entirely in Appalachia. The Appalachian region is a known area of health disparity [31]. A disproportionate segment of the region's population is rural [32], impoverished, lowly educated, and physically ill, all of which are linked to poor mental health outcomes [33]. Therefore, more adults in Appalachia may be suffering from loneliness [15] or untreated depression [34]. The rural nature of Appalachia coupled with the lack of public transportation may limit social contacts. In spite of the stereotypical view of Appalachians living rurally with close extended family, other forms of social support may be more important than family proximity in predicting mental health outcomes [35]. Particularly for women, emotional support from friends has been reported to enhance affect [36]. Knowing that adults living rurally may experience a chronic condition with	1. LISTEN is delivered sequentially and weekly, in five 2-hour sessions. The content of the sessions was derived from the health and social science literature on loneliness, and the sessions are designed to be sequential, focusing first on belonging, then relationships, role in community, loneliness as a health challenge, and meaning of loneliness. Participants begin each session with writing; during weeks 1 to 4, the participants complete unique homework assignments relevant to the content for the upcoming week.<br><br>2. Reminder letters and phone calls were used for the weekly group sessions.	Yes	Prevalence of social need(s) in the community	Yes

© 2024 Viswanathan M et al. *JAMA Network Open.*

Author, Year	Justification for intervention from publication[a]	Intervention description	Multiple Intervention Comp-onents	Was the intervention developed to respond to community or individual social need(s)?	Justification for specific components
	a sense of quiet pride [37] makes it even more important that healthcare providers proactively assess and address problems such as loneliness and depression. Findings from preliminary qualitative studies on lonely older women living in Appalachia encouraged the development of LISTEN [29]. Lonely older women suffering from chronic illness and living in Appalachia reported that their experience of loneliness is related to negative emotions including fear, anger, and worry. They reported that loneliness can lead to loss of function or independence [38]. Conversely, these women reported positive emotions such as joy when the feeling of loneliness is absent. Participants of the qualitative studies on loneliness in Appalachia reported that staying busy and going out were important to the management of loneliness [38]. This paper presents the effectiveness of LISTEN, an intervention designed to target impaired cognitive processes associated with loneliness, on the psychosocial and physiological measures in a sample of lonely, chronically ill adults living in the communities of Appalachia.				
Tomita, 2012[66]	Critical Time Intervention (CTI) is a time-limited care coordination intervention, shown to reduce recurrent homelessness in this population following discharge from shelters (5) and psychiatric hospitals (6). This study examines the impact of CTI on occurrences of psychiatric re-hospitalization among 150 previously homeless individuals with severe mental illness following discharge from inpatient treatment at two large state-operated hospitals.<br><br>While a growing body of research has begun to measure the effectiveness of a variety of outreach, housing and treatment approaches in meeting the complex needs of persons with mental illness, who are homeless or residentially unstable, few carefully documented models intended to reduce the incidence	1. Phase one--transition to the community--focuses on providing intensive support and assessing the resources that exist for the transition of care to community providers. Activities include: CTI worker makes home visits; Accompanies clients to community providers; Meets with caregivers; Supplements the role of caregivers when necessary; Gives support and advice to client and caregivers; Mediates conflicts between client and caregivers.	Yes	Prevalence of social need(s) in the community	Justification not specific to individual components

© 2024 Viswanathan M et al. *JAMA Network Open.*

Author, Year	Justification for intervention from publication[a]	Intervention description	Multiple Intervention Comp-onents	Was the intervention developed to respond to community or individual social need(s)?	Justification for specific components
	of homelessness in this population have been described or tested	2. Phase two try out is devoted to testing and adjusting the systems of support that were developed during phase one. Activities include: CTI worker observes operation of support network; Helps to modify network as necessary; Intervenes when a crisis arises.<br><br>3. Phase three transfer of care focuses on completing the transfer of responsibility to community resources that will provide long-term support. Activities include: CTI worker reaffirms ongoing roles of support network members; Develops and begins to set in motion plan for long-term goals (eg ,employment, education, family reunification); Holds party/meetings to symbolize transfer of care.			
Toro, 1997[67]	A vast array of shelters, food programs, and other emergency services now exists to serve the homeless population (Shlay & Rossi, 1992), and psychologists and other mental health professionals are becoming increasingly involved with designing and delivering services to address the many needs of homeless persons. In the past several years, grant funds have become increasing available, and many new interventions for the homeless have been implemented (Levine and Rog 1990; National Institute on Alcohol Abuse and Alcoholism [NIAAA] 1991). Unfortunately, few of these interventions have been formally evaluated and even fewer have been	1. Intensive case management, offering access and linkage to services (eg, financial aid, housing support, counseling for drug and alcohol problems, mental health assessment and treatment, and job training).<br><br>2. If an appropriate service could not be identified in the community, intervention staff would provide it.	Yes	Prevalence of social need(s) in the community	Justification not specific to individual components

Author, Year	Justification for intervention from publication[a]	Intervention description	Multiple Intervention Comp-onents	Was the intervention developed to respond to community or individual social need(s)?	Justification for specific components
	considered in experimental designs. Rigorous experimental evaluations are required if we are to identify truly effective interventions. The earnest group of experimental evaluations of programs for the homeless population involved substance abusers. Mercier, Bournier, and Pelandeau (1992) reviewed a group of seven experimental studies that focused on the treatment of skid-row alcoholics and that were all conducted in the period following the decriminalization of public inebriation and the development of extensive detoxification and related treatment approaches in the 1970s. Generally, the results of these studies were not encouraging when considering alcohol consumption and related outcomes. However, some evaluations showed positive effects on social adjustment, arrests, access to healthcare, financial and residential stability, or other outcomes. In what was probably the first field experiment to evaluate a program for homeless mentally ill persons, Lipton, Nutt, and Sabatini (1988) studied 49 seriously mentally ill homeless adults admitted to the psychiatric emergency service of New York's Bellevue Hospital. All study participants had been continuously homeless for at least 3 months before admission. Patients were randomly assigned to "routine discharge planning" or to an experimental post-discharge residential program that included "individualized case management, coordination of public assistance or Social Security benefits, medication monitoring, money management, meals, activity therapy, and, when appropriate, referrals to psychosocial and rehabilitation programs" (Lipton et al 1988, p. 41). Data available on 34 (69%) patients of the original sample at the final 12-month follow-up indicated fewer nights in hospitals or undomiciled and greater satisfaction with their living arrangements for the experimental group. In another field experiment, Morse, Calsyn, Allen, Tempelhoff, and Smith	3. The program also addressed the clients' immediate tangible needs. For example, funds were sometimes loaned to cover the security deposit for a new apartment and program staff helped clients obtain donated furniture and appliances and find quality day care for their children.			

Author, Year	Justification for intervention from publication[a]	Intervention description	Multiple Intervention Comp-onents	Was the intervention developed to respond to community or individual social need(s)?	Justification for specific components
	compared three interventions for seriously mentally ill homeless adults: (a) traditional outpatient mental health treatment; (b) daytime drop-in centers offering food, recreational facilities, and referrals; and (c) a "continuous treatment team" (1992, p. 1005) that provided therapeutic services to help clients cope with personal problems, link with psychiatric and other services, and learn community living skills. This study improved upon the Lipton et al (1988) study by including a larger sample (178 at baseline), a wider array of measures, and a more representative group of mentally ill persons (including those homeless for short periods and who had not been hospitalized). There was a good overall 12-month follow-up rate (57%), with a significantly better rate among those in the intensive intervention group (71%). Compared with the other two groups, those in the continuous treatment condition spent less time homeless during the follow-up year, used more community services, and were more satisfied with the services they received. A final experimental study involved street youths (ages 13 to 21) in Seattle (Cauce et al 1994). A sample of 300 youths was randomly assigned to “intensive” or “regular" case management. The intensive intervention was similar to that used by Morse et al (1992) although it was adapted for its younger population, and the research also included a broad array of measures and obtained a good rate of follow-up (68% after 1 year). Preliminary data at the first 3-month follow-up suggested several trends favoring the intensive over the regular case management groups (including changes on externalizing behaviors and self-reported quality of life).The present research used a field experiment methodology to evaluate an innovative intervention which, unlike those described above, was designed for the full range of homeless adults, including those with diagnoses of substance abuse and mental illness				

© 2024 Viswanathan M et al. *JAMA Network Open.*

Author, Year	Justification for intervention from publication[a]	Intervention description	Multiple Intervention Comp-onents	Was the intervention developed to respond to community or individual social need(s)?	Justification for specific components
	and those homeless with children. The intensive case management intervention evaluated here is similar to that studied by Morse et al (1992) and Cauce et al (1994), although it was adapted for its broader sample and attempted to affect a broader array of domains (eg, employment, housing, and health services, in addition to mental health).				
Towfighi, 2021[68]	The chronic care model (CCM), which incorporates self-management support, delivery system redesign, clinical information systems with decision support for applying evidence-based care guidelines, healthcare system leadership engagement, and community resources, has been effective in improving outcomes while reducing costs for chronic conditions.7-10 A randomized clinical trial of a CCM-based intervention by Cheng et al11 did not show a benefit beyond usual care in blood pressure (BP) reduction in a predominantly Hispanic population treated for stroke in a safety-net setting (ie, healthcare setting where all individuals receive care, regardless of health insurance status or ability to pay). The intervention, delivered solely within the healthcare system by advanced practice clinicians (APCs; including nurse practitioners or physician assistants), did not address home and community barriers to lifestyle change or transportation barriers to accessing care. We hypothesized that community health workers (CHWs) could more effectively address lifestyle factors, health literacy, medication adherence, and obstacles to behavior change. Therefore, we developed a multilevel, multicomponent, complex CCM-based team (including an APC, a CHW, and a physician) intervention for improving poststroke risk factor control.12<br><br>The Chronic Care Model (CCM), advocated as a guide for developing care improvement interventions for patients with chronic disease, has been an	1. Advanced practice clinicians: prescribe and titrate medications, emphasize medication adherence, and reinforce self-management skills<br><br>2. CHWs: assess and address medication adherence, provide stroke education and self-management training, liaison with the healthcare system<br><br>3. Home visits and telephone visits<br><br>4. Protocol-driven risk factor management<br><br>5. Educational materials and Chronic Disease Self-Management Program workshops<br><br>6. Resources or referrals to address social determinants of health	Yes	Community advisory board or other community input	Justification not specific to individual components

© 2024 Viswanathan M et al. *JAMA Network Open.*

Author, Year	Justification for intervention from publication[a]	Intervention description	Multiple Intervention Comp-onents	Was the intervention developed to respond to community or individual social need(s)?	Justification for specific components
	effective framework for multi-component intervention programs aimed at improving care processes and outcomes while reducing costs for various chronic conditions, including diabetes and hypertension [16, 17]. The six components of this model are self-management support, clinical information systems, delivery system redesign, decision support for application of evidence-based care guidelines, health care organization championship and leadership support, and community resources. We recently completed a different randomized controlled trial (RCT) of a CCM-based intervention for secondary stroke prevention, Systemic Use of STroke Averting INterventions (SUSTAIN) [18]. This experience highlighted our need for a strong community-based component to intensively address behavioral risk factors by accounting for social determinants of health, such as the social and community context, living situation, physical environment, education, and access to healthcare.				
Waitzkin, 2011[69]	When patients who present to primary care settings receive screening, the prevalence of depression generally ranges from 20 to 50%. These prevalence rates vary considerably according to setting, method of assessment, language used, and race/ethnicity [7–12]. Intervention trials for psychiatric disorders in large primary care settings such as managed care organizations [13– 18] have included improved depression treatment by systems modification or quality improvement programs to foster evidence-based care [19–23]. In the Partners in Care study, guideline-informed interventions resulted in improved quality of care, quality of life, clinical outcomes, and employment retention; cost-effectiveness analysis also showed substantial benefits [24–26]. Enhanced depression care for minorities has led to long-term improvements in outcomes [27]. Most intervention strategies include guideline- informed ''best practices''	1. Promotora interviews patient on contextual sources of depression (unemployment or under-employment, housing, food, trauma) using Factors Associated with Depression instrument.<br><br>2. Promotora then assists the patient to deal with the identified contextual sources of depression by mobilizing resources from the resource list and by providing other help at the promotora's discretion in relation to these problems only. Promotora documents activities in patient's chart.	Yes	Other, Extensive review of interventions designed to modify social contextual conditions that are risk factors for mental illness in primary care. Developed conceptual framework using biopsychosocial approach to depression.	Yes

© 2024 Viswanathan M et al. *JAMA Network Open.*

Author, Year	Justification for intervention from publication[a]	Intervention description	Multiple Intervention Comp-onents	Was the intervention developed to respond to community or individual social need(s)?	Justification for specific components
	for recognition and treatment of depression [28–32]. Recent intervention research demonstrates the value of enhanced, collaborative approaches [33–41]. Several studies substantiate the efficacy of collaborative interventions for depression in primary care for ethnically diverse and underserved populations [42–48]. Nevertheless, disparities remain in the care of patients treated in primary care settings, especially for minorities [49]. Promotoras have become a widely adopted work role in underserved communities [50, 51]. Our definition of promotora refers to her or his role as a trusted community member, who provides health-related services for underserved individuals in community settings and helps fortify the relationship between patients and PCPs [52–55]. Community health workers are known by nearly 30 titles such as: promotoras de salud (Spanish for ‘‘health promoters’’), community health advocates, outreach workers, indigenous health workers, lay health educators, and community health aides [56, 57]. Other than mental health services, promotoras have performed a variety of duties: first aid, nutrition education, blood pressure screenings, midwifery, translation, environmental work, patient transportation, case management, breast cancer screening, diabetes education, asthma management, social work, and peer counseling [58–61]. The Diabetes Initiative of the Robert Wood Johnson Foundation has included promotoras focusing in part on depression [62, 63]. Promotoras may help PCPs to identify patients' health needs and to consider the cultural relevance of treatments provided [64]. Researchers have assessed the efficacy of promotora interventions focusing on heart disease [65], diabetes [66], tobacco [67], general chronic diseases [68], breast and cervical cancer [69, 70], and nutrition [71]. These studies generally showed favorable intervention effects. Regarding applicability to diverse	3. Promotora communicates at every 2 months with depressed patients at the intervention community health centers and reports findings by standard form to be placed in chart for PCP to review.			

© 2024 Viswanathan M et al. *JAMA Network Open.*

Author, Year	Justification for intervention from publication[a]	Intervention description	Multiple Intervention Comp-onents	Was the intervention developed to respond to community or individual social need(s)?	Justification for specific components
	cultures and ethnicities, studies in Panama [72], Uganda [73], and Chile [74] showed positive results from training non-physicians for depression interventions in rural settings. A curriculum ‘‘toolbox’’ was developed for promotoras to use for English and Spanish speaking diabetic patients with depression [75].				
Williams, 2006[70]	Interventions aimed at modifying the risk factors for asthma have found that a multipronged and individually customized approach can improve outcomes for asthma.6'7 Use of an asthma counselor or case manager to deliver the intervention has emerged as a key component to implementing and delivering secondary asthma prevention measures.8'9 Programs that improve care, educate, control the exposure of children to allergens and irritants, and reduce exacerbation of symptoms decrease medical costs related to asthma.'0 Appropriate case management and decreasing exposure to environmental factors may reduce the frequency of acute exacerbations of asthma symptoms.11	1. Baseline environmental evaluations of the home.<br><br>2. Strategies to reduce exposure to dust mites and cockroaches, including encasing the mattress, box spring and pillows; instruction on cleaning procedures for carpets and fabrics; hydramethylnon gel for cockroach eradication; education about proper food-handling.<br><br>3. One-time professional cleaning of homes.<br><br>4. Instructions on other customized interventions based on the home audits and referrals as needed to resources available in the community.<br><br>5. Education about environmental tobacco smoke and other health education to support the environmental interventions (provided at the 2, 6, and 10 month visits).	Yes	Prevalence of social need(s) in the community	Yes

© 2024 Viswanathan M et al. *JAMA Network Open.*

Author, Year	Justification for intervention from publication[a]	Intervention description	Multiple Intervention Comp-onents	Was the intervention developed to respond to community or individual social need(s)?	Justification for specific components
Wu, 2019[71]	Despite the potential for improved care and cost savings from coordination between CBOs and healthcare organizations, there are barriers to developing and maintaining these partnerships.15−18 Clinical providers are often unaware of local community resources to which they might refer. Similarly, CBO staff members struggle to identify the specific channels into the local healthcare system to meet their clients' needs. Accordingly, the team developed and implemented an RCT, Baltimore Community-based Organizations Neighborhood Network: Enhancing Capacity Together (CONNECT) to evaluate how a community engagement approach could be used to codevelop a set of interventions to link a local health system (Johns Hopkins Health System [JHHS]) and surrounding CBOs. The overarching aim was to enhance the capacity of both CBO staff and frontline hospital workers to address client needs by strengthening the bidirectional flow of information about health and social services and building networks that span both entities. Outcomes were examined in a cohort of JHHS patients, CBO clients, CBO staff, and JHHS frontline staff.	1. Baltimore Community-based Organizations Neighborhood Network: Enhancing Capacity Together website<br><br>2. Paid subscription to Healthify<br><br>3. Designated research assistant who provided technical assistance and served as liaisons between the study team and community-based organizations<br><br>4. 5 in-person meet-and-greet sessions between community-based organizations leaders and JHHHS frontline staff to increase awareness of services, establish personal contacts, and promote referrals	Yes	Community advisory board or other community input	Justification not specific to individual components
Zulman, 2017[72]	Intensive outpatient care programs have gained traction in recent years as a model for delivering comprehensive, individualized medical, mental health, and social services to high-need patients.1,8-11 Despite enthusiasm for these programs,12 evidence regarding their effects on hospitalization and costs is mixed.13,14 Some have described program savings as high as 15% to 50% in observational and retrospective evaluations,15-18 and there are examples of programs for older adults that have decreased acute care use and/or costs in randomized trials.19-22However, rigorous evaluations of other programs have not demonstrated these effects,23-25 suggesting that contextual factors such as patient population, existing services, and resource availability	1. Comprehensive patient assessment and goal setting, including assessment of physical function, cognitive impairment, social support, advance directives, medication adherence, and level of activation.<br><br>2. Multidisciplinary team (nurse practitioner, physician, social worker, and recreation therapist) that partners with and augments patients medical home to provide enhanced	Yes	Prevalence of social need(s) in the community	Justification not specific to individual components

© 2024 Viswanathan M et al. *JAMA Network Open.*

Author, Year	Justification for intervention from publication[a]	Intervention description	Multiple Intervention Comp-onents	Was the intervention developed to respond to community or individual social need(s)?	Justification for specific components
	may be important. Because many early intensive outpatient programs were designed specifically for older adults,19-22 patients in fragmented safety-net settings,11,13 and employed individuals,17,18 their evaluations may not be generalizable to integrated systems caring for more diverse populations. Furthermore, it is unclear whether individuals receiving comprehensive care in an effective patient-centered medical home(PCMH)26 will benefit from additional intensive services. To advance understanding of the value of intensive outpatient care in these settings, we evaluated a program in the Veterans Affairs (VA) Health Care System, an integrated system with a well-established PCMH. The program was a quality improvement initiative offered to a random sample of patients with high healthcare costs and/or hospitalization risk. In this article, we describe the program's effects on healthcare utilization, cost, and patient experience.	services, including care management for medical and social service needs, frequent contact via phone and in-person, coordination of care with VA and non-VA clinicians, tracking system to monitor ED visits, hospitalizations, and upcoming appointments.<br><br>3. Interventions to support patients and caregivers quality of life (eg, recreation therapy).<br><br>4. Attendance at specialty care appointments to support decision-making, when appropriate.			

[a] The text in this column was taken directly from the publication referenced in the first column.

AAP = American Academy Pediatrics; CHIP = Children's Health Insurance Program; CHW = community health worker; CSA = community-supported agriculture; CTI = critical time intervention; ED = emergency department; EYIPP = Empowering Youth Through Interpersonal Violence Prevention Program FQHC = Federally Qualified Health Centers; NICU = neonatal intensive care unit; NP= nurse practioners; PCP = primary care provider; SNAP = Supplemental Nutrition Assistance Program; TC = transitional care; VA = Veterans Affairs.

© 2024 Viswanathan M et al. *JAMA Network Open.*

eReferences

1. Andrews KG, Martin MW, Shenberger E, Pereira S, Fink G, McConnell M. Financial support to Medicaid-eligible mothers increases caregiving for preterm infants. *Matern Child Health J*. 2020;24(5):587-600. doi:10.1007/s10995-020-02905-7
2. Balaban RB, Zhang F, Vialle-Valentin CE, et al. Impact of a patient navigator program on hospital-based and outpatient utilization over 180 days in a safety-net health system. *J Gen Intern Med*. 2017;32(9):981-989. doi:10.1007/s11606-017-4074-2
3. Berkowitz SA, O'Neill J, Sayer E, et al. Health center-based community-supported agriculture: an RCT. *Am J Prev Med*. 2019;57(6 Suppl 1):S55-S64. doi:10.1016/j.amepre.2019.07.015
4. Birkhead GS, LeBaron CW, Parsons P, et al. The immunization of children enrolled in the Special Supplemental Food Program for Women, Infants, and Children (WIC). The impact of different strategies. *JAMA*. 1995;274(4):312-6.
5. Bovell-Ammon A, Mansilla C, Poblacion A, et al. Housing intervention for medically complex families associated with improved family health: pilot randomized trial. *Health Aff (Millwood)*. 2020;39(4):613-621. doi:10.1377/hlthaff.2019.01569
6. Bronstein LR, Gould P, Berkowitz SA, James GD, Marks K. Impact of a social work care coordination intervention on hospital readmission: a randomized controlled trial. *Soc Work*. 2015;60(3):248-55.
7. Brown DM, Hernandez EA, Levin S, et al. Effect of social needs case management on hospital use among adult Medicaid beneficiaries: a randomized study. *Ann Intern Med*. 2022;175(8):1109-1117. doi:10.7326/M22-0074
8. Bryce R, Wolfson Bryce JA, Cohen Bryce A, et al. A pilot randomized controlled trial of a fruit and vegetable prescription program at a federally qualified health center in low income uncontrolled diabetics. *Prev Med Rep* 2021;23:101410. doi:10.1016/j.pmedr.2021.101410
9. Burnam MA, Morton SC, McGlynn EA, et al. An experimental evaluation of residential and nonresidential treatment for dually diagnosed homeless adults. *J Addict Dis*. 1995;14(4):111-34. doi:10.1300/j069v14n04_07
10. Carter J, Hassan S, Walton A, Yu L, Donelan K, Thorndike AN. Effect of community health workers on 30-day hospital readmissions in an accountable care organization population: a randomized clinical trial. *JAMA Netw Open*. 2021;4(5):e2110936. doi:10.1001/jamanetworkopen.2021.10936
11. Wagner V, Sy J, Weeden K, et al. Effectiveness of intensive case management for homeless adolescents: results of a 3-month follow-up. *J Emot Behav Disord*. 2016;2(4):219-227. doi:10.1177/106342669400200404
12. Cheng TL, Haynie D, Brenner R, Wright JL, Chung SE, Simons-Morton B. Effectiveness of a mentor-implemented violence prevention intervention for assault-injured youth presenting to the emergency department: results of a randomized trial. *Pediatrics*. 2008;122(5):938-46. this is a duplicate of 11865; we retained this study bc the extraction data were more complete. doi:10.1542/peds.2007-2096
13. Corrigan PW, Kraus DJ, Pickett SA, et al. Using peer navigators to address the integrated health care needs of homeless African Americans with serious mental illness. *Psychiatr Serv*. 2017;68(3):264-270. doi:10.1176/appi.ps.201600134

14. Counsell SR, Callahan CM, Clark DO, et al. Geriatric care management for low-income seniors: a randomized controlled trial. *JAMA*. 2007;298(22):2623-33. doi:10.1001/jama.298.22.2623
15. Cox GB, Walker RD, Freng SA, Short BA, Meijer L, Gilchrist L. Outcome of a controlled trial of the effectiveness of intensive case management for chronic public inebriates. *J Stud Alcohol*. 1998;59(5):523-32. doi:10.15288/jsa.1998.59.523
16. Dixon L, Goldberg R, Iannone V, et al. Use of a critical time intervention to promote continuity of care after psychiatric inpatient hospitalization. *Psychiatr Serv*. 2009;60(4):451-8. doi:10.1176/ps.2009.60.4.451
17. Duncan PW, Bushnell CD, Jones SB, et al. Randomized pragmatic trial of stroke transitional care: the COMPASS Study. *Circ Cardiovasc Qual Outcomes*. 2020;13(6):e006285. doi:10.1161/CIRCOUTCOMES.119.006285
18. Eismann EA, Zhang B, Fenchel M, et al. Impact of screening and co-located parent coaching within pediatric primary care on child health care use: a stepped wedge design. *Prev Sci*. 2023;24(1):173-185. doi:10.1007/s11121-022-01447-4
19. Ell K, Aranda MP, Wu S, Oh H, Lee PJ, Guterman J. Promotora assisted depression and self-care management among predominantly Latinos with concurrent chronic illness: safety net care system clinical trial results. *Contemp Clin Trials*. 2017;61:1-9. doi:10.1016/j.cct.2017.07.001
20. Ferrer RL, Neira LM, De Leon Garcia GL, Cuellar K, Rodriguez J. Primary care and food bank collaboration to address food insecurity: a pilot randomized trial. *Nutr Metab Insights*. 2019;12:1178638819866434. doi:10.1177/1178638819866434
21. Finkelstein A, Zhou A, Taubman S, Doyle J. Health care hotspotting—a randomized, controlled trial. *N Engl J Med*. 2020;382(2):152-162. doi:10.1056/NEJMsa1906848
22. Gottlieb LM, Adler NE, Wing H, et al. Effects of in-person assistance vs personalized written resources about social services on household social risks and child and caregiver health: a randomized clinical trial. *JAMA Netw Open*. 2020;3(3):e200701. doi:10.1001/jamanetworkopen.2020.0701
23. Gottlieb LM, Hessler D, Long D, et al. Effects of social needs screening and in-person service navigation on child health: a randomized clinical trial. *JAMA Pediatr*. 2016:e162521. doi:10.1001/jamapediatrics.2016.2521
24. Guevara JP, Erkoboni D, Gerdes M, et al. Effects of early literacy promotion on child language development and home reading environment: a randomized controlled trial. *J Pediatr X*. 2020;2:100020. doi:10.1016/j.ympdx.2020.100020
25. Hannan J, Brooten D, Page T, Galindo A, Torres M. Low-income first-time mothers: effects of APN follow-up using mobile technology on maternal and infant outcomes. *Glob Pediatr Health*. 2016;3:2333794X16660234. April2023 update: cochrane access to care. doi:10.1177/2333794X16660234
26. Heisler M, Lapidos A, Kieffer E, et al. Impact on health care utilization and costs of a Medicaid community health worker program in Detroit, 2018–2020: a randomized program evaluation. *Am J Public Health*. 2022;112(5):766-775. doi:10.2105/ajph.2021.306700
27. Henschen BL, Theodorou ME, Chapman M, et al. An intensive intervention to reduce readmissions for frequently hospitalized patients: the CHAMP Randomized Controlled Trial. *J Gen Intern Med*. 2022;37(8):1877-1884. doi:10.1007/s11606-021-07048-1

© 2024 Viswanathan M et al. *JAMA Network Open.*

28. Herman D, Opler L, Felix A, Valencia E, Wyatt RJ, Susser E. A critical time intervention with mentally ill homeless men: impact on psychiatric symptoms. *J Nerv Ment Dis*. 2000;188(3):135-40. doi:10.1097/00005053-200003000-00002
29. Hilgeman MM, Mahaney-Price AF, Stanton MP, et al. Alabama Veterans Rural Health Initiative: a pilot study of enhanced community outreach in rural areas. *J Rural Health*. 2014;30(2):153-63. doi:10.1111/jrh.12054
30. Horwitz SM, Busch SH, Balestracci KM, Ellingson KD, Rawlings J. Intensive intervention improves primary care follow-up for uninsured emergency department patients. *Acad Emerg Med*. 2005;12(7):647-52. doi:10.1197/j.aem.2005.02.015
31. Johnson EM, Poleshuck E, Possemato K, et al. Practical and emotional peer support tailored for life's challenges: personalized support for progress randomized clinical pilot trial in a Veterans Health Administration Women's Clinic. *Mil Med*. 2022; SIREN import (2-2023). doi:10.1093/milmed/usac164
32. Kangovi S, Mitra N, Grande D, et al. Patient-centered community health worker intervention to improve posthospital outcomes: a randomized clinical trial. *JAMA Intern Med*. 2014;174(4):535-43. doi:10.1001/jamainternmed.2013.14327
33. Kangovi S, Mitra N, Grande D, Huo H, Smith RA, Long JA. Community health worker support for disadvantaged patients with multiple chronic diseases: a randomized clinical trial. *Am J Public Health*. 2017;107(10):1660-1667. doi:10.2105/ajph.2017.303985
34. Kangovi S, Mitra N, Norton L, et al. Effect of community health worker support on clinical outcomes of low-income patients across primary care facilities: a randomized clinical trial. *JAMA Intern Med*. 2018;178(12):1635-1643. doi:10.1001/jamainternmed.2018.4630
35. Kelley L, Capp R, Carmona JF, et al. Patient navigation to reduce emergency department (ED) utilization among Medicaid insured, frequent ED users: a randomized controlled trial. *J Emerg Med*. 2020;58(6):967-977. doi:10.1016/j.jemermed.2019.12.001
36. Kempainen S, Cutts DB, Robinson-O'Brien R, De Kesel Lofthus A, Gilbertson DT, Mino R. A collaborative pilot to support patients with diabetes through tailored food box home delivery. *Health Promot Pract*. 2023:15248399221100792. April2023: Siren import. doi:10.1177/15248399221100792
37. Kim SE, Michalopoulos C, Kwong RM, Warren A, Manno MS. Telephone care management's effectiveness in coordinating care for Medicaid beneficiaries in managed care: a randomized controlled study. *Health Serv Res*. 2013;48(5):1730-49. doi:10.1111/1475-6773.12060
38. Kneipp SM, Kairalla JA, Lutz BJ, et al. Public health nursing case management for women receiving temporary assistance for needy families: a randomized controlled trial using community-based participatory research. *Am J Public Health*. 2011;101(9):1759-68. ref from SR doi:10.2105/ajph.2011.300210
39. Korr WS, Joseph A. Housing the homeless mentally ill: findings from Chicago. *J Soc Serv Res*. 2008;21(1):53-68. doi:10.1300/J079v21n01_04
40. Krieger J, Collier C, Song L, Martin D. Linking community-based blood pressure measurement to clinical care: a randomized controlled trial of outreach and tracking by community health workers. *Am J Public Health*. 1999;89(6):856-61. doi:10.2105/ajph.89.6.856
41. Krieger J, Takaro TK, Song L, Beaudet N, Edwards K. A randomized controlled trial of asthma self-management support comparing clinic-based nurses and in-home community

© 2024 Viswanathan M et al. *JAMA Network Open.*

health workers: the Seattle-King County Healthy Homes II Project. *Arch Pediatr Adolesc Med*. 2009;163(2):141-9. doi:10.1001/archpediatrics.2008.532
42. Krieger J, Song L, Philby M. Community health worker home visits for adults with uncontrolled asthma: the HomeBASE Trial randomized clinical trial. *JAMA Intern Med*. 2015;175(1):109-17. doi:10.1001/jamainternmed.2014.6353
43. Lin MP, Blanchfield BB, Kakoza RM, et al. ED-based care coordination reduces costs for frequent ED users. *Am J Manag Care*. 2017;23(12):762-766.
44. Lipton FR, Nutt S, Sabatini A. Housing the homeless mentally ill: a longitudinal study of a treatment approach. *Hosp Community Psychiatry*. 1988;39(1):40-5. doi:10.1176/ps.39.1.40
45. Liss DT, Ackermann RT, Cooper A, et al. Effects of a transitional care practice for a vulnerable population: a pragmatic, randomized comparative effectiveness trial. *J Gen Intern Med*. 2019;34(9):1758-1765. doi:10.1007/s11606-019-05078-4
46. Lopez MA, Yu X, Hetrick R, et al. Social needs screening in hospitalized pediatric patients: a randomized controlled trial. *Hosp Pediatr*. 2023;13(2):95-114. SIREN import (2-2023). doi:10.1542/hpeds.2022-006815
47. Mackinney T, Visotcky AM, Tarima S, Whittle J. Does providing care for uninsured patients decrease emergency room visits and hospitalizations? *J Prim Care Community Health*. 2013;4(2):135-42. doi:10.1177/2150131913478981
48. McClintock HF, Bogner HR. Incorporating patients' social determinants of health into hypertension and depression care: a pilot randomized controlled trial. *Community Ment Health J*. 2017;53(6):703-710.
49. Melnikow J, Paliescheskey M, Stewart GK. Effect of a transportation incentive on compliance with the first prenatal appointment: a randomized trial. *Obstet Gynecol*. 1997;89(6):1023-7. doi:10.1016/s0029-7844(97)00147-6
50. Mion LC, Palmer RM, Meldon SW, et al. Case finding and referral model for emergency department elders: a randomized clinical trial. *Ann Emerg Med*. 2003;41(1):57-68. doi:10.1067/mem.2003.3
51. Nyamathi A, Flaskerud JH, Leake B, Dixon EL, Lu A. Evaluating the impact of peer, nurse case-managed, and standard HIV risk-reduction programs on psychosocial and health-promoting behavioral outcomes among homeless women. *Res Nurs Health*. 2001;24(5):410-22. doi:10.1002/nur.1041
52. O'Brien GM, Stein MD, Fagan MJ, Shapiro MJ, Nasta A. Enhanced emergency department referral improves primary care access. *Am J Manag Care*. 1999;5(10):1265-9.
53. O'Connell M, Sint K, Rosenheck R. How do housing subsidies improve quality of life among homeless adults? A mediation analysis. *Am J Community Psychol*. 2018;61(3-4):433-444. doi:10.1002/ajcp.12229
54. O'Toole TP, Johnson EE, Borgia ML, Rose J. Tailoring outreach efforts to increase primary care use among homeless veterans: results of a randomized controlled trial. *J Gen Intern Med*. 2015;30(7):886-98. doi:10.1007/s11606-015-3193-x
55. Post B, Lapedis J, Singh K, et al. Predictive model-driven hotspotting to decrease emergency department visits: a randomized controlled trial. *J Gen Intern Med*. 2021;36(9):2563-2570. doi:10.1007/s11606-021-06664-1
56. Raven MC, Niedzwiecki MJ, Kushel M. A randomized trial of permanent supportive housing for chronically homeless persons with high use of publicly funded services. *Health Serv Res*. 2020;55 Suppl 2:797-806. doi:10.1111/1475-6773.13553

© 2024 Viswanathan M et al. *JAMA Network Open.*

57. Sadowski LS, Kee RA, VanderWeele TJ, Buchanan D. Effect of a housing and case management program on emergency department visits and hospitalizations among chronically ill homeless adults: a randomized trial. *JAMA*. 2009;301(17):1771-8. doi:10.1001/jama.2009.561
58. Schickedanz A, Perales L, Holguin M, et al. Clinic-based financial coaching and missed pediatric preventive care: a randomized trial. *Pediatrics*. 2023;151(3)April 2023 update: medline food insec. doi:10.1542/peds.2021-054970
59. Schumacher JR, Lutz BJ, Hall AG, et al. Feasibility of an ED-to-home intervention to engage patients: a mixed-methods investigation. *West J Emerg Med*. 2017;18(4):743. doi:10.5811/westjem.2017.2.32570
60. Sege R, Preer G, Morton SJ, et al. Medical-legal strategies to improve infant health care: a randomized trial. *Pediatrics*. 2015;doi:10.1542/peds.2014-2955
61. Shinn M, Samuels J, Fischer SN, Thompkins A, Fowler PJ. Longitudinal impact of a family critical time intervention on children in high-risk families experiencing homelessness: a randomized trial. *Am J Community Psychol*. 2015;56(3-4):205-16. doi:10.1007/s10464-015-9742-y
62. Shumway M, Boccellari A, O'Brien K, Okin RL. Cost-effectiveness of clinical case management for ED frequent users: results of a randomized trial. *Am J Emerg Med*. 2008;26(2):155-64. doi:10.1016/j.ajem.2007.04.021
63. Sood RK, Bae JY, Sabety A, Chan PY, Heindrichs C. ActionHealthNYC: effectiveness of a health care access program for the uninsured, 2016–2017. *Am J Public Health*. 2021;111(7):1318-1327. doi:10.2105/AJPH.2021.306271
64. Talavera GA, Castaneda SF, Mendoza PM, et al. Latinos understanding the need for adherence in diabetes (LUNA-D): a randomized controlled trial of an integrated team-based care intervention among Latinos with diabetes. *Transl Behav Med*. 2021;doi:10.1093/tbm/ibab052
65. Theeke LA, Mallow JA, Moore J, McBurney A, Rellick S, VanGilder R. Effectiveness of LISTEN on loneliness, neuroimmunological stress response, psychosocial functioning, quality of life, and physical health measures of chronic illness. *Int J Nurs Sci*. 2016;3(3):242-251. doi:10.1016/j.ijnss.2016.08.004
66. Tomita A, Herman DB. The impact of critical time intervention in reducing psychiatric rehospitalization after hospital discharge. *Psychiatr Serv*. 2012;63(9):935-7. doi:10.1176/appi.ps.201100468
67. Toro PA, Passero Rabideau JM, Bellavia CW, et al. Evaluating an intervention for homeless persons: results of a field experiment. *J Consult Clin Psychol*. 1997;65(3):476-84. doi:10.1037//0022-006x.65.3.476
68. Towfighi A, Cheng EM, Ayala-Rivera M, et al. Effect of a coordinated community and chronic care model team intervention vs usual care on systolic blood pressure in patients with stroke or transient ischemic attack: the SUCCEED Randomized Clinical Trial. *JAMA Netw Open*. 2021;4(2):e2036227. doi:10.1001/jamanetworkopen.2020.36227
69. Waitzkin H, Getrich C, Heying S, et al. Promotoras as mental health practitioners in primary care: a multi-method study of an intervention to address contextual sources of depression. *J Community Health*. 2011;36(2):316-31. doi:10.1007/s10900-010-9313-y
70. Williams SG, Brown CM, Falter KH, et al. Does a multifaceted environmental intervention alter the impact of asthma on inner-city children? *J Natl Med Assoc*. 2006;98(2):249-60.

© 2024 Viswanathan M et al. *JAMA Network Open.*

71. Wu AW, Weston CM, Ibe CA, et al. The Baltimore Community-Based Organizations Neighborhood Network: Enhancing Capacity Together (CONNECT) Cluster RCT. *Am J Prev Med*. 2019;57(2):e31-e41. doi:10.1016/j.amepre.2019.03.013
72. Zulman DM, Pal Chee C, Ezeji-Okoye SC, et al. Effect of an intensive outpatient program to augment primary care for high-need Veterans Affairs patients: a randomized clinical trial. *JAMA Intern Med*. 2017;177(2):166-175. doi:10.1001/jamainternmed.2016.8021
73. Lewin S, Hendry M, Chandler J, et al. Assessing the complexity of interventions within systematic reviews: development, content and use of a new tool (iCAT_SR). *BMC Med Res Methodol*. 2017;17(1):76. doi:10.1186/s12874-017-0349-x
74. Lewin S, Hendry M, Chandler J, et al. *Guidance for using the iCAT_SR: Intervention Complexity Assessment Tool for Systematic Reviews, Version 1.0*. 2016. https://methods.cochrane.org/sites/methods.cochrane.org/files/uploads/icat_sr_additional_file_4_2016_12_27.pdf
75. Stirman SW, Miller CJ, Toder K, Calloway A. Development of a framework and coding system for modifications and adaptations of evidence-based interventions. *Implement Sci*. 2013;8:65. doi:10.1186/1748-5908-8-65
76. Evans T, Brown H. Road traffic crashes: operationalizing equity in the context of health sector reform. *Inj Control Saf Promot*. 2003;10(1-2):11-2. doi:10.1076/icsp.10.1.11.14117
77. Welch V, Petticrew M, Petkovic J, et al. Extending the PRISMA statement to equity-focused systematic reviews (PRISMA-E 2012): explanation and elaboration. *Intern*. 2015;14:92. doi:10.1186/s12939-015-0219-2
78. Berkowitz S, Delahanty L, Terranova J, et al. Medically tailored meal delivery for diabetes patients with food insecurity: a randomized cross-over trial. *J Gen Intern Med*. Article In Press. 2018;(no pagination). doi:10.1007/s11606-018-4716-z https://www.cochranelibrary.com/central/doi/10.1002/central/CN-01667976/full
79. High PC, LaGasse L, Becker S, Ahlgren I, Gardner A. Literacy promotion in primary care pediatrics: can we make a difference? *Pediatrics*. 2000;105(4):927-934. https://www.cochranelibrary.com/central/doi/10.1002/central/CN-01623599/full
80. Flores G, Lin H, Walker C, et al. Parent mentoring program increases coverage rates for uninsured Latino children. *Health Aff (Millwood)*. Randomized Controlled Trial; Research Support, N.I.H., Extramural. 2018;37(3):403-412. doi:10.1377/hlthaff.2017.1272 https://www.cochranelibrary.com/central/doi/10.1002/central/CN-01703839/full
81. Caskey R, Moran K, Touchette D, et al. Effect of comprehensive care coordination on Medicaid expenditures compared with usual care among children and youth with chronic disease: a randomized clinical trial. *JAMA Netw Open*. Randomized Controlled Trial; Research Support, U.S. Gov't, Non-P.H.S. 2019;2(10):e1912604. doi:10.1001/jamanetworkopen.2019.12604 https://www.cochranelibrary.com/central/doi/10.1002/central/CN-01996057/full
82. Lumba-Brown A, Batek M, Choi P, Keller M, Kennedy R. Mentoring pediatric victims of interpersonal violence reduces recidivism. *J Interpers Violence*. Journal: Article. 2020;35(21-22):4262-4275. doi:10.1177/0886260517705662 https://www.cochranelibrary.com/central/doi/10.1002/central/CN-02158727/full

https://journals.sagepub.com/doi/10.1177/0886260517705662?url_ver=Z39.88-2003&rfr_id=ori:rid:crossref.org&rfr_dat=cr_pub%3dpubmed

© 2024 Viswanathan M et al. *JAMA Network Open.*
