# Supplementary material for: Evaluating Intensity, Complexity, and Potential for Causal Inference in Social Needs Interventions: A Review of a Scoping Review
Source: JAMA Netw Open. 2024 Jun 21;7(6):e2417994. doi: 10.1001/jamanetworkopen.2024.17994 (PMC11193129; doi:10.1001/jamanetworkopen.2024.17994)
Supplement: Supplement 2. — Data Sharing Statement [file jamanetwopen-e2417994-s002.pdf]

Data Sharing Statement

Viswanathan. Evaluating Intensity, Complexity, and Potential for Causal Inference in Social Needs Interventions: A Systematic Review. *JAMA Netw Open*. Published June 21, 2024. doi:10.1001/jamanetworkopen.2024.17994

Data
Data available: Yes
Data types: Other (please specify)
Additional Information: Data available in manuscript and supplemental tables and on PCORI's website
How to access data: https://www.pcori.org/research-results/2020/scoping-review-and-evidence-map-social-needs-interventions-improve-health-outcomes
When available: With publication

Supporting Documents
Document types: None

Additional Information
Who can access the data: Anyone
Types of analyses: Any purpose
Mechanisms of data availability: Files can be freely downloadeded
